# Photoactivatable CRISPR/Cas13d via upconversion nanoparticles for deep tissue RNA engineering and orthopedic therapy

**DOI:** 10.1038/s41467-026-72181-6

**Published:** 2026-04-20

**Authors:** Jie Zhao, Jingyu Zhang, Miaomiao Gao, Zukang Miao, Yang Zhang, Yue Guo, Zhengrui Fan, Jinglin Tian, Lu Yang, Ning Jiang, Jianxiong Ma, Jun Jiao, Jinbin Pan, Xinlong Ma

**Affiliations:** 1 Department of Orthopedic, Tianjin Hospital, Tianjin University, Tianjin, China; 2 Department of Bone and Soft Tissue Oncology, Tianjin Hospital, Tianjin University, Tianjin, China; 3 Tianjin Key Laboratory of Orthopedic Biomechanics and Medical Engineering, Tianjin Hospital, Tianjin, China; 4 Tianjin Key Laboratory of Tumor Microenvironment and Neurovascular Regulation, School of Medicine, Nankai University, Tianjin, China; 5 Department of Radiology, Tianjin Key Lab of Functional Imaging & Tianjin Institute of Radiology, Tianjin Medical University General Hospital, Tianjin, China; 6 Athinoula A. Martinos Center for Biomedical Imaging, Department of Radiology, Massachusetts General Hospital, Harvard Medical School, Charlestown, MA USA

**Keywords:** Translational research, Nucleic-acid therapeutics

## Abstract

Spatiotemporal control of RNA therapeutics remains a fundamental challenge limiting clinical translation. Here, we develop a photoactivatable CRISPR/Cas13d (paCas13d) system that enables non-invasive, light-controlled RNA manipulation in deep tissues. Through structure-guided engineering, we identify optimal split sites within RfxCas13d and create light-switchable fragments using CRY2PHR/CIBN optogenetic dimerization. To overcome the limited tissue penetration of blue light, we engineer polyethylenimine-functionalized upconversion nanoparticles (UCNPs-PEI) that serve dual roles as gene carriers and photon transducers, converting tissue-penetrating near-infrared (NIR) to blue light. The UCNPs-PEI@paCas13d system achieves precise spatiotemporal control of RNA targeting within bone tissue in vivo. In a murine steroid-associated osteonecrosis model, NIR-activated paCas13d achieves robust TET3 knockdown, disrupting the TET3-5hmC-PTEN axis that drives glucocorticoid-induced osteocyte apoptosis. This targeted intervention prevents bone deterioration, with treated mice showing preserved trabecular architecture, enhanced bone volume, and favorable shifts in bone turnover markers, while maintaining systemic glucocorticoid efficacy. Our platform combines the programmability of CRISPR/Cas13d with non-invasive optical control, offering a versatile approach for treating diseases requiring localized RNA modulation while minimizing systemic effects.

## Abstract (web summary)

Researchers develop a light-switchable CRISPR platform using upconversion nanoparticles to enable RNA manipulation in deep tissues. Near-infrared activation triggers precise, localised RNA knockdown or editing, offering a versatile approach for diseases requiring targeted RNA modulation.

## Introduction

The emergence of CRISPR/Cas systems has fundamentally transformed our ability to manipulate genetic information with unprecedented precision. Within this revolutionary toolkit, the CRISPR/Cas13 system occupies a unique niche as an RNA targeting platform that offers distinct advantages over DNA-editing counterparts^1,2^. Unlike Cas9 or base editors that introduce permanent genomic alterations, Cas13 mediates transient and reversible modulation of gene expression at the post-transcriptional level, making it particularly suited for therapeutic applications where temporal control is paramount. Among the diverse Cas13 orthologs, RfxCas13d from *Ruminococcus flavefaciens* has emerged as one of the most compact variants, facilitating efficient delivery via size-constrained vectors while maintaining robust catalytic activity and high target specificity^3–5^.

The therapeutic potential of Cas13d is particularly compelling in the context of diseases characterized by aberrant RNA expression or processing. However, constitutive Cas13d activity following delivery poses significant challenges, including potential cytotoxicity from prolonged RNA degradation and inability to restrict therapeutic effects to specific tissues or time windows^3,6^. These limitations underscore the critical need for precise spatiotemporal control mechanisms that can harness the full therapeutic potential of Cas13d while minimizing adverse effects.

Optogenetics has emerged as a powerful approach for achieving such control, utilizing light-responsive proteins to enable non-invasive, reversible regulation of biological processes with exquisite spatial and temporal precision^7,8^. The integration of optogenetic control with CRISPR systems represents a convergence of two transformative technologies^9,10^. Among various optogenetic strategies, split-protein approaches offer unique advantages by dividing functional proteins into inactive fragments that reconstitute activity only upon light-induced dimerization^11^. This strategy not only provides stringent control over protein activity but also addresses delivery constraints by reducing the size of individual components. The cryptochrome 2 (CRY2) and CIB1 (cryptochrome-interacting basic helix-loop-helix 1) system represents one of the most well-characterized optogenetic dimerization platforms. Upon blue light exposure (~470 nm), the photolyase homology region of CRY2 (CRY2PHR) undergoes a conformational change that enables rapid association with CIBN (the N-terminal domain of CIB1), with dimerization occurring within seconds and reversal within minutes following light cessation^12^. This rapid and reversible interaction provides an ideal molecular switch for controlling split protein systems.

However, the clinical translation of optogenetic therapies faces a fundamental biophysical constraint: the limited tissue penetration of visible light. Blue light, while optimal for CRY2/CIBN activation, penetrates less than 1 mm into biological tissues due to strong absorption by hemoglobin, melanin, and water. This necessitates invasive approaches such as implanted optical fibers for deep tissue applications, significantly limiting clinical feasibility and patient acceptability. Upconversion nanoparticles (UCNPs) offer an elegant solution to this challenge^13^. These lanthanide-doped nanocrystals possess the unique ability to convert low-energy near-infrared (NIR) photons into higher-energy visible emissions through a non-linear optical process. NIR light in the optical window (650–1350 nm) can penetrate several centimeters into biological tissues with minimal absorption and scattering. By engineering UCNPs to emit blue light upon NIR excitation, we can effectively deliver optogenetic activation signals to deep tissues non-invasively. Furthermore, the surface functionalization of UCNPs with cationic polymers such as polyethylenimine (PEI) enables them to serve dual roles as both photon transducers and gene delivery vehicles, creating an integrated therapeutic platform.

Steroid-associated osteonecrosis (SAON) exemplifies a clinical scenario where spatially-targeted RNA modulation could provide significant therapeutic benefit. This devastating complication affects 10–30% of patients receiving high-dose glucocorticoid therapy, with the femoral head being the most commonly affected site^14^. The pathogenesis of SAON involves a complex cascade initiated by glucocorticoid-induced osteocyte apoptosis, leading to disruption of the lacunocanalicular network, impaired bone remodeling, and eventual subchondral collapse^15^. Recent mechanistic insights have revealed that the ten-eleven translocation 3 (TET3) enzyme plays a pivotal role in SAON pathogenesis^16,17^. TET3 catalyzes the oxidation of 5-methylcytosine to 5-hydroxymethylcytosine (5hmC), initiating active DNA demethylation^18^. In response to glucocorticoid exposure, TET3 expression is dramatically upregulated in osteocytes, leading to aberrant epigenetic reprogramming. This results in the demethylation and subsequent activation of PTEN, which antagonizes the pro-survival PI3K/Akt pathway. The resulting shift in the cellular survival/death balance triggers extensive osteocyte apoptosis, initiating the pathological cascade of SAON. The spatial specificity of SAON pathology—predominantly affecting weight-bearing joints while sparing systemic bone—presents an ideal therapeutic scenario for localized intervention. Current preventive strategies are limited and often ineffective, as systemic approaches would interfere with the beneficial anti-inflammatory and immunosuppressive effects of glucocorticoids. Therefore, a technology capable of selectively suppressing TET3 expression in at-risk bone tissue while preserving normal glucocorticoid signaling elsewhere could revolutionize SAON prevention.

In this study, we present a paCas13d system that integrates structure-guided protein engineering, optogenetic control, and upconversion nanotechnology to achieve spatially-restricted RNA knockdown in deep tissues. Through systematic screening of split sites within RfxCas13d, we identified optimal configurations that maintain catalytic efficiency while providing stringent light-dependent control. By combining this split paCas13d system with PEI-functionalized UCNPs, we demonstrate non-invasive, NIR-triggered RNA modulation in bone tissue. Application of this technology to target TET3 in a murine SAON model reveals its potential to prevent glucocorticoid-induced osteonecrosis while maintaining the systemic therapeutic effects of corticosteroids. This work establishes a versatile platform for spatiotemporally controlled RNA therapeutics with broad implications for treating diseases requiring localized intervention.

## Results

### Structure-guided engineering and optimization of photoactivatable split Cas13d

To construct a photoactivatable CRISPR/Cas13 system, we first selected RfxCas13d due to its high efficiency and specificity in RNA targeting. This RNA-guided RNA endonuclease has demonstrated robust activity in mammalian cells, offering a promising tool for precise transcriptome modulation. Notably, RfxCas13d’s compact size facilitates efficient delivery, and it does not require protospacer flanking sites (PFS), allowing for flexible guide RNA design.

**Fig. 1:**
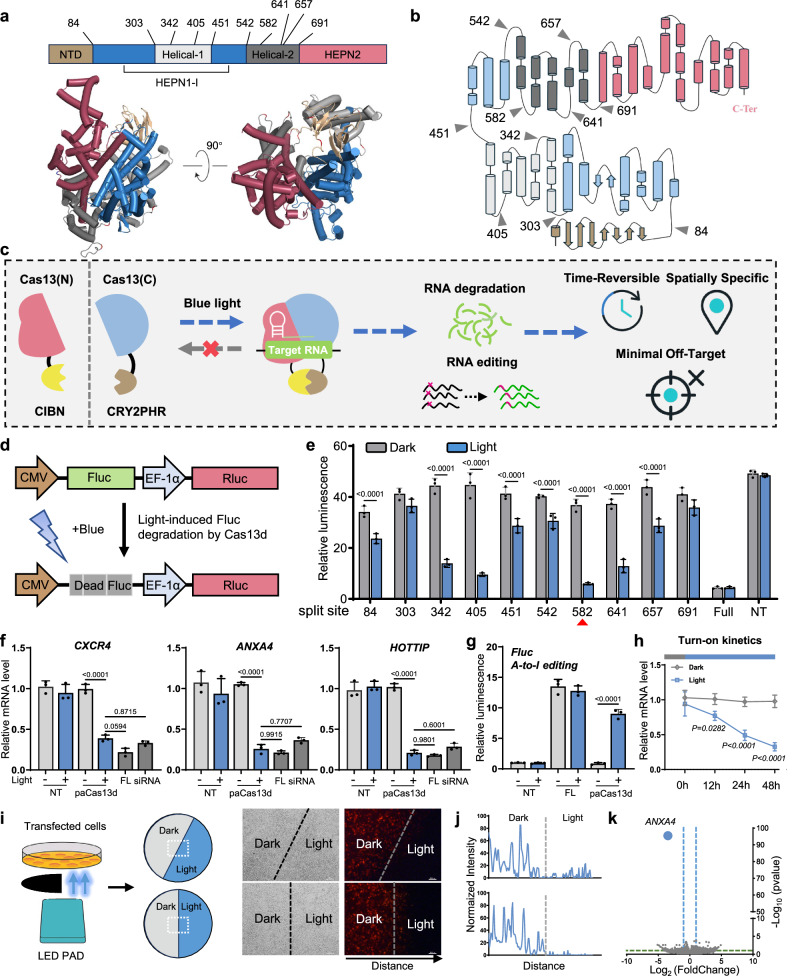
Structure-guided engineering and validation of paCas13d system. **a** AlphaFold-predicted three-dimensional structure of RfxCas13d showing the characteristic bi-lobed architecture. Two orthogonal views (90° rotation) highlight the spatial distribution of split sites across the protein architecture. **b** Schematic representation of the protein topology showing the relative positions of all ten split sites within the linear sequence and secondary structure elements. **c** Engineering strategy for photoactivatable Cas13d through CRY2PHR/CIBN-mediated split protein reconstitution. RfxCas13d is rationally divided into N-terminal and C-terminal fragments fused to CIBN (yellow) and CRY2PHR (brown), respectively. Upon blue light illumination (≈470 nm), CRY2PHR undergoes conformational changes promoting high-affinity heterodimerization with CIBN, enabling spatial approximation of split fragments and restoration of catalytic activity for targeted RNA degradation or editing. **d** Dual-luciferase reporter system for quantitative assessment of paCas13d activity. **e** Comprehensive functional screening of all ten split-site combinations under standardized blue light conditions (470 nm, 0.002 W/cm², 3 s on/60 s off cycling) in HEK293T cells. Data represent relative luminescence (Fluc/Rluc) comparing dark (gray bars) versus light-activated (blue bars) conditions. **f** Validation of endogenous RNA targeting specificity and efficiency. NT non-targeting control, FL full-length Cas13d. **g** Programmable RNA base editing capability through padCas13-ADAR2_DD_ fusion system. **h** Temporal dynamics and reversibility of paCas13d-mediated *CXCR4* knockdown. Time-course analysis reveals progressive mRNA depletion during continuous illumination. **i** Spatial precision assessment through half-field patterned illumination. Schematic (left) shows experimental setup with photomask covering half the culture dish. Confocal microscopy images (right) demonstrate spatially-restricted mCherry knockdown exclusively in illuminated regions, with sharp demarcation between light-exposed and protected areas. Scale bar = 100 μm. **j** Quantitative spatial analysis of mCherry fluorescence intensity. **k** Transcriptome-wide specificity analysis through RNA sequencing. Scatter plot comparing |log₂(FC)| and −log_10_(*P* value) values of all expressed genes between *ANXA4*-targeting paCas13d and non-targeting control conditions. For (**e–h**), *n* = 3 independent experiments. Data are presented as mean ± SD. *P* values were calculated by two-way ANOVA with multiple comparisons for (**e**, **h**), and one-way ANOVA with multiple comparisons for (**f**, **g**). *P* values in (**k**) were calculated using a negative binomial generalized linear model via the Wald test. Source data are provided as a Source Data file.

In the absence of an experimentally determined structure for RfxCas13d, we utilized AlphaFold Server (https://alphafoldserver.com/) to predict its full-length three-dimensional structure (Fig. 1a). The predicted template modeling (pTM) score was 0.94, suggesting that there was a high confidence in the predicted structure. We further mapped the functional domain architecture of RfxCas13d based on structural homology with EsCas13d^19^. This analysis identified the characteristic bi-lobed organization consisting of an N-terminal domain (NTD), two helical domains (Helical-1 and Helical-2), and dual HEPN (Higher Eukaryotes and Prokaryotes Nucleotide-binding) nuclease domains. Critically, we identified solvent-exposed flexible loops connecting these structured domains as prime candidates for protein splitting while preserving catalytic integrity. Through comprehensive structural analysis, we systematically evaluated ten strategically positioned split sites (84, 303, 342, 405, 451, 542, 582, 641, 657, and 691) located within inter-domain linker regions and surface-accessible loops (Fig. 1b). Each split site was selected based on three key criteria: (1) minimal disruption to secondary structure elements, (2) maximal solvent accessibility to enable optogenetic domain fusion, and (3) geometric compatibility for light-induced reconstitution.

Then we implemented the well-characterized CRY2PHR/CIBN blue light-inducible dimerization system to control split Cas13d reconstitution (Fig. 1c). The molecular mechanism underlying this optogenetic switch has been extensively established: the CRY2PHR photolyase homology region contains a flavin adenine dinucleotide (FAD) chromophore that undergoes photoreduction upon blue light absorption, triggering a conformational change from a closed, CIBN-inaccessible state to an open conformation that exposes the CIBN-binding interface and enables high-affinity heterodimer formation (K_D_ ~ 1 μM)^12,20^. Upon light withdrawal, the FAD chromophore spontaneously re-oxidizes in the dark, reverting CRY2PHR to its closed conformation and causing dimer dissociation. This reversible photocycle enables light-gated spatial approximation of split Cas13d fragments, restoring catalytic activity exclusively under illumination while maintaining inactive separation in darkness. To directly validate the functional operation of this light-dependent interaction, we performed nucleus-cytoplasm translocation assays that exploit competing nuclear localization and export signals to visualize heterodimerization dynamics. This design strategy, analogous to the inducible split-Cas9 system^11^, employs CIBN-GFP containing dual nuclear localization signals (NLS) at both termini to ensure strong nuclear accumulation, while CRY2PHR-mCherry contains a single nuclear export signal (NES) directing cytoplasmic retention (Supplementary Fig. 1a). In darkness, the two proteins remain spatially segregated with CIBN-GFP nuclear and CRY2PHR-mCherry cytoplasmic. Upon 470 nm illumination, CRY2PHR-mCherry rapidly accumulated in the nucleus within 1 min, demonstrating robust light-induced translocation (Supplementary Fig. 1b). This nuclear accumulation occurs because heterodimerization with CIBN creates a complex in which the nuclear import activity mediated by CIBN’s dual NLS predominates over the nuclear export activity conferred by CRY2PHR’s single NES, driving net nuclear translocation of the heterodimer. Critically, other wavelengths (365, 530, 635, 730 nm) outside the blue light absorption range elicited no response, demonstrating strict wavelength specificity consistent with the FAD chromophore absorption profile. Upon light cessation, nuclear CRY2PHR-mCherry gradually decreased over 30 min, returning to baseline cytoplasmic distribution and demonstrating complete reversibility (Supplementary Fig. 1b, c). This reversible nuclear clearance occurs through a two-step mechanism: the CRY2PHR/CIBN heterodimer first undergoes spontaneous dissociation in darkness, followed by NES-mediated active nuclear export of free CRY2PHR-mCherry to the cytoplasm, while CIBN-GFP remains nuclear due to its dual NLS. The observed kinetics are consistent with published CRY2PHR/CIBN dissociation parameters, confirming the dynamic and reversible nature of this optogenetic switch^12,20^. Dark controls showed negligible spontaneous translocation throughout the observation period. These functional validation experiments confirm that our paCas13d system operates through the well-established CRY2PHR/CIBN photocycle, providing wavelength-specific, and reversible optogenetic control over Cas13d activity.

To rigorously evaluate the RNA interference activity of our split Cas13d library, we developed a quantitative dual-luciferase reporter system in HEK293T cells that enables real-time monitoring of RNA degradation efficiency (Fig. 1d). This orthogonal reporter design expresses firefly luciferase (Fluc) and Renilla luciferase (Rluc) under independent CMV and EF-1α promoters, respectively, where Fluc serves as the paCas13d activity readout while Rluc provides robust internal normalization control. Comprehensive screening of all ten split site combinations under standardized blue light conditions (470 nm, 0.002 W/cm², 3 s on/60 s off cycling) revealed a striking heterogeneity in reconstitution efficiency (Fig. 1e). Eight split pairs (sites 84, 342, 405, 451, 542, 582, 641, and 657) demonstrated significant light-induced reporter downregulation, while others showed minimal or no light-dependent activity. Remarkably, the N582/C583 split site exhibited the most pronounced light-dependent activation and emerged as the optimal variant with superior dynamic range and stringent optogenetic control.

### Functional characterization and precision validation of the paCas13d system

To establish the translational potential of our paCas13d system, we validated its capacity to target endogenous mammalian transcripts with high specificity and efficiency. Transfection of HEK293T cells with optimized paCas13d (N582/C583) and crRNAs targeting therapeutically relevant genes, *CXCR4* (chemokine receptor), *ANXA4* (calcium-binding protein), and *HOTTIP* (oncogenic lncRNA), demonstrated robust light-dependent knockdown comparable to both full-length Cas13d and gold-standard siRNA controls (Fig. 1f). Crucially, the system maintained stringent optogenetic control with minimal dark-state activity across all targets tested.

To demonstrate the versatility and programmability of our platform, we engineered a catalytically inactive variant (padCas13) fused to the ADAR2 deaminase domain (ADAR2_DD_) for precision RNA base editing applications (Fig. 1g and Supplementary Fig. 2a). Using a dual-luciferase reporter harboring a premature UAG stop codon (W417X) that abolishes firefly activity, we demonstrated efficient light-dependent A-to-I editing that restores the tryptophan codon (UGG) and luminescence function. The padCas13-ADAR2_DD_ system achieved ~ten fold luciferase rescues exclusively under blue light illumination, establishing precise optogenetic control over RNA editing with single-nucleotide resolution.

To confirm that the photoactivatable system responds exclusively to blue light and not to NIR wavelengths used for upconversion activation, we performed critical control experiments. Exposure to NIR light (980 nm) in the absence of UCNPs failed to activate either paCas13d-mediated RNA degradation (Supplementary Fig. 2b) or padCas13-ADAR2_DD_-mediated base editing (Supplementary Fig. 2c). These results unequivocally demonstrate that the CRY2PHR/CIBN optogenetic switch exhibits no intrinsic photosensitivity to NIR wavelengths, confirming that functional activation observed with UCNPs+NIR treatment is entirely attributable to upconversion-mediated generation of blue light.

We next examined the kinetics of paCas13d‑mediated RNA degradation. Time-course analysis of paCas13d-mediated *CXCR4* knockdown revealed progressive mRNA depletion kinetics, with transcript levels decreasing to ~30% of baseline within 48 h of continuous illumination (Fig. 1h). Importantly, this light-induced knockdown exhibited complete reversibility, with mRNA levels returning to baseline within 36 h following light withdrawal (Supplementary Fig. 3), demonstrating the temporal precision and non-permanent nature of our optogenetic intervention.

To evaluate the spatial resolution of our paCas13d system, we performed half-field patterned illumination experiments using cells transfected with mCherry-targeting constructs (Fig. 1i). Confocal microscopy analysis revealed exquisite spatial control, with mCherry fluorescence selectively reduced only within illuminated regions while maintaining normal expression levels in masked areas. This sharp demarcation between light-exposed and protected regions (Fig. 1j) demonstrates the potential for anatomically precise therapeutic interventions with minimal effects on adjacent tissues.

Finally, to rigorously assess the specificity of paCas13d-mediated knockdown and exclude potential off-target effects, we performed comprehensive RNA sequencing analysis comparing cells transfected with *ANXA4*-targeting paCas13d versus controls (Fig. 1k). Transcriptome-wide analysis revealed high on-target specificity with *ANXA4* as the predominant differentially expressed gene, while the vast majority of transcripts remained unaffected. This high-fidelity targeting profile establishes paCas13d as a precision tool for RNA manipulation with minimal transcriptome perturbation.

### Preparation and characterization of UCNPs-PEI

**Fig. 2:**
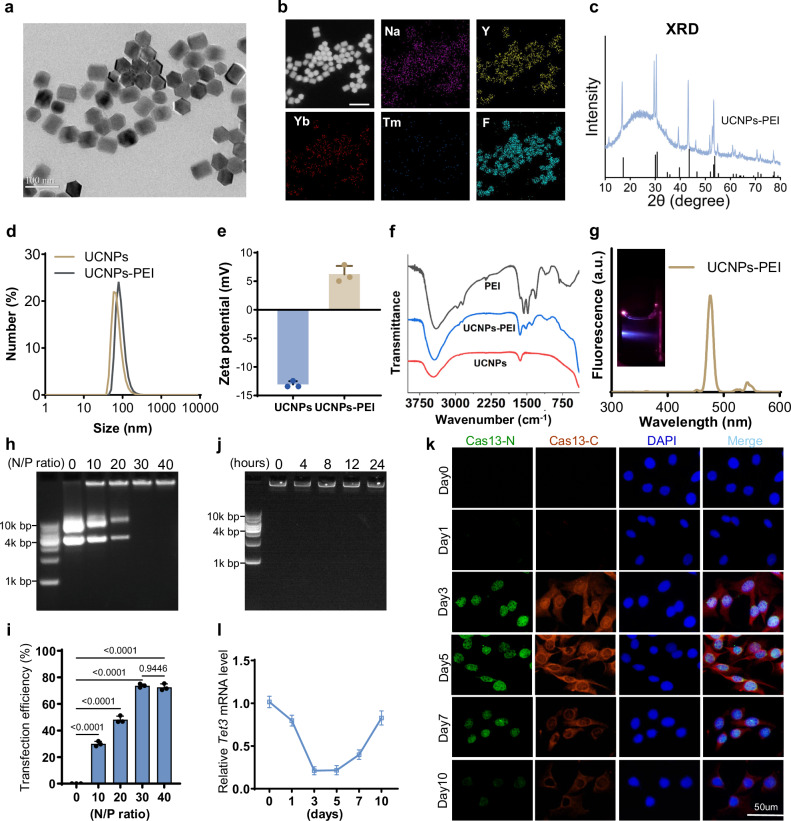
Characterization of UCNPs-PEI. **a** Transmission electron microscopy (TEM) analysis showing monodisperse and uniform UCNPs-PEI (scale bar = 100 nm). **b** Elemental mapping analysis of the UCNPs-PEI shown in (**a**), confirming uniform distribution of Na, Y, Yb, Tm, and F. **c** X-ray diffraction (XRD) pattern of UCNPs-PEI. **d** DLS analysis of the hydrodynamic size distribution of ligand-free UCNPs and UCNPs-PEI. **e** Zeta potential analysis of UCNPs and UCNPs-PEI. **f** Fourier-transform infrared spectroscopy (FTIR) spectra analysis of UCNPs and UCNPs-PEI. **g** Upconversion emission spectrum of UCNPs-PEI under 980 nm NIR excitation. Inset: photograph of UCNPs-PEI solution under 980 nm NIR illumination. **h** Agarose gel electrophoresis of UCNPs-PEI@DNA complexes at different N/P ratios (0, 10, 20, 30, 40). **i** Transfection efficiency assay of UCNPs-PEI@GFP complexes in HEK293T cells at various N/P ratios. **j** Serum stability assay of UCNPs-PEI@paCas13d complexes over 24 h. **k** Fluorescence microscopy of split-Cas13 expression (Cas13-N in green, Cas13-C in red, DAPI in blue) at the indicated time points post-transfection in MLO-Y4 cells, scale bar = 50 μm. **l** Quantitative RT-PCR analysis of *Tet3* mRNA expression following UCNPs-PEI@paCas13d mediated knockdown over 10 days. For (**e**, **i**, and **l**), *n* = 3 independent experiments. Data are presented as mean ± SD. *P* values in (**i**) were calculated by one-way ANOVA with multiple comparisons. Source data are provided as a Source Data file.

UCNPs were introduced to harness the advantages of NIR light for deep tissue optogenetic applications. UCNPs can convert tissue-penetrating NIR light into visible light, facilitating non-invasive activation of paCas13d in vivo. First, the UCNPs-PEI were prepared and exhibited good monodispersity and uniform size (Fig. 2a). Element mapping analysis showed that different elements (Na, Y, Yb, F and Tm) were uniformly distributed in the UCNPs-PEI (Fig. 2b). The X-ray diffraction (XRD) pattern of the UCNPs-PEI after shell growth and subsequent ligand modification matches well with the standard hexagonal β-NaYF₄ phase with no detectable impurity peaks, indicating that the crystalline host lattice is preserved (Fig. 2c). Dynamic light scattering (DLS) measurements showed that ligand-free UCNPs had a narrow hydrodynamic size distribution (~77.5 nm), which increased to ~88.7 nm after PEI functionalization (Fig. 2d). The successful PEI coating was further supported by the positive zeta potential shift (Fig. 2e) and pronounced -CH₂- vibrations (1450–1480 cm^−1^) indicated by the FTIR analysis (Fig. 2f). Under 980 nm excitation, both ligand-free UCNPs (before PEI modification) and UCNPs-PEI (after PEI modification) exhibited a dominant blue emission band centered at ~470 nm (Fig. 2g and Supplementary Fig. 4a). Direct comparison under identical acquisition settings shows that PEI functionalization preserves the dominant ~470 nm blue emission, although minor high-energy features in the UV/∼450 nm region are relatively attenuated. The blue emission output was further quantified at the same excitation power densities, showing comparable blue light emission between ligand-free UCNPs and UCNPs-PEI (Supplementary Fig. 4b). In addition, thermogravimetric analysis indicated that PEI constituted approximately 3.16% of the UCNPs-PEI mass (Supplementary Fig. 4c).

Having established the physicochemical properties of UCNPs-PEI, we systematically optimized the formulation parameters for efficient plasmid DNA delivery. The nitrogen-to-phosphate (N/P) ratio, representing the molar ratio of positively charged amine groups on PEI to negatively charged phosphate groups on DNA, is a critical determinant of complexation efficiency and transfection performance. Gel retardation assay showed complete DNA binding at N/P ≥ 30 (Fig. 2h), while transfection efficiency reached maximum at N/P = 30 with no further gains at higher ratios (Fig. 2i). Cell viability remained >95% across all tested conditions (Supplementary Fig. 4d). Based on these results, N/P = 30 was selected as the optimal formulation, achieving maximal gene delivery without compromising cell viability.

To assess stability under physiological conditions, we first characterized the complexation and colloidal properties of UCNPs-PEI@paCas13d. DLS measurements showed that plasmid loading increased the hydrodynamic diameter from 88.7 nm (UCNPs-PEI) to 232 nm (UCNPs-PEI@paCas13d), confirming successful DNA complexation (Supplementary Fig. 4e). Longitudinal assessment over 5 days revealed that both particle size and polydispersity index remained stable for DNA-loaded complexes (Supplementary Fig. 4e, f), indicating robust colloidal stability. We next challenged these complexes under physiologically relevant stress conditions. Gel electrophoresis confirmed that complexes remained largely intact after incubation in 10% fetal bovine serum (Fig. 2j) and resisted dissociation in 500 mM NaCl (Supplementary Fig. 4g), demonstrating resistance to both protein competition and ionic screening. These comprehensive stability assessments confirm the suitability of UCNPs-PEI@paCas13d for therapeutic applications. To assess functional persistence, we transfected MLO-Y4 cells with UCNPs-PEI@paCas13d and monitored Cas13d expression and target gene suppression over time. Immunofluorescence analysis showed sustained expression of both Cas13d-N and Cas13d-C fragments for up to 7 days post-transfection, with peak expression at Days 3–5 (Fig. 2k). Correspondingly, *Tet3* mRNA levels remained significantly suppressed through Day 7 but began recovering toward baseline by Day 10 (Fig. 2l), defining a functional window of approximately one week and providing the rationale for weekly dosing in subsequent in vivo studies. Moreover, cell viability remained above 95% at UCNPs-PEI concentrations up to 1 mg/mL (Supplementary Fig. 4h). This acceptable biocompatibility is attributed to the low PEI content in the formulation (Supplementary Fig. 4c). Parallel testing with equivalent concentrations of free PEI showed no significant cytotoxicity (Supplementary Fig. 4i). These comprehensive characterization studies establish UCNPs-PEI as a stable and biocompatible platform for paCas13d delivery, providing the foundation for subsequent functional evaluation.

### UCNPs-PEI mediated paCas13d for RNA degradation and A-to-I editing

**Fig. 3:**
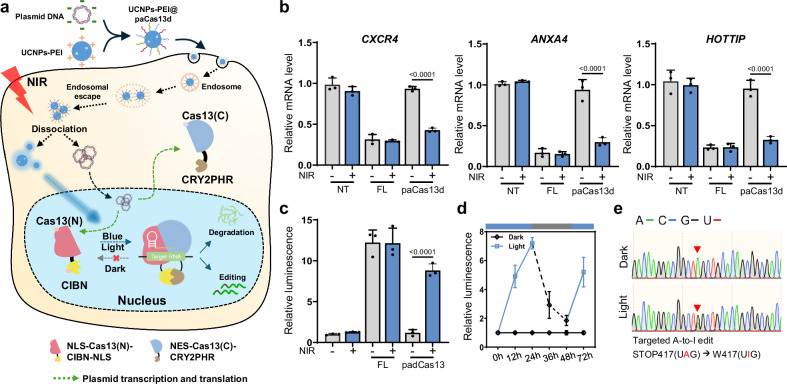
UCNPs-PEI mediated paCas13d for NIR-activated RNA manipulation. **a** Schematic illustration of UCNPs-PEI delivery system for NIR-activated paCas13d. Split Cas13d fragments are delivered via PEI-functionalized upconversion nanoparticles. Upon NIR illumination (980 nm), UCNPs convert NIR to blue light (470 nm), triggering CRY2PHR/CIBN dimerization and functional reconstitution of paCas13d for targeted RNA degradation or base editing. **b** NIR-activated RNA knockdown efficiency of endogenous transcripts *CXCR4*, *ANXA4*, and *HOTTIP*. Comparison between non-targeting control (NT), full-length Cas13d (FL), and paCas13d under dark (−) and NIR illumination (+) conditions. **c** A-to-I RNA base editing activity assessed through dual-luciferase reporter rescue assay. padCas13-ADAR2_DD_ system restores firefly luciferase activity by converting premature UAG stop codon to functional UGG codon exclusively under NIR illumination. **d** Temporal dynamics and reversibility of NIR-activated RNA base editing. Time-course analysis demonstrates progressive luciferase activity increase during NIR exposure followed by rapid decline upon light withdrawal, confirming temporal precision and reversible control. **e** Molecular confirmation of A-to-I editing through Sanger sequencing. Chromatograms show A-to-G conversion (red arrowheads) at position 417 in NIR-treated samples, converting UAG stop codon to UIG (read as UGG), while dark controls maintain original adenosine residue. For (**b–d**), *n* = 3 independent experiments. Data are presented as mean ± SD. *P* values in (**b**, **c**) were calculated by one-way ANOVA with multiple comparisons. Source data are provided as a Source Data file.

Having established the paCas13d system and characterized the UCNPs-PEI nanoparticles, we next investigated whether UCNPs-PEI could efficiently deliver the paCas13d system and enable NIR light-triggered RNA manipulation in mammalian cells. The UCNPs-PEI@paCas13d essentially functions as a typical PEI/DNA polyplex nanocarrier, and the intracellular delivery cascade of PEI-based gene vectors has been extensively described in the literature^21,22^. In brief, the cationic PEI surface promotes adsorption to the cell membrane and initiates energy-dependent endocytosis (often dominated by clathrin-mediated uptake, with potential contributions from other endocytic routes depending on cell type and formulation). Following internalization, PEI-containing polyplexes can promote endosomal escape, which is often discussed in the context of PEI’s buffering capacity (the “proton-sponge” framework), allowing the complexes to access the cytosol. Subsequently, polyplex decondensation releases plasmid DNA, enabling downstream nuclear entry and gene expression (Fig. 3a). To leverage this platform for optogenetic RNA manipulation, we designed a comprehensive experimental approach to evaluate both RNA degradation and A-to-I editing capabilities mediated by UCNPs-PEI.

To assess the RNA knockdown efficiency of UCNPs-PEI delivered paCas13d under NIR illumination, we transfected HEK293T cells with UCNPs-PEI complexed with paCas13d components and crRNAs targeting endogenous transcripts *CXCR4*, *ANXA4*, and the lncRNA *HOTTIP*. Following transfection, cells were exposed to 980 nm NIR light (1.3 W/cm², 3 s on/60 s off cycles) for 48 h. Quantitative RT-PCR analysis revealed that NIR-activated paCas13d achieved robust knockdown of all three targets (Fig. 3b). Importantly, cells maintained in the dark showed minimal knockdown activity, demonstrating tight optogenetic control even with NIR-mediated activation through UCNPs.

We further expanded the capability of our UCNPs-PEI delivery system to enable programmable RNA base editing. Using the padCas13 editor system, we evaluated A-to-I editing activity through a dual-luciferase reporter containing a premature stop codon (W417X). Successful A-to-I editing converts the UAG stop codon to UIG (read as UGG), thereby restoring firefly luciferase expression. NIR illumination of cells transfected with UCNPs-PEI@padCas13 resulted in significant restoration of luciferase activity (Fig. 3c). This efficiency was comparable to that of direct blue light activation (Supplementary Fig. 5). To understand why NIR-mediated activation achieved efficiency comparable to direct blue light, we quantified the blue light emission from NIR-excited UCNPs. Spectral analysis revealed that UCNPs under 1 W/cm² NIR excitation (980 nm) emit approximately 0.04 W/cm² of blue light (~470 nm), which exceeds the direct blue light intensity used in our experiments (0.002 W/cm², Supplementary Fig. 4b). Both illumination conditions thus deliver sufficient blue light doses to saturate optogenetic activation, explaining the equivalent editing efficiencies, while NIR offers the critical advantage of deep tissue penetration.

To characterize the temporal dynamics and reversibility of NIR-activated RNA editing, we performed time-course experiments monitoring luciferase activity over 72 h. The relative luminescence progressively increased with continuous NIR exposure. Remarkably, upon removal of NIR illumination, the luminescence immediately decreased, returning to near-baseline levels within 24 h (Fig. 3d). This reversible control demonstrates that the UCNPs-PEI mediated system maintains the same temporal precision as the original blue light-activated system. To definitively confirm A-to-I editing at the molecular level, we performed Sanger sequencing of the target site in the firefly luciferase reporter. Sequencing chromatograms clearly showed the expected A-to-G conversion (corresponding to A-to-I editing at the RNA level) at position 417, converting the UAG stop codon to UIG in NIR-treated samples, while dark controls maintained the original adenosine residue (Fig. 3e).

Collectively, these results establish UCNPs-PEI@paCas13d as an efficient platform for programmable RNA manipulation in mammalian cells, enabling both targeted degradation and precision base editing under NIR light control. Having validated the specificity, efficiency, and temporal precision of this photoactivatable system, we next sought to evaluate its therapeutic potential in SAON disease, where spatially confined gene regulation could provide therapeutic benefit.

### UCNPs-PEI@paCas13d mediated knockdown of TET3 for prevention of steroid-associated osteocyte apoptosis

**Fig. 4:**
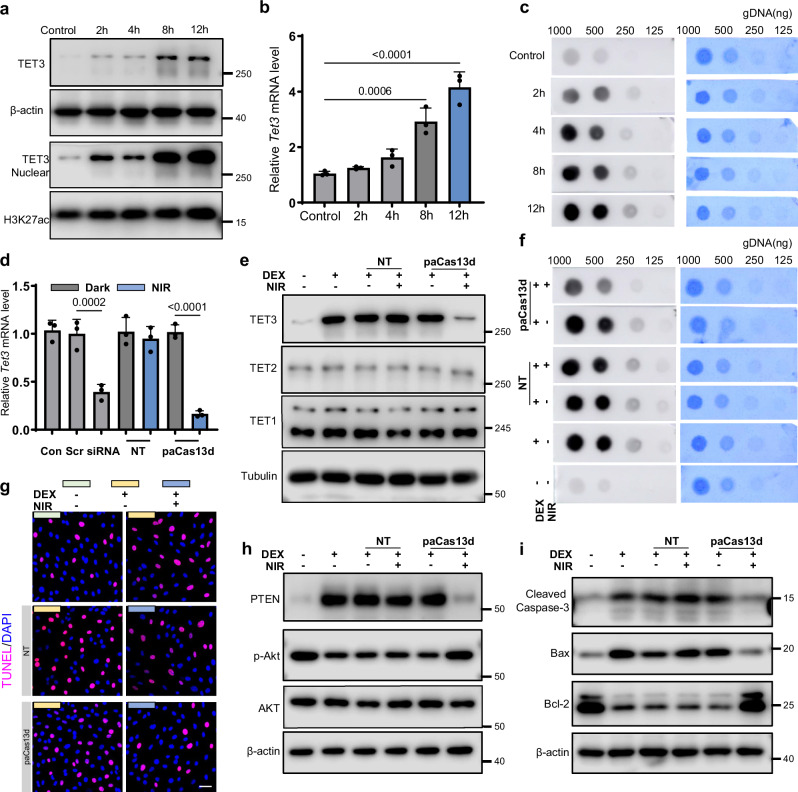
UCNPs-PEI@paCas13d mediated knockdown of TET3 for prevention of steroid-associated osteocyte apoptosis. **a** Time-course analysis of TET3 protein expression in MLO-Y4 osteocyte-like cells following DEX treatment. Western blot analysis shows total TET3 protein levels (upper panel) and nuclear TET3 localization (lower panel) at indicated time points. β-actin and H3K27ac serve as loading controls for total and nuclear fractions, respectively. **b** Quantitative RT-PCR analysis of *Tet3* mRNA expression during DEX treatment time course. **c** Dot-blot analysis of global 5-hydroxymethylcytosine (5hmC) levels in genomic DNA during DEX treatment. Serial dilutions of genomic DNA (1000, 500, 250, and 125 ng) were analyzed at different time points. Left panels show 5hmC detection; right panels show methylene blue staining for loading control. **d** TET3 knockdown efficiency by UCNPs-PEI@paCas13d system. Quantitative RT-PCR analysis comparing scramble siRNA (Scr), siRNA targeting TET3, non-targeting paCas13d (NT), and paCas13d targeting TET3 under dark and NIR illumination conditions. **e** Western blot analysis confirming specific TET3 protein knockdown without affecting TET1 and TET2 expression levels. **f** Functional validation of TET3 knockdown through 5hmC level assessment. Dot-blot analysis demonstrates reduced global 5hmC levels following NIR-activated paCas13d treatment, confirming disruption of TET3-mediated DNA demethylation activity. **g** In situ TUNEL staining for apoptosis detection in MLO-Y4 cells. Red fluorescence indicates apoptotic cells; blue (DAPI) shows total cell nuclei. Images show representative fields from different treatment groups. Scale bar = 50 μm. **h** Western blot analysis of PTEN-Akt survival signaling pathway. **i** Western blot analysis of apoptosis-related proteins. Expression levels of pro-apoptotic markers (cleaved Caspase-3, Bax) and anti-apoptotic marker (Bcl-2) were assessed. For (**b** and **d**), *n* = 3 independent experiments. Data are presented as mean ± SD. *P* values in (**b**, **d**) were calculated by one-way ANOVA with multiple comparisons. Source data are provided as a Source Data file.

Given the essential role of the TET3–5hmC signal in the pathogenesis of steroid-associated osteonecrosis (SAON), we first examined the dynamic expression of TET3 in MLO-Y4 osteocyte-like cells following dexamethasone (DEX) treatment. Both total and nuclear TET3 protein levels were markedly increased in a time-dependent manner, as confirmed by Western blotting and subcellular fractionation (Fig. 4a). Consistently, qRT-PCR revealed significant upregulation of *Tet3* mRNA, and dot-blot assays demonstrated a parallel increase in global 5-hydroxymethylcytosine (5hmC) levels, indicating enhanced TET3 enzymatic activity (Fig. 4b, c). These findings highlight TET3-mediated epigenetic reprogramming as a key driver of DEX-induced osteocyte apoptosis and a promising therapeutic target for SAON.

To determine whether targeted silencing of TET3 could mitigate this pathological response, we employed our UCNPs-PEI platform to introduce the paCas13d system into MLO-Y4 cells. Upon exposure to 980 nm NIR illumination (1.3 W/cm², 3 s on/60 s off cycles for 48 h), TET3 transcript levels were robustly reduced by > 80%, outperforming conventional small interfering RNAs (siRNAs) (Fig. 4d). Importantly, this targeted knockdown exhibited excellent specificity, as the expression levels of other TET family members (TET1 and TET2) remained unchanged across all treatment conditions (Supplementary Fig. 6), confirming selective targeting of TET3 without off-target effects on related epigenetic regulators. Western blot analysis further confirmed efficient suppression of TET3 protein expression, while the expression levels of other TET family members remained unaffected, indicating high target specificity (Fig. 4e). Importantly, dot-blot analysis revealed a significant reduction in global 5hmC levels following paCas13d activation (Fig. 4f), corroborating functional silencing of TET3 activity.

Given that osteocyte apoptosis as a key pathological feature of SAON, we investigated the protective effect of TET3 silencing on DEX-induced cell death. TUNEL staining revealed a significant reduction in DEX-induced apoptosis in NIR-activated paCas13d-treated cells, whereas non-targeting controls failed to show protective effects (Fig. 4g and Supplementary Fig. 7a). In parallel, CCK-8 assays showed that NIR-activated paCas13d markedly restored MLO-Y4 cell viability (Supplementary Fig. 7b). Consistent with these observations, downstream analysis of the PTEN–Akt signaling pathway demonstrated suppression of PTEN expression and a concomitant increase in phosphorylated Akt (p-Akt) levels (Fig. 4h), indicating activation of pro-survival signaling. Furthermore, we observed downregulation of the pro-apoptotic proteins Bax and cleaved Caspase-3, along with upregulation of the anti-apoptotic marker Bcl-2 (Fig. 4i), supporting a shift toward cell survival upon TET3 suppression. Collectively, these results demonstrate that UCNPs-PEI-mediated delivery of the NIR-activated paCas13d system enables precise, efficient, and selective knockdown of TET3 in osteocytes. This silencing mitigates DEX-induced epigenetic reprogramming, reactivates survival pathways, and protects against apoptosis - highlighting the therapeutic potential of the UCNPs-PEI@paCas13d system for the prevention of SAON.

### Pharmacokinetics and biodistribution of UCNPs-PEI@paCas13d in vivo

**Fig. 5:**
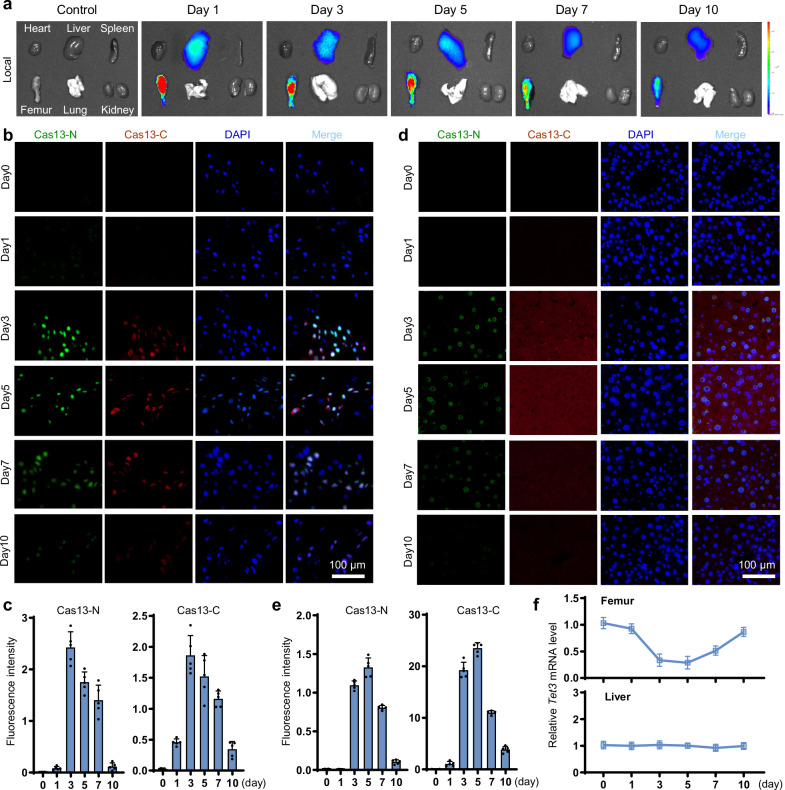
Pharmacokinetics and biodistribution of UCNPs-PEI@paCas13d following intrafemoral injection. **a** Representative in vivo fluorescence imaging showing biodistribution of UCNPs-PEI@paCas13d at Days 1, 3, 5, 7, and 10 following intrafemoral injection. **b** Time-course immunofluorescence imaging of split-Cas13d expression in femoral tissue. Cas13d-N (green), Cas13d-C (red), and nuclei (DAPI, blue) were detected at Days 0, 1, 3, 5, 7, and 10 post-injections. Scale bar = 100 μm. **c** Quantitative analysis of Cas13d-N and Cas13d-C fluorescence intensity in femoral tissue over time. **d** Immunofluorescence imaging of Cas13d expression in hepatic tissue at the same time points. Scale bar = 100 μm. **e** Quantitative analysis of Cas13d-N and Cas13d-C fluorescence intensity in liver tissue. **f** Quantitative RT-PCR analysis of *Tet3* mRNA levels in femur and liver tissues over 10 days. For (**c**, **e**, and **f**), *n* = 5 animals per group. Data are presented as mean ± SD. Source data are provided as a Source Data file.

For safe and effective clinical translation, we comprehensively assessed the pharmacokinetics and biodistribution of UCNPs-PEI@paCas13d in vivo, to establish a rational dosing regimen and evaluate potential off-target effects. Following intrafemoral injection, longitudinal in vivo fluorescence imaging revealed that UCNPs-PEI remained predominantly localized to the injected femur, with peak fluorescence signals observed at Days 1–5 and gradual decline over 10 days (Fig. 5a). Notably, progressive hepatic accumulation was also detected, indicating hepatobiliary clearance as the primary metabolic pathway. To correlate nanoparticle distribution with functional gene delivery, we performed immunofluorescence analysis of femoral and hepatic tissues at multiple time points. In the femur, robust expression of both Cas13d-N and Cas13d-C fragments was observed at Days 3–7, with significant reduction by Day 10 (Fig. 5b, c), consistent with nanoparticle clearance and plasmid degradation kinetics. Most importantly, functional assessment revealed that femoral *Tet3* mRNA levels remained significantly suppressed through Day 7 but returned toward baseline by Day 10 (Fig. 5f), defining a therapeutic window of approximately 7 days. Critically, despite detectable Cas13d-N and Cas13d-C expression in hepatic tissue (Fig. 5d, e), hepatic *Tet3* mRNA levels remained unchanged throughout the 10-day observation period (Fig. 5f), demonstrating that paCas13d remains functionally inactive in non-illuminated tissues even when successfully delivered. Based on this pharmacokinetic profile, we implemented a weekly intrafemoral injection regimen for the subsequent SAON treatment protocol.

To directly compare delivery routes and further validate the necessity of local administration, we performed parallel biodistribution analysis following intravenous injection of UCNPs-PEI@paCas13d. As expected for nanoparticles in this size range (~200 nm), systemic administration resulted in rapid and predominant hepatic sequestration via reticuloendothelial system uptake, with peak signals at Days 1–5 and gradual clearance by Day 10 (Supplementary Fig. 8a). In stark contrast to intrafemoral injection, femoral accumulation following intravenous delivery was minimal, with correspondingly negligible Cas13d expression in femoral tissue (Supplementary Fig. 8b, c), rendering systemic delivery fundamentally inefficient for bone-targeted applications. Immunofluorescence analysis revealed transient hepatic Cas13d expression at Days 3–7 following intravenous administration (Supplementary Fig. 8d, e), yet hepatic *Tet3* mRNA levels remained unchanged (Supplementary Fig. 8f), indicating negligible functional gene silencing despite substantial nanoparticle accumulation. This critical observation demonstrates that the requirement for NIR light activation provides spatial confinement of therapeutic activity, even when nanoparticles distribute systemically, gene editing occurs exclusively in illuminated tissues. These pharmacokinetic studies established the rationale for weekly intrafemoral dosing and confirmed light-gated spatial specificity. We therefore proceeded to evaluate therapeutic efficacy in a murine model of SAON.

### UCNPs-PEI@paCas13d mediated TET3 knockdown prevents SAON in vivo

To evaluate the therapeutic efficacy of UCNPs-PEI@paCas13d system in vivo, we first assessed its spatial specificity and tissue-targeting capability. Using an A-to-I editing firefly luciferase reporter system, we performed intrafemoral injection of UCNPs-PEI@padCas13d in mice (Supplementary Fig. 9a). Following NIR irradiation (980 nm, 1.3 W/cm², 3 s on/60 s off cycles), in vivo bioluminescence imaging revealed robust luciferase signal specifically localized to the injected femur, with no detectable signal in other organs or tissues (Supplementary Fig. 9b). This demonstrates the excellent spatial control and tissue-specific activation of our system, minimizing potential off-target effects on other organs.

**Fig. 6:**
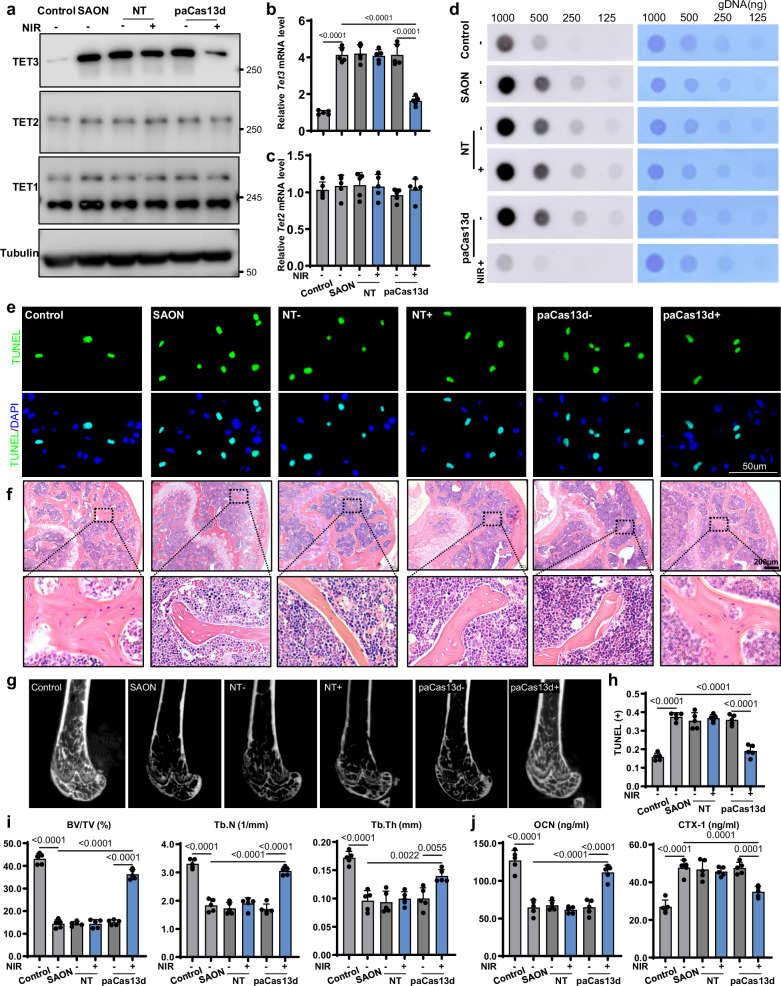
UCNPs-PEI@paCas13d mediated TET3 knockdown prevents SAON in vivo. **a** Western blot analysis of TET family proteins in femoral tissue from different treatment groups. SAON steroid-associated osteonecrosis, NT non-targeting control; paCas13d, TET3-targeting paCas13d; NIR (−/+), without/with near-infrared illumination. **b**, **c** Quantitative RT-PCR analysis of TET3 and TET2 mRNA expression levels in femoral tissue. **d** Dot-blot analysis of global 5hmC levels in genomic DNA at different contents (1000, 500, 250, 125 ng). Left panel shows chemiluminescent detection; right panel shows methylene blue staining for loading control. **e** In situ TUNEL staining for osteocyte apoptosis detection in trabecular bone. Green fluorescence indicates apoptotic cells; blue (DAPI) shows total cell nuclei. Scale bar = 50 μm. **f** Hematoxylin and eosin (H&E) staining of femur trabecular bone. Upper panels show low magnification overview; lower panels show high magnification of trabecular microarchitecture within boxed regions. Scale bars = 200 μm. **g** Representative micro-CT 3D reconstruction images of femur trabecular bone showing preservation of bone microarchitecture following NIR-activated paCas13d treatment. **h** Quantitative analysis of TUNEL-positive osteocytes. **i** Micro-CT quantitative analysis of trabecular bone parameters: bone volume fraction (BV/TV), trabecular number (Tb.N), and trabecular thickness (Tb.Th). **j** Serum bone turnover markers analysis. Osteocalcin (OCN) indicates bone formation activity; CTX-1 reflects bone resorption. For (**b**, **c** and **h–j**), *n* = 5 animals per group. Data are presented as mean ± SD. *P* values were calculated by one-way ANOVA with multiple comparisons. Source data are provided as a Source Data file.

Having established the spatial precision of our approach, we next investigated its therapeutic potential in a murine model of SAON. The SAON model was established through continuous oral administration of dexamethasone via drinking water over a 90-day period, as previously described^23^. UCNPs-PEI@paCas13d targeting TET3 or non-targeting control was delivered via intrafemoral injection, with the distal femur selected as the region of interest. NIR-activated paCas13d achieved highly specific and efficient TET3 knockdown in vivo. Western blot analysis of femoral tissue demonstrated substantial reduction of TET3 protein expression only in the TET3-targeted group with NIR activation, while TET1 and TET2 levels remained unchanged (Fig. 6a). This specificity was corroborated by RT-qPCR analysis, which showed approximately 60% reduction in TET3 mRNA levels (Fig. 6b), with no significant changes in TET1 or TET2 expression (Fig. 6c and Supplementary Fig. 10). Functionally, dot-blot analysis confirmed that TET3 knockdown led to a corresponding decrease in genomic 5hmC levels, validating the disruption of TET3-mediated epigenetic modifications (Fig. 6d).

The therapeutic benefit of TET3 knockdown was evident in preventing glucocorticoid-induced osteocyte apoptosis, a hallmark of SAON pathogenesis. TUNEL staining revealed that NIR-activated paCas13d treatment dramatically reduced osteocyte apoptosis in the TET3-targeted treatment group, approaching levels observed in healthy controls (Fig. 6e and h). Neither the non-targeting control nor the TET3-targeted group without NIR activation showed significant protective effects, confirming the requirement for both specific targeting and light activation. Histopathological examination through H&E staining further substantiated the protective effects. The SAON group exhibited severe trabecular deterioration characterized by numerous empty lacunae, disrupted trabecular architecture, and reduced trabecular number. In contrast, the TET3-targeted NIR treatment group showed well-preserved trabecular structure with significantly fewer empty lacunae and maintained trabecular integrity (Fig. 6f). Micro-CT analysis of the distal femur provided quantitative assessment of bone microarchitecture preservation. Three-dimensional reconstruction revealed marked trabecular loss in the SAON group, while the TET3-targeted paCas13d treatment group maintained trabecular connectivity comparable to healthy controls (Fig. 6g). Quantitative analysis demonstrated significant improvements in all microstructural parameters: bone volume fraction (BV/TV), trabecular number (Tb.N), and trabecular thickness (Tb.Th) were all significantly increased in the TET3-targeted NIR treatment group compared to the SAON group (Fig. 6i). Additionally, trabecular separation (Tb.Sp) was significantly reduced, indicating enhanced trabecular connectivity and improved bone microarchitecture (Supplementary Fig. 11).

Serum analysis of bone turnover markers revealed favorable metabolic changes following treatment. Serum osteocalcin (OCN), a marker of bone formation activity, was significantly increased in the TET3-targeted treatment group. Conversely, CTX-1, a marker of bone resorption, was significantly decreased, indicating a shift toward bone anabolism and reduced bone degradation (Fig. 6j). Importantly, comprehensive safety evaluation through blood routine examination and liver/kidney function tests showed no significant differences between treatment and control groups, confirming the excellent biocompatibility and absence of systemic toxicity of the UCNPs-PEI@paCas13d system (Supplementary Figs. 12 and 13).

To further establish the necessity of UCNPs-mediated NIR activation and benchmark therapeutic efficacy in vivo, we evaluated two critical controls in the SAON model. We compared siRNA-mediated *Tet3* knockdown against our photoactivatable system and tested whether direct blue light could activate UCNPs-PEI@paCas13d in deep bone tissue without upconversion. Both controls revealed fundamental limitations that our photoactivatable nanoparticle system overcomes. The siRNA control group, administered via intrafemoral injection, achieved comparable therapeutic efficacy in the target femur, demonstrating significant *Tet3* suppression (Supplementary Fig. 14a), preservation of trabecular bone microarchitecture (Supplementary Fig. 14b–d), and reduction in osteocyte apoptosis (Supplementary Fig. 14e, f). However, despite local administration, siRNA exhibited systemic redistribution to the liver, as evidenced by biodistribution imaging (Supplementary Fig. 14g). Critically, this hepatic accumulation resulted in unintended off-target *Tet3* suppression (Supplementary Fig. 14h) and significant hepatotoxicity, with elevated serum transaminases (Supplementary Fig. 14i). In stark contrast, the UCNPs-PEI@paCas13d+NIR group, despite similar hepatic nanoparticle accumulation, showed no hepatic *Tet3* suppression and maintained normal liver function (Supplementary Fig. 13), demonstrating that light-gated spatial control prevents off-target gene editing in non-illuminated tissues.

**Fig. 7:**
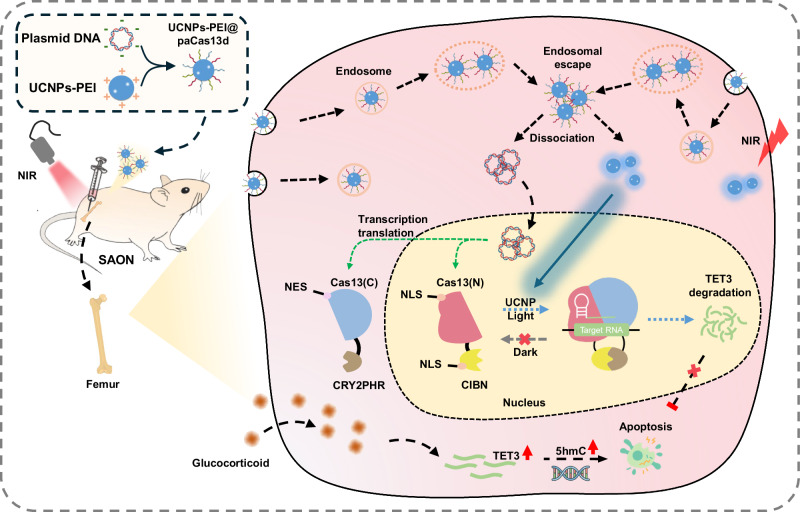
Schematic illustration of the UCNPs-PEI@paCas13d system for preventing SAON. Comprehensive overview of the NIR-activated paCas13 system for localized prevention of SAON. UCNPs-PEI nanoparticles serve as gene delivery vectors and photon transducers, facilitating efficient transfection of split paCas13d components into target cells via intrafemoral injection to bone tissue. Upon NIR illumination, UCNPs convert NIR to blue light, triggering CRY2PHR/CIBN heterodimerization and functional reconstitution of Cas13d. The activated paCas13d specifically degrades TET3 mRNA, preventing glucocorticoid-induced TET3 upregulation and subsequent epigenetic reprogramming that leads to osteocyte apoptosis. This spatially-restricted intervention protects bone tissue from steroid-induced damage while preserving systemic glucocorticoid therapeutic effects. The modular design enables precise temporal and spatial control of RNA therapeutic intervention through non-invasive external light activation.

The direct blue light control group, where UCNPs-PEI@paCas13d was delivered via intrafemoral injection followed by transcutaneous blue light illumination (470 nm, 0.002 W/cm²) instead of NIR, revealed the absolute necessity of upconversion-mediated photoactivation for deep tissue applications. Despite successful nanoparticle and paCas13d delivery to the femur, direct blue light activation failed to achieve any therapeutic benefit. Femoral *Tet3* expression remained elevated at levels comparable to untreated SAON controls (Supplementary Fig. 14a), trabecular bone parameters showed no improvement (Supplementary Fig. 14b–d), and osteocyte apoptosis remained unabated (Supplementary Fig. 14e, f). This complete absence of therapeutic effect demonstrates that external blue light cannot penetrate sufficiently through overlying skin, subcutaneous tissue, and muscle layers (~3–5 mm total depth) to reach the bone tissues and activate the optogenetic switch. Only through NIR excitation of UCNPs can blue light be generated locally within the deep tissue compartment, enabling effective photoactivation where direct external blue light is physically incapable of reaching. Collectively, these results demonstrate that the NIR-activated UCNPs-PEI@paCas13d system enables precise, spatially-controlled knockdown of TET3 in bone tissue, effectively preventing steroid-induced osteocyte apoptosis and preserving bone microarchitecture (Fig. 7). The combination of high therapeutic efficacy, spatial specificity, and excellent safety profile positions this optogenetic approach as a promising strategy for preventing SAON.

## Discussion

The development of our paCas13d system represents a significant advancement in the field of precision RNA therapeutics, addressing fundamental challenges that have limited the clinical translation of CRISPR-based technologies^24,25^. By integrating structural biology insights, optogenetic engineering, and nanomaterial science, we have created a platform that enables non-invasive, spatiotemporally controlled RNA modulation in deep tissues—a capability that has remained elusive despite intense research efforts.

Our systematic approach to engineering split Cas13d highlights the importance of structure-guided design in developing controllable CRISPR systems. The identification of the N582/C583 split site through comprehensive screening demonstrates that not all protein division points are functionally equivalent^26^. The success of this particular configuration likely reflects its location within a flexible loop region that permits reconstitution without disrupting critical catalytic residues or RNA-binding interfaces. Notably, the dynamic range achieved with our optimized split pair exceeds that reported for other split CRISPR systems, suggesting that Cas13d may be particularly amenable to optogenetic control. This finding has important implications for the broader field, as it suggests that systematic evaluation of split sites, rather than relying on homology-based predictions, may be essential for optimizing split protein systems.

The integration of upconversion nanotechnology with optogenetic control represents a critical innovation that addresses the fundamental limitation of light penetration in biological tissues^27,28^. Our UCNPs-PEI system serves dual functions as both a photon transducer and a gene delivery vehicle, streamlining the therapeutic platform. The choice of NaYF₄:Yb/Tm as the upconversion matrix was deliberate, as this composition provides optimal 980 to 470 nm conversion efficiency while maintaining biocompatibility. The PEI functionalization not only enables efficient cellular uptake and endosomal escape but also provides a versatile platform for further modifications, such as targeting ligands or stealth coatings for systemic delivery.

The therapeutic efficacy demonstrated in our SAON model provides compelling evidence for the clinical potential of spatially-restricted RNA modulation^29^. The specific targeting of TET3 represents a rational therapeutic strategy based on emerging understanding of SAON pathogenesis. By preventing TET3-mediated epigenetic reprogramming, we effectively maintain the pro-survival Akt signaling that protects osteocytes from glucocorticoid-induced apoptosis. The preservation of bone microarchitecture and the favorable shift in bone turnover markers indicate that this approach addresses the disease at its mechanistic root rather than merely treating symptoms. Importantly, our results demonstrate that spatial restriction of therapeutic effects to the femoral joint prevents interference with systemic glucocorticoid signaling. This is crucial for patients who require corticosteroid therapy for life-threatening conditions such as autoimmune diseases, organ transplantation, or severe inflammatory disorders. The ability to protect specific anatomical sites from glucocorticoid toxicity while maintaining therapeutic immunosuppression represents a paradigm shift in managing corticosteroid-associated complications.

Regarding the clinical feasibility of our delivery and photoactivation strategy, both components employ established clinical procedures. Intrafemoral injection is routinely performed for bone marrow aspiration under local anesthesia with minimal invasiveness^30^. NIR illumination can be delivered transcutaneously using portable LED devices approved for clinical use, enabling non-invasive, painless activation in outpatient settings^31^. The light-gated control mechanism provides clinical advantages through spatial precision via targeted illumination and temporal control via on-demand activation, with the ability to immediately terminate therapy by withholding light if needed. Beyond SAON, the modular platform architecture enables rapid adaptation to diverse localized musculoskeletal disorders, including osteoarthritis, fracture healing, and degenerative disc disease, positioning this technology as a clinically viable strategy for targeted orthopedic gene therapy.

However, we acknowledge several limitations and areas for future development. While our current study focused on local delivery, systemic administration with tissue-specific targeting would broaden therapeutic applications. Engineering UCNPs with targeting moieties such as bone-seeking bisphosphonates or antibodies against tissue-specific markers could enable intravenous delivery with site-specific accumulation. Additionally, while 980 nm NIR provides good tissue penetration, exploration of alternative wavelengths in the second NIR window (1000–1350 nm) might further improve deep tissue activation^32^. The integration of artificial intelligence and machine learning approaches could accelerate future iterations of the paCas13d system. AI-driven protein design tools could identify novel split sites or engineer variants with improved properties such as enhanced stability, reduced immunogenicity, or altered PAM requirements^33,34^. Similarly, machine learning algorithms could optimize UCNP composition and surface chemistry for specific tissue targets or delivery routes^35^.

In conclusion, our photoactivatable Cas13d system represents a convergence of multiple cutting-edge technologies to address a fundamental challenge in gene therapy: achieving precise spatiotemporal control over therapeutic effects. By demonstrating its efficacy in preventing SAON, we provide proof-of-concept for a new class of RNA therapeutics that could transform the treatment of localized diseases. The modular nature of the platform, combined with its non-invasive activation and reversible effects, positions it as a versatile tool for precision medicine. As we advance toward clinical translation, this technology holds promise not only for preventing glucocorticoid-induced complications but also for addressing a broad spectrum of diseases where localized RNA modulation could provide therapeutic benefit. The future integration of emerging technologies in protein engineering, nanomaterials, and artificial intelligence will likely expand the capabilities and applications of this platform, opening new frontiers in spatially-targeted molecular therapeutics.

## Methods

### Ethical statement

All animal experiments were conducted in accordance with Guidelines for Care and Use of Laboratory Animals and approved by the Institutional Animal Care and Use Committee of Tianjin Hospital. The approval number for this study is 2021YLS047.

### Split Cas13d system design and construction

The three-dimensional structure of full-length RfxCas13d was predicted using AlphaFold Server (https://alphafoldserver.com/) to identify optimal split sites, and the resulting structure files were visualized using PyMOL^36^. Ten split sites (84, 303, 342, 405, 451, 542, 582, 641, 657, and 691) were strategically selected based on solvent accessibility, structural flexibility, and minimal disruption to functional domains. Split fragments were human codon-optimized and synthesized (GenScript, Nanjing, China).

N-terminal Cas13d fragments were fused to CIBN via a flexible (GGGGS)₃ linker and cloned into pcDNA3.1^(+)^ vector (Invitrogen™, V790-20) with nuclear export signal (NES). C-terminal fragments were fused to CRY2PHR and cloned with nuclear localization signals (NLS). Guide RNAs targeting human *CXCR4*, *ANXA4*, *HOTTIP*, and mouse *Tet3* were cloned into CasRx gRNA backbone vector (addgene #109053). Guide RNAs targeting human *CXCR4*, *ANXA4*, *HOTTIP*, and mouse *Tet3* were designed using the Cas13 Design computational platform Cas13 Design (https://cas13design.nygenome.org/)^37^. This platform employs machine learning algorithms trained on large-scale Cas13 screening datasets to predict on-target knockdown efficiency while minimizing off-target effects. For each target gene, multiple candidate crRNAs were computationally designed and ranked by predicted efficacy scores. Top-ranked sequences were synthesized and used in all subsequent cellular and in vivo experiments, and all guide sequences were listed in Supplementary Table 1.

For programmable A-to-I RNA base editing, catalytically inactive split Cas13d variants were generated by introducing dual inactivating mutations in each HEPN nuclease domain: R239A/H244A in the N-terminal fragment (HEPN-I domain) and R858A/H863A in the C-terminal fragment (HEPN-II domain). The catalytically dead C-terminal fragment was further engineered by C-terminal fusion to the catalytic domain of human ADAR2 via a flexible (GGGGS)₃ linker to generate the photoactivatable dead Cas13d-ADAR2 deaminase (padCas13d-ADAR2_DD_) base editing system.

Firefly luciferase reporters containing target sequences were constructed in pGL3-Basic vector (Promega). For A-to-I editing validation, a premature UAG stop codon was introduced at position 417 of firefly luciferase. Renilla luciferase control was driven by EF-1α promoter for normalization.

### Upconversion nanoparticle synthesis and characterization

The blue-emitting UCNPs core (NaYF₄:20%Yb/0.5%Tm) were synthesized via a thermal decomposition method^38,39^. Lanthanide precursors (0.795 mmol Y(CH₃CO₂)₃, 0.2 mmol Yb(CH₃CO₂)₃, 0.005 mmol Tm(CH₃CO₂)₃) were mixed with 6 mL oleic acid and 15 mL 1-octadecene, and then the mixture was slowly heated to 110 °C under vacuum with magnetic stirring for 1 h. Subsequently, the temperature of the mixed solution was increased to 160 °C and maintained for 45 min under an argon atmosphere. After cooling to 50 °C, a methanol solution (8 mL) containing 2.5 mmol NaOH and 4 mmol NH₄F was added, followed by stirring for 30 min. Then, the reaction was heated to 110 °C under vacuum for 10 min to eliminate methanol, followed by heating to 300 °C and maintaining for 1.5 h under an argon atmosphere. UCNPs were purified by ethanol precipitation and centrifugation (3823 × *g*, 10 min), then redispersed in cyclohexane for further use.

To reduce surface-related quenching and preserve emission intensity, an inert NaYF₄ shell was epitaxially deposited on the as-prepared UCNPs core. In brief, 0.4 mmol Y(CH₃CO₂)₃ was combined with oleic acid (6 mL) and 1-octadecene (15 mL), degassed at 110 °C for 1 h, and then heated to 160 °C for 45 min under an inert atmosphere to obtain a clear, uniform solution. After the mixture was cooled to 50 °C, UCNP cores (1 mmol, dispersed in 4 mL cyclohexane) were added. The suspension was vacuum-treated at 110 °C for 20 min to remove cyclohexane. A methanolic precursor solution (6 mL) containing NaOH (1 mmol) and NH₄F (1.6 mmol) was then introduced at 45 °C, followed by stirring for 30 min. Methanol was subsequently removed under vacuum at 110 °C for another 20 min. The reaction temperature was ramped to 300 °C under inert gas and maintained for 1.5 h. After cooling to room temperature, the core-shell UCNPs were collected, washed three times with ethanol, and finally redispersed in 4 mL cyclohexane for subsequent use.

The surface modification of polyethylenimine (PEI) was carried out with a two-step ligand-exchange method^40^. The hydrophobic UCNPs (4 mL cyclohexane dispersion) were precipitated with ethanol and redispersed in 20 mL HCl solution (0.1 M). The mixture was sonicated at 45 °C for 1 h to remove oleate ligands, then the ligand-free UCNPs were collected by centrifugation and redispersed in H_2_O (1 mL). Subsequently, the PEI (50 mg, Mw ~25,000) was dissolved in 9 mL deionized water, and the pH was adjusted to around 11 using 0.1 M NaOH. Ligand-free UCNPs (1 mL) were then added dropwise under continuous stirring and allowed to react for 2 h. The resulting dispersion was mixed with 10 mL diethylene glycol (DEG) and heated at 105 °C for 1 h, followed by transfer into a Teflon-lined autoclave and incubation at 160 °C for 2 h. The UCNPs-PEI were harvested by centrifugation, sequentially washed with ethanol and deionized water, and finally stored in water at 4 °C for further use.

### Dynamic light scattering (DLS) and zeta potential analysis

Hydrodynamic size distribution and zeta potential of ligand-free UCNPs and UCNPs-PEI were measured using a Zetasizer Nano ZS (Malvern Panalytical) at 25 °C. For DLS, samples were dispersed in ultrapure water (0.1 mg/mL) and sonicated for 5 min prior to measurement. For zeta potential measurements, samples were dispersed in PBS (pH 7.4) at the same concentration (0.1 mg/mL) and sonicated for 5 min before measurement. Each sample was analyzed in triplicate.

### Cell culture and transfection

HEK293T cells were obtained from ATCC (CRL-11268). MLO-Y4 osteocyte-like cells were generously provided by Dr. Lynda Bonewald (University of Missouri-Kansas City)^41^. HEK293T cells were maintained in Dulbecco’s Modified Eagle’s Medium (DMEM, Gibco) supplemented with 10% fetal bovine serum (FBS, Lonsera) and 1× penicillin/streptomycin (Gibco)^42^. MLO-Y4 cells were maintained in α-modified essential medium (Gibco) supplemented with 5% FBS and 5% calf serum. All cells were cultured at 37 °C in a humidified atmosphere with 5% CO_2_^43^.

For conventional blue light-activated experiments, cells were transfected using Entranster™-H4000 (Engreen Biosystem) according to manufacturer’s instructions. For NIR-activated experiments, UCNPs-PEI nanoparticles served as both transfection vectors and photon transducers. For three-plasmid systems, an equimolar ratio (1:1:1) of N-fragment, C-fragment, and crRNA was employed. In four-plasmid systems, such as those used for dual-luciferase or mCherry reporter assays, a molar ratio of 1:1:1:0.5 (N-fragment:C-fragment:crRNA:reporter) was adopted. UCNPs-PEI nanoparticles (1 mg/mL in 10 mM HEPES, pH 7.4) were mixed with plasmid DNA encoding the split paCas13d components at nitrogen-to-phosphate (N/P) ratios of 0–40. After systematic optimization, we determined that N/P = 30 provided optimal transfection efficiency with minimal cytotoxicity. The mixture was vortexed briefly and incubated for 30 min at room temperature to allow complex formation. The resulting UCNPs-PEI@DNA complexes were applied to cells in serum-free medium for 6 h, followed by replacement with complete medium to ensure optimal transfection efficiency and cell viability. Optogenetic activation was initiated 16 h post-transfection using either blue light or NIR illumination according to experimental design. TET3-specific siRNA and control scramble siRNA were synthesized by Biotend based on published studies (Supplementary Table 2)^16^. Transfections were performed using Lipofectamine 2000 Transfection Reagent according to the manufacturer’s protocol.

### Optogenetic illumination systems

A custom LED array was constructed using ten 470 nm LEDs (1 W@300 mA, EPILEDs) connected in series, with two series connected in parallel and mounted on a circuit board^39^. Light intensity was set to 0.002 W/cm^2^ and controlled by a high-precision programmable DC power supply (RIGOL) for pulsed illumination (3 s on/60 s off cycles). Light power was measured using a TP100 Power Meter (Cnilaser).

A 980 nm NIR laser (Cnilaser, China) was used at 1.3 W/cm^2^ intensity with identical pulsing parameters. For in vivo experiments, NIR illumination was applied through a fiber optic cable positioned 5 mm above the injection site.

For spatial precision experiments, cells on 35 mm glass-bottom dishes were subjected to half-field illumination using photomasks. After 72 h of pulsed illumination, live-cell fluorescence imaging was performed directly using confocal microscopy (Olympus IX83).

### Luciferase reporter assays

Dual-luciferase assays were performed using the Dual-Luciferase Reporter Assay System (Promega) according to manufacturer’s protocol. Luminescence was measured using a GloMax Navigator Microplate Luminometer (Promega). Relative luciferase activity was calculated as firefly/Renilla luminescence ratio.

### RNA extraction and qRT-PCR

Total RNA was extracted using TRIzol reagent (Invitrogen) and reverse transcribed using PrimeScript RT Master Mix (Vazyme). Quantitative PCR was performed using AceQ qPCR SYBR Green Master Mix (Vazyme) on a CFX96 Real-Time System (Bio-Rad). Gene expression was normalized to GAPDH using the 2^−(ΔΔCt)^ method. Primer sequences were provided in Supplementary Table 3.

### RNA sequencing and analysis

Total RNA was extracted 48 h post-illumination. RNA-seq libraries were prepared using NEBNext Ultra RNA Library Prep Kit and sequenced on Illumina NovaSeq 6000 platform. Reads were aligned to human genome (hg38) using STAR aligner. Differential expression analysis was performed using DESeq2 with FDR < 0.05 and |log₂(FC)| > 1 as significance thresholds.

### Sanger sequencing

Target regions were PCR amplified and cloned into pMD19-T vector (TaKaRa). Individual clones were sequenced using ABI 3730xl DNA Analyzer to confirm A-to-I editing events.

### Protein analysis

Cells were lysed in RIPA buffer supplemented with protease and phosphatase inhibitors. Proteins were separated by SDS-PAGE and transferred to PVDF membranes. Primary antibodies used: TET1 (1:500, MilliporeSigma #SAB2700730), TET2 (1:1000, ProteinTech #21207-1-AP), TET3 (1:500, Abcam #ab153724), PTEN (1:1000, CST #9188), phospho-Akt (Ser473) (1:1000, CST #4060), Akt (1:1000, CST #4691), Bcl-2 (1:1000, CST #15071), Bax (1:1000, Abcam #ab32503), cleaved Caspase-3 (1:1000, CST #9664), Histone H3 (1:2000, CST #9715), β-actin (1:10000, Millipore #A5441), Tubulin (1:5000, CST #2146). Signals were detected using ECL substrate and quantified using ImageJ software. Nuclear and cytoplasmic fractions were separated using NE-PER Nuclear and Cytoplasmic Extraction Reagents (Thermo Fisher) according to manufacturer’s protocol. All of the unprocessed scans of the blots were shown in the Source Data file.

### 5hmC dot-blot assay

Genomic DNA was extracted using DNeasy Blood & Tissue Kit (Qiagen). DNA (125–1000 ng) was denatured with 1 M NaOH, neutralized with ammonium acetate, and spotted onto Hybond-N+ membrane (GE Healthcare). Membranes were probed with 5hmC antibody (1:10000, Active Motif #39769) overnight at 4 °C, followed by HRP-conjugated secondary antibody (1:2000, Abmart). Signals were detected using ECL substrate and quantified using ImageJ.

### TUNEL staining

Apoptosis was assessed using In Situ Cell Death Detection Kit (Roche) according to manufacturer’s instructions. Cells were counterstained with DAPI (100 μg/mL, Solarbio) and visualized using confocal microscopy (Olympus IX83). At least 500 cells were analyzed per condition using systematic random sampling.

### Cell viability assay

Cell viability was measured using Cell Counting Kit-8 (CCK-8, Dojindo) according to manufacturer’s protocol. Absorbance was measured at 450 nm using a microplate reader.

### Animal experiments and UCNPs-PEI@paCas13d intervention

Twelve-week-old male BALB/c mice were randomly divided into groups (*n* = 5 per group) and housed under standard specific pathogen-free conditions with a 12 h light/12 h dark cycle, an ambient temperature of 22 ± 2 °C, and relative humidity of 50–60%, with free access to food and water. The SAON model was established as previously described, with mice receiving 4 mg/L oral dexamethasone in drinking water for 90 days^23^. UCNPs-PEI@paCas13d complexes (0.625 mg/kg total DNA) were injected weekly into the medullary cavity of distal femur using a Hamilton syringe with 30-gauge needle under isoflurane anesthesia. NIR illumination (980 nm, 13 mW/mm²) was applied daily for 60 min starting 24 h post-injection throughout the experimental period.

### Micro-CT and histological analysis

Animals were euthanized, and femurs were immediately harvested and fixed in 4% paraformaldehyde. Micro-CT scanning was subsequently performed using a high-resolution μCT system (SkyScan 1276, Bruker) with the following imaging parameters: voxel resolution of 17.5 μm, source voltage of 70 kV, current of 200 μA, and integration time of 498 ms. A defined region of interest adjacent to the endocortical surface in the distal femoral epiphysis was selected, and trabecular microarchitecture parameters—including bone volume fraction (BV/TV), trabecular number (Tb.N), trabecular thickness (Tb.Th), and trabecular separation (Tb.Sp), were quantitatively analyzed using CTAn software (Bruker). Following a micro-CT scan, femurs were decalcified in 10% EDTA, embedded in paraffin, and sectioned at 5 μm thickness. Tissue sections were stained with hematoxylin and eosin (H&E) and evaluated independently by two experienced pathologists blinded to the experimental groups. Osteonecrosis was histologically defined by the presence of empty lacunae, pyknotic nuclei, or degeneration of bone marrow.

### Bioluminescence imaging

In vivo bioluminescence was monitored using IVIS Spectrum system (PerkinElmer) following luciferin injection (150 mg/kg, i.p.). Images were acquired with 1 min exposure time and analyzed using Living Image software. For ex vivo organ imaging of UCNPs-PEI, mice were euthanized at designated time points (Days 1, 3, 5, 7, 10), and major organs (heart, liver, spleen, lung, kidney) and femur were harvested, rinsed with PBS, and imaged under identical acquisition settings.

### Immunofluorescence staining

Cells on coverslips were fixed with 4% paraformaldehyde for 15 min, permeabilized with 0.3% Triton ×-100 for 10 min, and blocked with 5% BSA in PBS for 1 h. Cells were incubated overnight at 4 °C with mouse anti-FLAG antibody (1:200, MilliporeSigma #F1804) for Cas13d-N and rabbit anti-HA antibody (1:200, CST #3724) for Cas13d-C. Following PBS washes, cells were incubated with Alexa Fluor 488-conjugated anti-mouse IgG (1:500, Invitrogen #A-10680) and Alexa Fluor 594-conjugated anti-rabbit IgG (1:500, Invitrogen #A-11012) for 1 h at room temperature. Nuclei were stained with DAPI (1 μg/mL) for 5 min. Images were acquired using confocal microscope with sequential scanning mode and analyzed using ImageJ software.

### Biochemical and hematological analysis

Blood samples were collected via cardiac puncture at experimental endpoints. Complete blood count analysis was performed using an automated veterinary hematology analyzer (BC-5000VET, Mindray). Serum biochemistry parameters, including liver function markers (ALT and AST) and kidney function indicators (CTEA), were assessed using an automated veterinary biochemistry analyzer (BS-240VET, Mindray) to evaluate systemic toxicity. Serum osteocalcin (OCN) and C-terminal telopeptide of type I collagen (CTX-1) were measured using mouse-specific ELISA kits (CUSABIO) according to manufacturer’s protocols.

### Statistics and reproducibility

All experiments were performed at least 3 biologically independent experiments, the results were shown as means ± SD. GraphPad Prism (Version 10.3.1) software was used for statistical analysis. Data between groups were compared using one-way or two-way ANOVA with multiple comparisons. Differences were considered statistically significant at *P* < 0.05. *n* and *P* values were described in the figure legends.

### Reporting summary

Further information on research design is available in the Nature Portfolio Reporting Summary linked to this article.

## Supplementary information

Supplementary Information

Reporting Summary

Transparent Peer Review file

## Source data

Source Data

## Data Availability

All data supporting the findings of this study are available within the paper and its Supplementary Information. The RNA sequencing data used in this study are deposited in the NCBI GEO database with accession number GSE325787. Source data are provided with this paper.
