# Supplementary material for: Photoactivatable CRISPR/Cas13d via upconversion nanoparticles for deep tissue RNA engineering and orthopedic therapy
Source: Nat Commun. 2026 Apr 20;17:5216. doi: 10.1038/s41467-026-72181-6 (PMC13261002; doi:10.1038/s41467-026-72181-6)

Supplementary Information

Photoactivatable CRISPR/Cas13d via upconversion nanoparticles for deep tissue RNA engineering and orthopedic therapy

Jie Zhao[1#], Jingyu Zhang[2#], Miaomiao Gao[1#], Zukang Miao[1], Yang Zhang[1,3], Yue Guo[1,3], Zhengrui Fan[1], Jinglin Tian[1], Lu Yang[1], Ning Jiang[1], Jianxiong Ma[1,3], Jun Jiao[4*], Jinbin Pan[5,6*], Xinlong Ma[1,3*]

1. Department of orthopedic, Tianjin Hospital, Tianjin University, Tianjin, 300211, China

2. Department of Bone and Soft Tissue Oncology, Tianjin Hospital, Tianjin University, Tianjin, 300211, China

3. Tianjin Key Laboratory of Orthopedic Biomechanics and Medical Engineering, Tianjin Hospital, Tianjin 300050, China

4. Tianjin Key Laboratory of Tumor Microenvironment and Neurovascular Regulation, School of Medicine, Nankai University, Tianjin, 300071, China

5. Department of Radiology, Tianjin Key Lab of Functional Imaging & Tianjin Institute of Radiology, Tianjin Medical University General Hospital, Tianjin, 300052, China

6. Athinoula A. Martinos Center for Biomedical Imaging, Department of Radiology, Massachusetts General Hospital, Harvard Medical School, Charlestown, MA 02129, USA

[#]These authors contributed equally.

*Correspondence: Xinlong Ma(maxinlong8686@yeah.net); Jinbin Pan (panjinbin@tmu.edu.cn); Jun Jiao (junjiao@nankai.edu.cn)

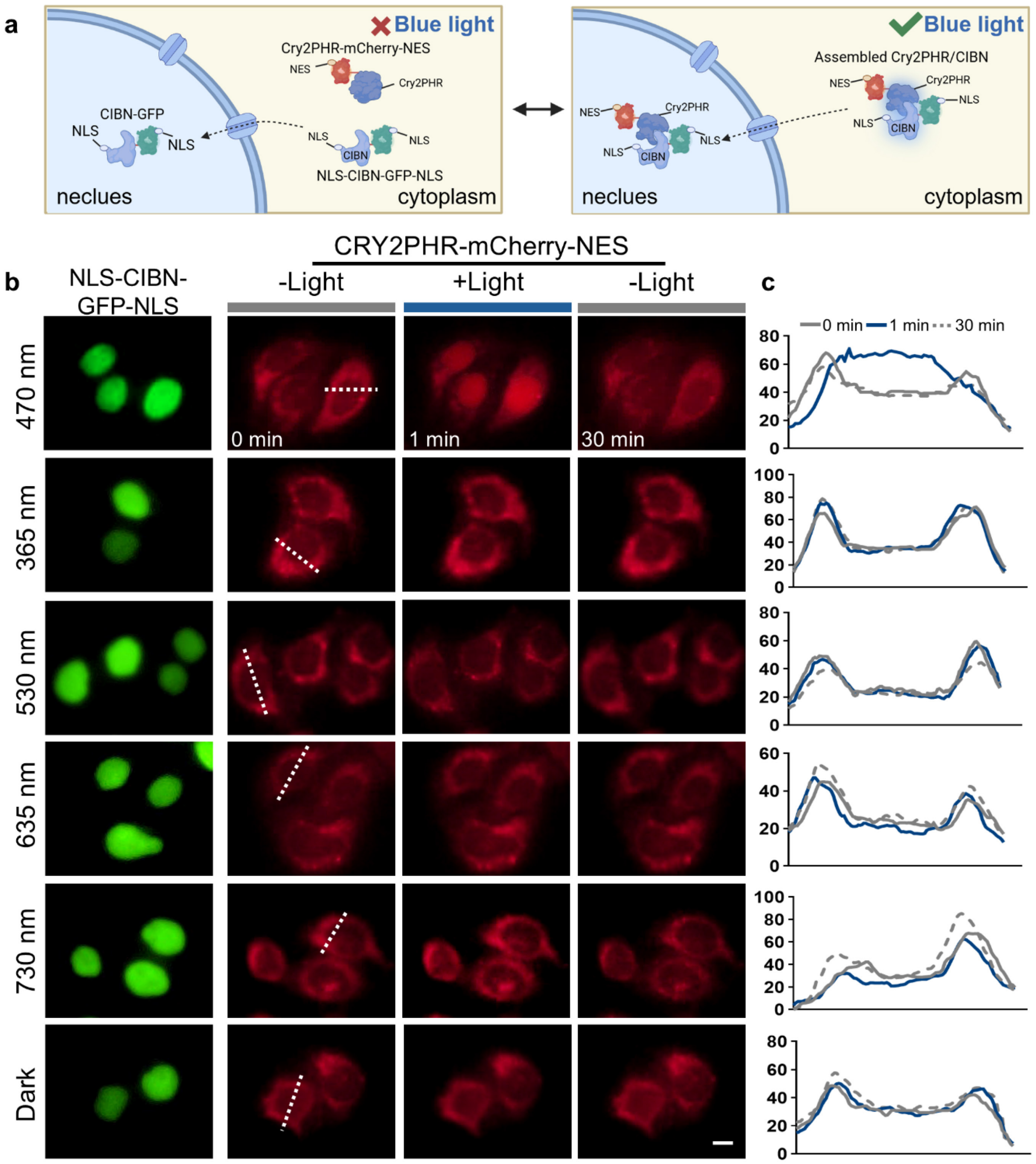

Supplementary Fig. 1 Wavelength specificity and reversibility of CRY2PHR/CIBN light-inducible dimerization

(a) Schematic illustration of the nucleus-cytoplasm translocation assay. Nuclear-localized NLS-CIBN-GFP-NLS (green) and cytoplasmic CRY2PHR-mCherry-NES (red) undergo heterodimerization upon blue light (470 nm) illumination, inducing CRY2PHR-mCherry nuclear translocation. NLS: nuclear localization signal; NES: nuclear export signal. Schematic created with BioRender.com. **(b)** Representative live-cell imaging of HEK293T cells expressing NLS-CIBN-GFP-NLS and CRY2PHR-mCherry-NES exposed to different wavelengths (470 nm, 365 nm, 530 nm, 635 nm, 730 nm) or maintained in darkness (Dark). Left column: NLS-CIBN-GFP-NLS nuclear localization. Middle columns: CRY2PHR-mCherry distribution before illumination (−Light, 0 min), 1 minute after light onset (+Light, 1 min), and 30 minutes after light cessation (−Light, 30 min). Scale bar, 10 μm. **(c)** Quantitative line scan analysis of CRY2PHR-mCherry fluorescence intensity over time for each wavelength condition shown in **(b)**.

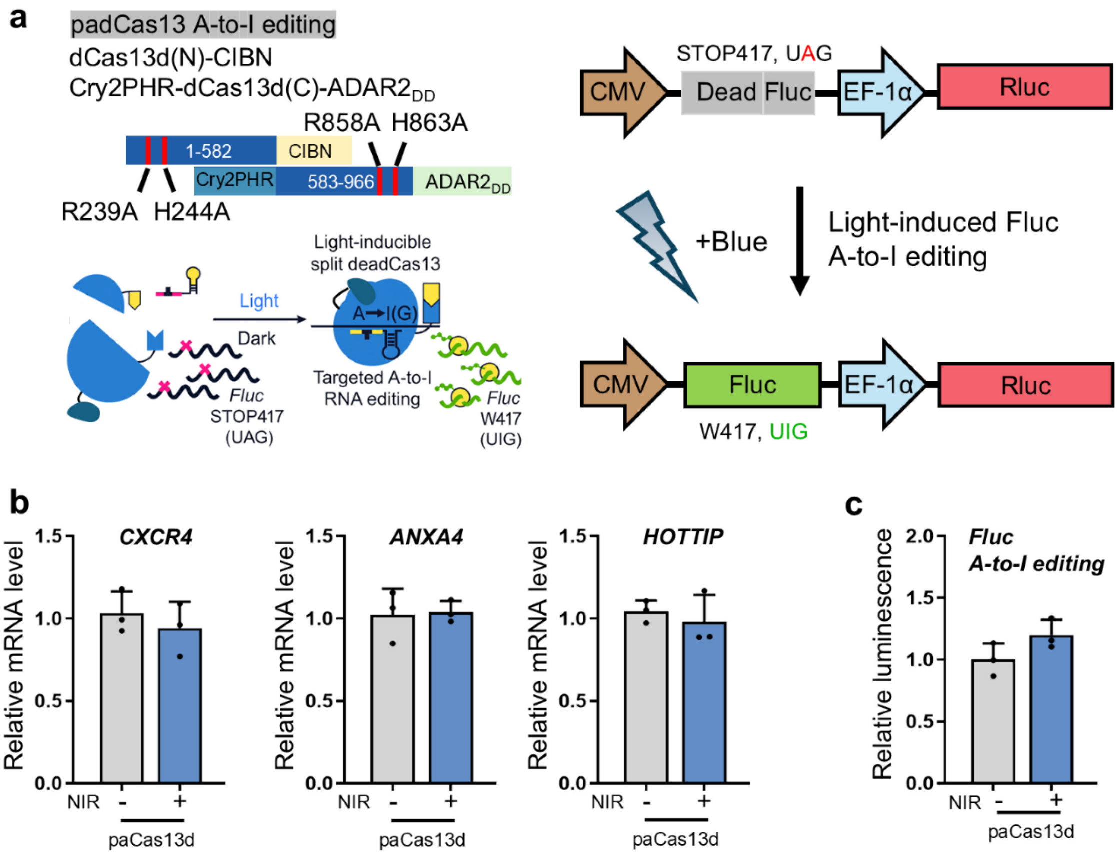

Supplementary Fig. 2 Engineering of padCas13d for precision RNA base editing

(a) Schematic representation of the photoactivatable deadCas13d (padCas13d) system engineered for A-to-I RNA base editing. The catalytically inactive dCas13d is split at the optimized N582/C583 site, with the N-terminal fragment (1-582) fused to CIBN and the C-terminal fragment (583-966) fused to CRY2PHR and the ADAR2 deaminase domain ($ADAR2_{DD}$). Key catalytic residues R239A and H244A in the N-terminal HEPN domain and R858A and H863A in the C-terminal HEPN domain are mutated to abolish nuclease activity while preserving RNA binding. Upon blue light illumination (~470 nm), CRY2PHR undergoes conformational changes enabling heterodimerization with CIBN, reconstituting the split padCas13d-$ADAR2_{DD}$ complex for targeted A-to-I editing. **(b)** Quantitative RT-PCR analysis of endogenous transcript levels (*CXCR4*, *ANXA4*, *HOTTIP*) in HEK293T cells transfected with paCas13d and exposed to NIR light (980 nm) in the absence of upconversion nanoparticles. **(c)** Dual-luciferase reporter assay measuring A-to-I editing efficiency in cells transfected with padCas13d and exposed to NIR light without upconversion nanoparticles. For panel **b** and **c**, n = 3 independent experiments. Data are presented as mean ± SD.

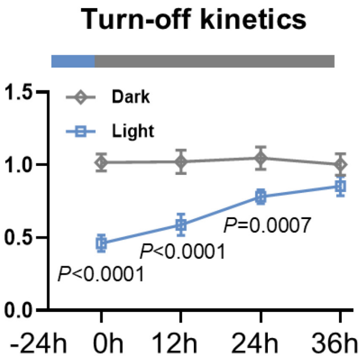

Supplementary Fig.3 Temporal reversibility and turn-off kinetics of paCas13d

Time-course analysis demonstrating the reversible nature of paCas13d-mediated RNA knockdown. HEK293T cells transfected with paCas13d targeting *CXCR4* were subjected to continuous blue light illumination for 24 hours, followed by light withdrawal. Relative *CXCR4* mRNA levels were quantified by RT-qPCR at indicated time points. Time point 0 h indicates the moment of light withdrawal. Data are presented as mean ± SD (n = 3 independent experiments). *P* values were calculated by two-way ANOVA with multiple comparisons.

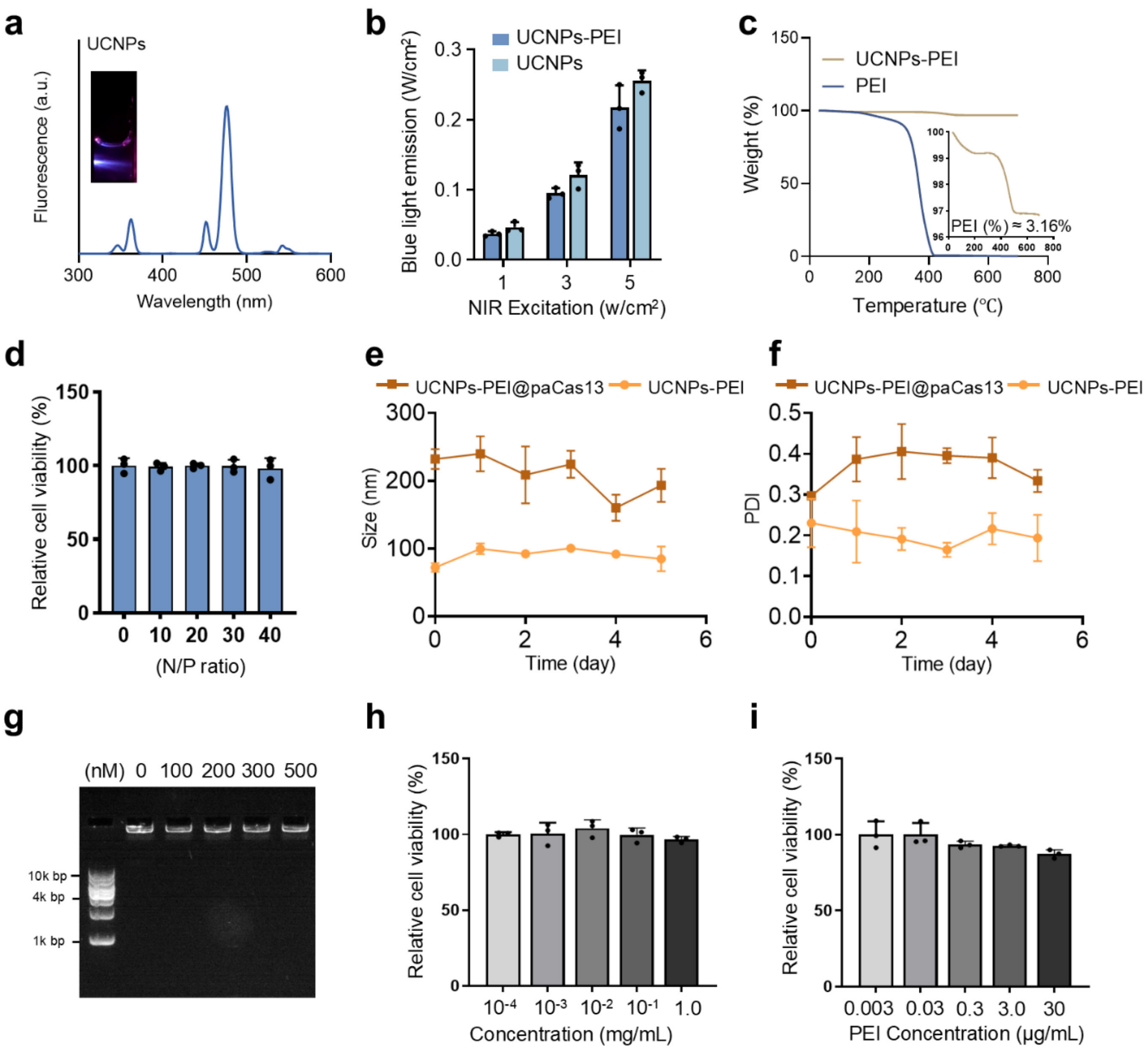

Supplementary Fig.4 Physicochemical and biological characterization of UCNPs-PEI nanoparticles

(a) Upconversion emission spectrum of UCNPs under 980 nm NIR excitation showing characteristic blue emission centered at ~470 nm. Inset: photograph of UCNPs under 980 nm laser illumination. **(b)** Quantification of blue light emission intensity from UCNPs-PEI and ligand-free UCNPs at different NIR excitation power densities (1, 3, 5 W/cm²). **(c)** Thermogravimetric analysis (TGA) of UCNPs-PEI. Inset: magnified view of the weight loss region. **(d)** Cell viability of HEK293T cells transfected with UCNPs-PEI@paCas13d complexes at N/P ratios from 0 to 40. **(e)** Hydrodynamic diameter of UCNPs-PEI and UCNPs-PEI@paCas13d measured by dynamic light scattering (DLS) over 5 days. **(f)** Polydispersity index (PDI) of UCNPs-PEI and UCNPs-PEI@paCas13d over 5 days. **(g)** Agarose gel electrophoresis of UCNPs-PEI@paCas13d complexes (N/P=30) after incubation in NaCl solutions of increasing ionic strength (0-500 mM) for 24 hours, demonstrating resistance to salt-induced dissociation. **(h)** Cell viability of HEK293T cells exposed to UCNPs-PEI at concentrations ranging from 10^{-4} to 1.0 mg/mL for 6 hours. **(i)** Cell viability of HEK293T cells exposed to free branched PEI (25 kDa) at concentrations equivalent to PEI content in UCNPs-PEI formulations (0.003-30 μg/mL) for 6 hours. For panel **b, d, e, f, h** and **i**, n = 3 independent experiments. Data are presented as mean ± SD.

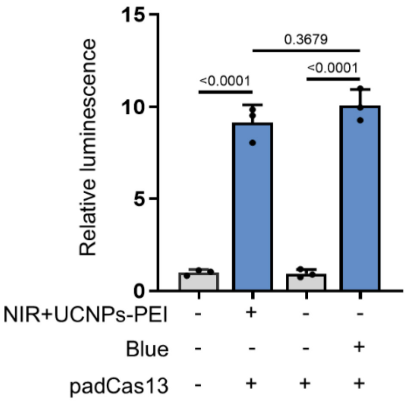

Supplementary Fig.5 Equivalent A-to-I editing efficiency between NIR-mediated and direct blue light activation

Dual-luciferase reporter assay comparing A-to-I editing efficiency of padCas13d-ADAR2$_{DD}$ system under NIR-excited UCNPs (NIR-UCNPs) versus direct blue light illumination. Data are presented as mean ± SD (n = 3 independent experiments). *P* values were calculated by one-way ANOVA with multiple comparisons.

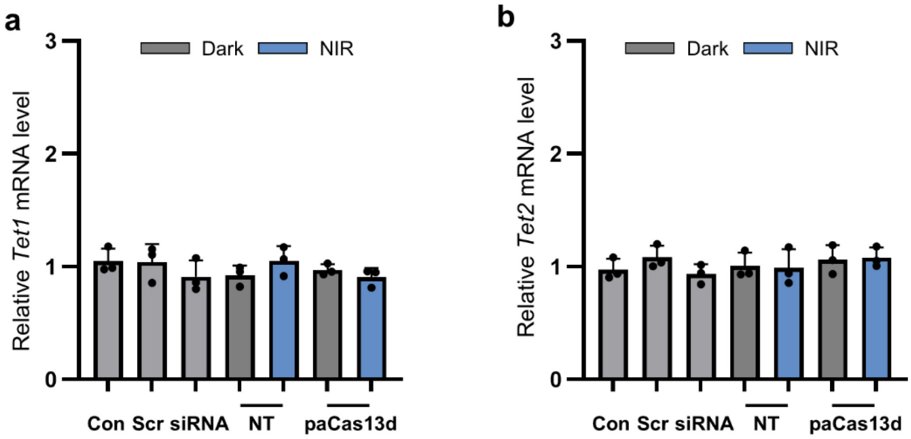

Supplementary Fig.6 Specificity of TET3-targeted paCas13d system

(a) *Tet1* mRNA levels remain unchanged across all treatment conditions, including scramble siRNA (Scr), TET3-targeting siRNA, non-targeting paCas13d (NT), and TET3-targeting paCas13d with or without NIR activation. **(b)** *Tet2* mRNA expression is unaffected by TET3-targeted interventions, demonstrating the specificity of paCas13d approaches. Data are presented as mean ± SD (n = 3 independent experiments).

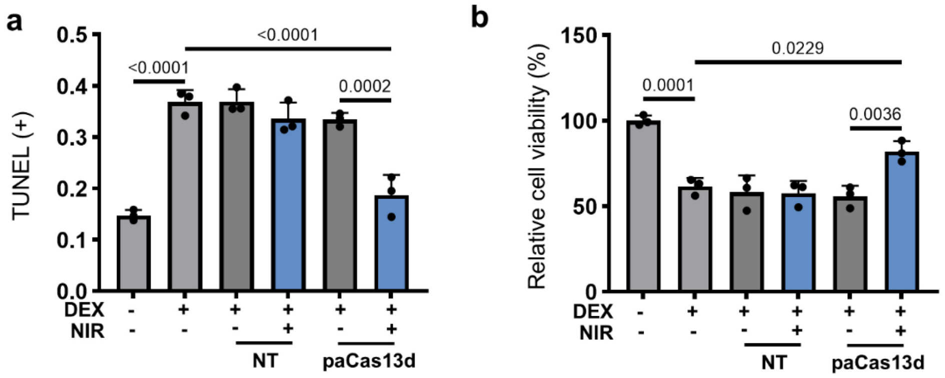

Supplementary Fig.7 Quantitative analysis of steroid-induced osteocyte apoptosis and cell viability

(a) Quantitative analysis of TUNEL-positive apoptotic cells in MLO-Y4 osteocytes following dexamethasone (1×10^{-6}M) treatment and paCas13d intervention. Systematic random sampling of at least 500 cells per condition reveals significant reduction in apoptosis from the dexamethasone group with NIR-activated TET3-targeting paCas13d treatment. Non-targeting controls show no protective effect. **(b)** Cell viability assessment by CCK-8 assay demonstrating restoration of osteocyte survival following NIR-activated paCas13d treatment. For panel **a** and **b**, n = 3 independent experiments. Data are presented as mean ± SD. *P* values were calculated by one-way ANOVA with multiple comparisons.

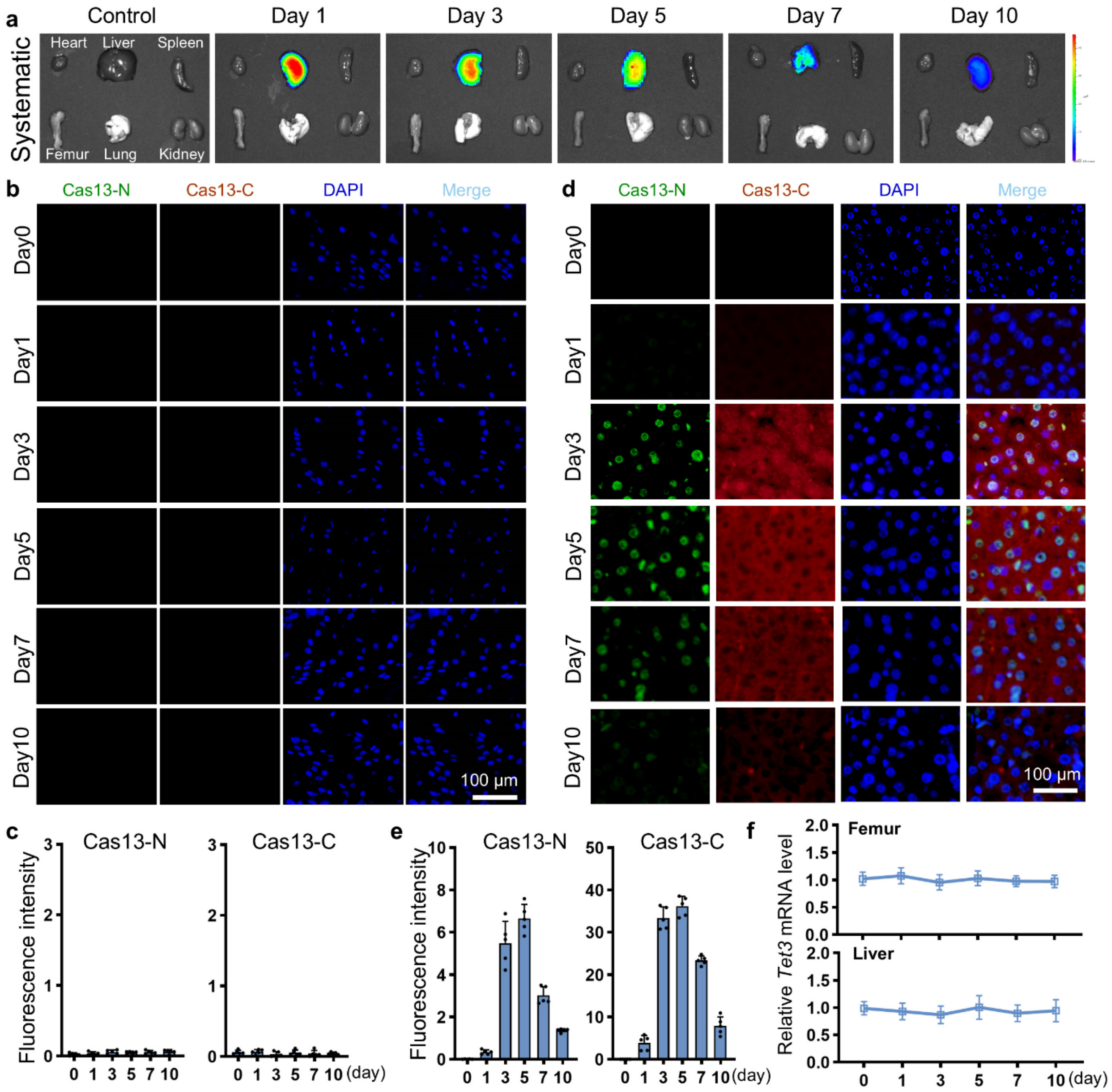

Supplementary Fig.8 Pharmacokinetics and biodistribution of UCNPs-PEI@paCas13d following systemic administration

(a) Representative in vivo imaging (heart, liver, spleen, femur, lung, kidney) following intravenous injection of UCNPs-PEI@paCas13d at Days 1, 3, 5, 7, and 10 post-injection. **(b,d)** Immunofluorescence staining of Cas13d-N (green) and Cas13d-C (red) in femoral bone tissue **(b)** and hepatic tissue **(d)** at indicated time points following intravenous administration. DAPI (blue) indicates nuclei. Scale bars = 100 μm. **(c,e)** Quantification of Cas13d-N and Cas13d-C fluorescence intensity in femoral **(c)** and hepatic **(e)** tissues over time. Data show mean fluorescence intensity in arbitrary units per timepoint across five animals. **(f)** Quantitative RT-PCR analysis of *Tet3* mRNA levels in femoral and hepatic tissues following intravenous administration. Data are presented as mean ± SD (n=5 animals per group).

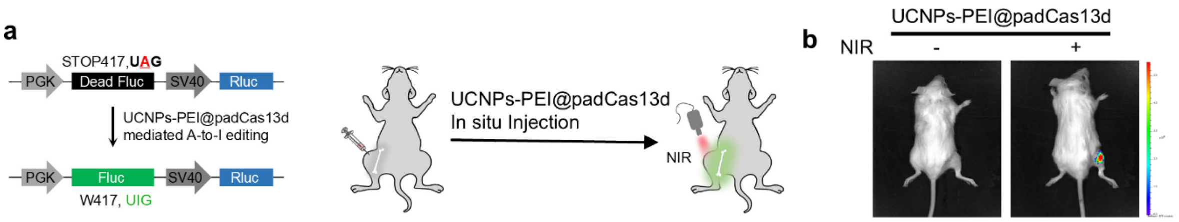

Supplementary Fig.9 Spatiotemporally controlled RNA editing in vivo with UCNPs-PEI@padCas13d system

(a) Experimental design for RNA editing in vivo with UCNPs-PEI@padCas13d system using the dual-luciferase A-to-I editing reporter (described in Supplementary Fig.2a) delivered via intrafemoral injection. **(b)** Bioluminescence imaging showing luciferase signals are exclusively detected in NIR-treated femurs, showing precise spatial restriction of RNA editing activity in living animals with UCNPs-PEI@padCas13d.

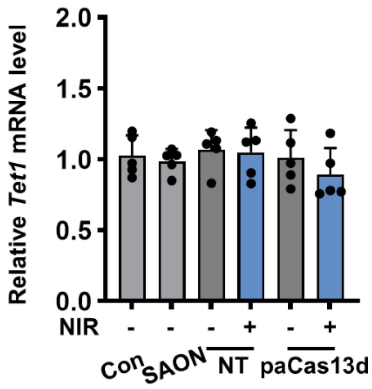

Supplementary Fig.10 TET3 targeting specificity without off-target effects in vivo
RT-qPCR analysis of TET1 mRNA expression in femoral tissue from different treatment groups. Data are presented as mean ± SD (n=5 animals per group).

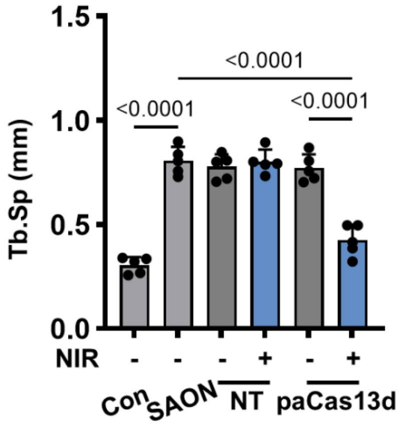

Supplementary Fig.11 Micro-CT quantitative analysis of trabecular bone microarchitecture

Trabecular separation (Tb.Sp) measurements from micro-CT analysis of distal femur. Data are presented as mean ± SD (n=5 animals per group). *P* values were calculated by one-way ANOVA with multiple comparisons.

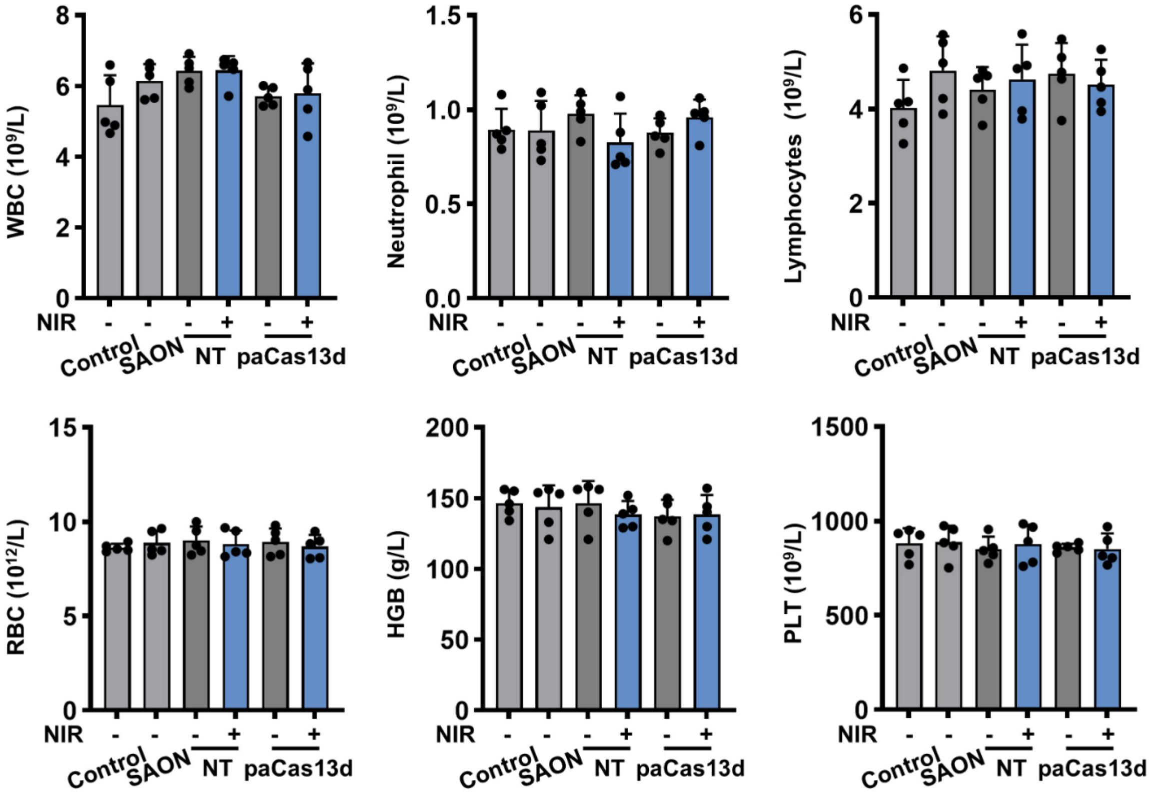

Supplementary Fig.12 Hematological safety profile analysis

Complete blood count analysis demonstrating systemic safety of UCNPs-PEI@paCas13d treatment. All hematological parameters including white blood cells (WBC), neutrophils, lymphocytes, red blood cells (RBC), hemoglobin (HGB), and platelets (PLT) remain no significant change across all treatment groups. Data are presented as mean ± SD (n=5 animals per group).

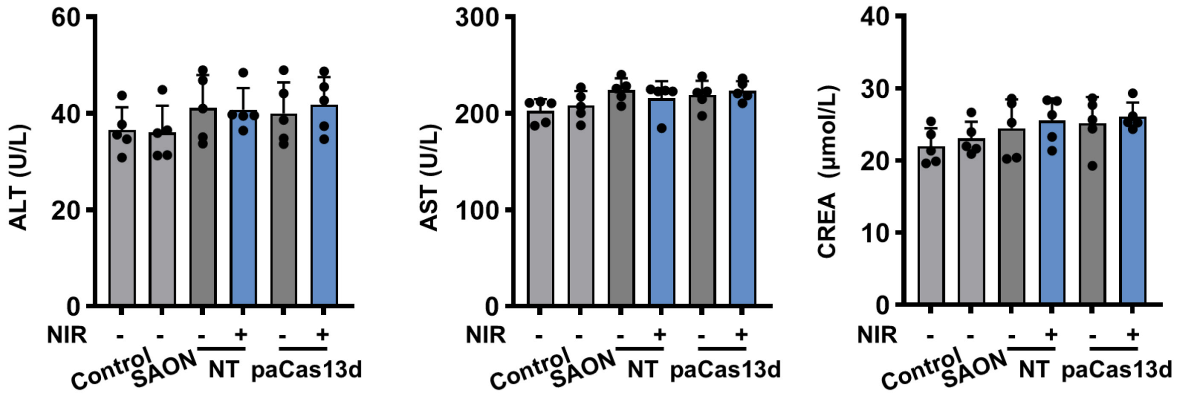

Supplementary Fig.13 Hepatic and renal function safety assessment

Serum biochemical markers evaluating liver and kidney function following UCNPs-PEI@paCas13d treatment. Alanine aminotransferase (ALT), aspartate aminotransferase (AST), and creatinine (CREA) levels remain no significant change across all experimental groups, indicating absence of hepatotoxicity or nephrotoxicity. Data are presented as mean ± SD (n=5 animals per group).

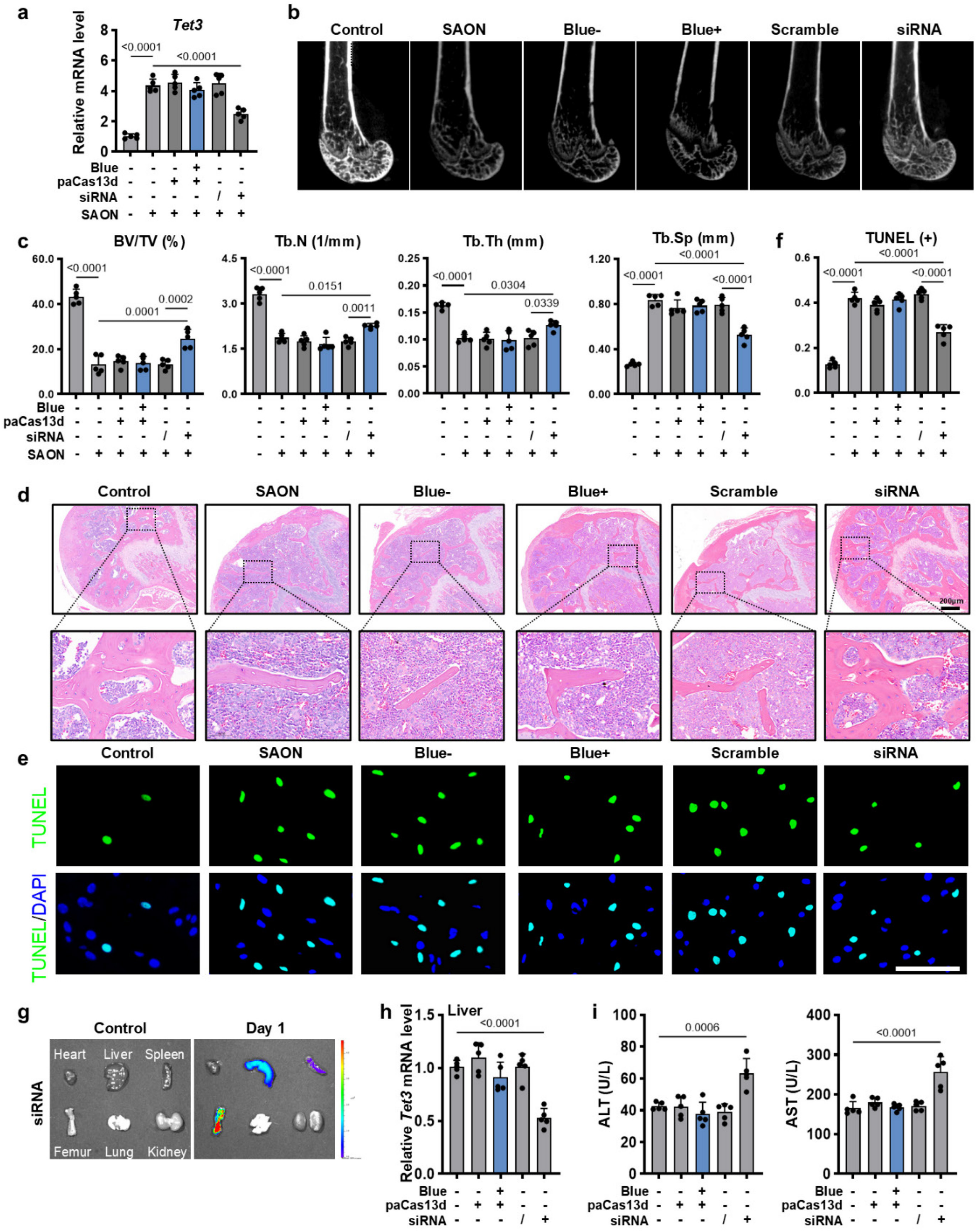

Supplementary Fig.14 Evaluation of blue light activation and siRNA-mediated *Tet3* knockdown in SAON model

(a) Quantitative RT-PCR analysis of femoral *Tet3* mRNA levels across treatment groups, including siRNA-mediated *Tet3* knockdown and direct blue light activation of paCas13d without UCNPs. **(/)** : scrambled siRNA control. **(b)** Representative micro-CT 3D reconstruction images of femur trabecular bone. **(c)** Quantitative micro-CT analysis of trabecular bone parameters including BV/TV, Tb.N, Tb.Th, and Tb.Sp. **(d)** Hematoxylin and eosin (H&E) staining of femur trabecular bone. Upper panels show low magnification overview; lower panels show high magnification of trabecular microarchitecture within boxed regions. Scale

bars = 200 μm. **(e)** Representative TUNEL staining (green) of femoral sections showing apoptotic osteocytes. DAPI (blue) counterstains nuclei. Scale bar = 50 μm. **(f)** Quantitative analysis of TUNEL-positive osteocytes. **(g)** In vivo organ imaging of siRNA at Day 1 post-injection. **(h)** Quantitative RT-PCR analysis of hepatic *Tet3* mRNA levels to assess potential off-target gene suppression across treatment groups. **(i)** Serum alanine aminotransferase (ALT) and aspartate aminotransferase (AST) levels measured as indicators of liver function. For panel **a, c, f, h** and **i**, n=5 animals per group. Data are presented as mean ± SD. *P* values were calculated by one-way ANOVA with multiple comparisons.

Supplementary Table 1. Guide sequences used in this study.

Name	Sequence
Fluc	CGATGGTTTGTATTCAGCCCATA
hCXCR4	CCAATGTAGTAAGGCAGCCAACA
hANXA4	ATGCACTTTACTATAGCCAGCAG
hLnc-HOTTIP	CCTTCCAGAACTAAGCCAGCCAT
Fluc A-to-I	CGATGTCGCCGCTGTGCAGC**C**AGCCGTCCTTGTCGATGAGAGCGTTTGTA
mCherry	AACTCCTTGATGATGGCCATGTT
mTet3	GATCAAGATAACAATCACGGCGT
Non-targeting	TCACCAGAAGCGTACCATACTC

Supplementary Table 2. Sequences of siRNA.

Name	Sequence
Tet3 siRNA	GCTCCAACGAGAAGCTATTTG
Scramble	ACGUGACACGUUCGGAGAATT

Supplementary Table 3. Sequences of qRT-PCR primers.

Name	Forward	Reverse
hCXCR4	ACTACACCGAGGAAATGGGCT	CCCACAATGCCAGTTAAGAAGA
hANXA4	GGAGGTACTGTCAAAGCTGCT	GGCAAGGACGCTAATAATGGC
hLnc-HOTTIP	CCTAAAGCCACGCTTCTTTG	TGCAGGCTGGAGATCCTACT
hGapdh	ACAACTTTGGTATCGTGGAAGG	GCCATCACGCCACAGTTTC
mTet3	TGCGATTGTGTCGAACAAATAGT	TCCATACCGATCCTCCATGAG
mTet2	AGAGAAGACAATCGAGAAGTCGG	CCTTCCGTACTCCCAAACTCAT
mTet1	ACACAGTGGTGCTAATGCAG	AGCATGAACGGGAGAATCGG
mGapdh	AGGTCGGTGTGAACGGATTTG	TGTAGACCATGTAGTTGAGGTCA

Note S1. Amino acid sequence of paCas13d system.

RfxCas13d (N): 1-582aa
MIEKKKSFAKGMGVKSTLVSGSKVYMTTFAEGSDARLEKIVEGDSIRSVNEGEAFSA
EMADKNAGYKIGNAKFSHPKGYAVVANNPLYTGPVQQDMLGLKETLEKRYFGESA
DGNDNICIQVIHNILDIEKILAEYITNAAYAVNNISGLDKDIIGFGKFSTVYTYDEFKDP
EHHRAAFNNNDKLINAIKAQYDEFDNFLDNPRLGYFGQAFFSKEGRNYIINYGNECY
DILALLSGLRHWVVHNNEEESRISRTWLYNLDKNLDNEYISTLNYLYDRITNELTNSF
SKNSAANVNYIAETLGINPAEFAEQYFRFSIMKEQKNLGFNITKLREVMLDRKDMSEI
RKNHKVFDSIRTKVYTMMDFVIYRYYIEEDAKVAAANKSLPDNEKSLSEKDIFVINLR
GSFNDDQKDALYYDEANRIWRKLENIMHNIKEFRGNKTREYKKKDAPRLPRILPAGR
DVSAFSKLMYALTMFLDGKEINDLLTTLINKFDNIQSFLKVMPLIGVNAKFVEEYAFF
KDSAKIADELRLIKSFARMGEPIADARRAMYIDAIRILGTNLSYDELKALADTFSLDEN
GNKLK

RfxCas13d (C): 583-967aa
KGKHGMRNFIINNVISNKRFHYLIRYGDPAHLHEIAKNEAVVKFVLGRIADIQKKQGQ
NGKNQIDRYYETCIGKDKGKSVSEKVDALTKIITGMNYDQFDKKRSVIEDTGRENAE
REKFKKIISLYLTVIYHILKNIVNINARYVIGFHCVERDAQLYKEKGYDINLKKLEEKG
FSSVTKLCAGIDETAPDKRKDVEKEMAERAKESIDSLESANPKLYANYIKYSDEKKAE
EFTRQINREKAKTALNAYLRNTKWNVIIREDLLRIDNKTCTLFRNKAVHLEVARYVH
AYINDIAEVNSYFQLYHYIMQRIIMNERYEKSSGKVSEYFDAVNDEKKYNDRLLKLL
CVPFGYCIPRFKNLSIEALFDRNEAAKFDKEKKKVSGNS

Cry2PHR
MKMDKKTIVWFRRDLRIEDNPALAAAAHEGSVFPVFIWCPEEEGQFYPGRASRWWM
KQSLAHLSQSLKALGSDLTLIKTHNTISAILDCIRVTGATKVVFNHLYDPVSLVRDHT
VKEKLVERGISVQSYNGDLLYEPWEIYCEKGKPFTSFNSYWKKCLDMSIESVMLPPP
WRLMPITAAAEAIWACSIEELGLENEAEKPSNALLTRAWSPGWSNADKLLNEFIEKQ
LIDYAKNSKKVVGNSTSLLSPYLHFGEISVRHVFQCARMKQIIWARDKNSEGEESADL
FLRGIGLREYSRYICFNFPFTHEQSLLSHLRFFPWDADVDKFKAWRQGRTGYPLVDA
GMRELWATGWMHNRIRVIVSSFAVKFLLLPWKWGMKYFWDTLLDADLECDILGW
QYISGSIPDGHELDRLDNPALQGAKYDPEGEYIRQWLPELARLPTEWIHHPWDAPLTV
LKASGVELGTNYAKPIVDIDTARELLAKAISRTREAQIMIGAA

CiBN
MNGAIGGDLLLNFPDMSVLERQRAHLKYLNPTFDSPLAGFFADSSMITGGEMDSYLS
TAGLNLPMMYGETTVEGDSRLSISPETTLGTGNFKKRKFDTETKDCNEKKKKMTMN
RDDLVEEGEEEKSKITEQNNGSTKSIKKMKHKAKKEENNFSNDSSKVTKELEKTDYI

ADAR2$_{DD}$
QLHLPQVLADAVSRLVLGKFGDLTDNFSSPHARRKVLAGVVMTTGTDVKDAKVISV
STGTKCINGEYMSDRGLALNDCHAEIISRRSLLRFLYTQLELYLNNKDDQKRSIFQKS
ERGGFRLKENVQFHLYISTSPCGDARIFSPHEPILEEPADRHPNRKARGQLRTKIESGQ
GTIPVRSNASIQTWDGVLQGERLLTMSCSDKIARWNVVGIQGSLLSIFVEPIYFSSIILG
SLYHGDHLSRAMYQRISNIEDLPPLYTLNKPLLSGISNAEARQPGKAPNFSVNWTVGD
SAIEVINATTGKDELGRASRLCKHALYCRWMRVHGKVPSHLLRSKITKPNVYHESKL
AAKEYQAAKARLFTAFIKAGLGAWVEKPTEQDQFSLT

Fluc (STOP 417)
ATGGAAGATGCCAAAAACATTAAGAAGGGCCCAGCGCCATTCTACCCACTCGAA
GACGGGACCGCCGGCGAGCAGCTGCACAAAGCCATGAAGCGCTACGCCCTGGTG
CCCGGCACCATCGCCTTTACCGACGCACATATCGAGGTGGACATTACCTACGCCG
AGTACTTCGAGATGAGCGTTCGGCTGGCAGAAGCTATGAAGCGCTATGGGCTGA
ATACAAACCATCGGATCGTGGTGTGCAGCGAGAATAGCTTGCAGTTCTTCATGCC
CGTGTTGGGTGCCCTGTTCATCGGTGTGGCTGTGGCCCCAGCTAACGACATCTAC
AACGAGCGCGAGCTGCTGAACAGCATGGGCATCAGCCAGCCCACCGTCGTATTC
GTGAGCAAGAAAGGGCTGCAAAAGATCCTCAACGTGCAAAAGAAGCTACCGATC
ATACAAAAGATCATCATCATGGATAGCAAGACCGACTACCAGGGCTTCCAAAGC
ATGTACACCTTCGTGACTTCCCATTTGCCACCCGGCTTCAACGAGTACGACTTCGT
GCCCGAGAGCTTCGACCGGGACAAAACCATCGCCCTGATCATGAACAGTAGTGG
CAGTACCGGATTGCCCAAGGGCGTAGCCCTACCGCACCGCACCGCTTGTGTCCGA
TTCAGTCATGCCCGCGACCCCATCTTCGGCAACCAGATCATCCCCGACACCGCTA
TCCTCAGCGTGGTGCCATTTCACCACGGCTTCGGCATGTTCACCACGCTGGGCTAC
TTGATCTGCGGCTTTCGGGTCGTGCTCATGTACCGCTTCGAGGAGGAGCTATTCTT
GCGCAGCTTGCAAGACTATAAGATTCAATCTGCCCTGCTGGTGCCCACACTATTT
AGCTTCTTCGCTAAGAGCACTCTCATCGACAAGTACGACCTAAGCAACTTGCACG
AGATCGCCAGCGGCGGGGCGCCGCTCAGCAAGGAGGTAGGTGAGGCCGTGGCCA
AACGCTTCCACCTACCAGGCATCCGCCAGGGCTACGGCCTGACAGAAACAACCA
GCGCCATTCTGATCACCCCCGAAGGGGACGACAAGCCTGGCGCAGTAGGCAAGG
TGGTGCCCTTCTTCGAGGCTAAGGTGGTGGACTTGGACACCGGTAAGACACTGGG
TGTGAACCAGCGCGGCGAGCTGTGCGTCCGTGGCCCCATGATCATGAGCGGCTAC
GTTAACAACCCCGAGGCTACAAACGCTCTCATCGACAAGGACGGCTAGCTGCAC
AGCGGCGACATCGCCTACTGGGACGAGGACGAGCACTTCTTCATCGTGGACCGGC
TGAAGAGCCTGATCAAATACAAGGGCTACCAGGTAGCCCCAGCCGAACTGGAGA
GCATCCTGCTGCAACACCCCAACATCTTCGACGCCGGGGTCGCCGGCCTGCCCGA
CGACGATGCCGGCGAGCTGCCCGCCGCAGTCGTCGTGCTGGAACACGGTAAAAC
CATGACCGAGAAGGAGATCGTGGACTATGTGGCCAGCCAGGTTACAACCGCCAA
GAAGCTGCGCGGTGGTGTTGTGTTCGTGGACGAGGTGCCTAAAGGACTGACCGGC
AAGTTGGACGCCCGCAAGATCCGCGAGATTCTCATTAAGGCCAAGAAGGGCGGC
AAGATCGCCGTGTAA

Uncropped scans of gel in Supplementary Fig.4g

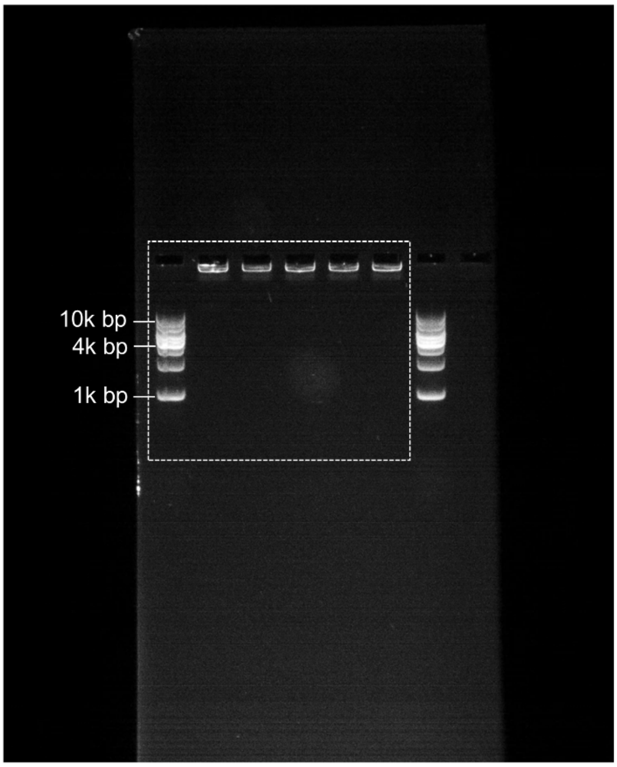

Supplement: Supplementary file 1 — Supplementary Information [file 41467_2026_72181_MOESM1_ESM.pdf]
