# Supplementary material for: Photoactivatable CRISPR/Cas13d via upconversion nanoparticles for deep tissue RNA engineering and orthopedic therapy
Source: Nat Commun. 2026 Apr 20;17:5216. doi: 10.1038/s41467-026-72181-6 (PMC13261002; doi:10.1038/s41467-026-72181-6)
Supplement: Supplementary file 2 — Reporting Summary [file 41467_2026_72181_MOESM2_ESM.pdf]

nature portfolio

Corresponding author(s): Xinlong Ma

Last updated by author(s): Mar 28, 2026

Reporting Summary

Nature Portfolio wishes to improve the reproducibility of the work that we publish. This form provides structure for consistency and transparency in reporting. For further information on Nature Portfolio policies, see our Editorial Policies and the Editorial Policy Checklist.

Statistics

For all statistical analyses, confirm that the following items are present in the figure legend, table legend, main text, or Methods section.

n/a	Confirmed	
☐	☒	The exact sample size (n) for each experimental group/condition, given as a discrete number and unit of measurement
☒	☐	A statement on whether measurements were taken from distinct samples or whether the same sample was measured repeatedly
☐	☒	The statistical test(s) used AND whether they are one- or two-sided<br>*Only common tests should be described solely by name; describe more complex techniques in the Methods section.*
☐	☒	A description of all covariates tested
☒	☐	A description of any assumptions or corrections, such as tests of normality and adjustment for multiple comparisons
☐	☒	A full description of the statistical parameters including central tendency (e.g. means) or other basic estimates (e.g. regression coefficient) AND variation (e.g. standard deviation) or associated estimates of uncertainty (e.g. confidence intervals)
☐	☒	For null hypothesis testing, the test statistic (e.g. F, t, r) with confidence intervals, effect sizes, degrees of freedom and P value noted<br>*Give P values as exact values whenever suitable.*
☒	☐	For Bayesian analysis, information on the choice of priors and Markov chain Monte Carlo settings
☒	☐	For hierarchical and complex designs, identification of the appropriate level for tests and full reporting of outcomes
☒	☐	Estimates of effect sizes (e.g. Cohen's d, Pearson's r), indicating how they were calculated

Our web collection on statistics for biologists contains articles on many of the points above.

Software and code

Policy information about availability of computer code

Data collection	Hydrodynamic size distribution and zeta potential were measured using a Zetasizer Nano ZS (Malvern Panalytical). Luminescence was measured using a GloMax Navigator Microplate Luminometer (Promega). CFX96 Real-Time System (Bio-Rad) is used for real-time PCR. Fluorescence image were obtained with Olympus IX83 confocal microscopy. Micro-CT scanning was subsequently performed using a high-resolution µCT system (SkyScan 1276, Bruker). In vivo bioluminescence was monitored using IVIS Spectrum system.
Data analysis	Protein structure was predicted using Alphafold2 and analyzed in PyMOL 2.5.2.<br>Data was analyzed in GraphPad Prism (Version 10.3.1). Imaging data was analyzed in ImageJ (v1.8.0).<br>In vivo data was analyzed in PerkinElmer Living Image Software 4.4.

For manuscripts utilizing custom algorithms or software that are central to the research but not yet described in published literature, software must be made available to editors and reviewers. We strongly encourage code deposition in a community repository (e.g. GitHub). See the Nature Portfolio guidelines for submitting code & software for further information.

Data

Policy information about availability of data

All manuscripts must include a data availability statement. This statement should provide the following information, where applicable:

- Accession codes, unique identifiers, or web links for publicly available datasets
- A description of any restrictions on data availability
- For clinical datasets or third party data, please ensure that the statement adheres to our policy

All data supporting the findings of this study are available within the paper and its Supplementary Information. The RNA sequencing data used in this study are deposited in the NCBI GEO database with accession number GSE325787. Source data are provided with this paper.

Research involving human participants, their data, or biological material

Policy information about studies with human participants or human data. See also policy information about sex, gender (identity/presentation), and sexual orientation and race, ethnicity and racism.

Reporting on sex and gender	Not available
Reporting on race, ethnicity, or other socially relevant groupings	Not available
Population characteristics	Not available
Recruitment	Not available
Ethics oversight	Not available

Note that full information on the approval of the study protocol must also be provided in the manuscript.

Field-specific reporting

Please select the one below that is the best fit for your research. If you are not sure, read the appropriate sections before making your selection.

[x] Life sciences [ ] Behavioural & social sciences [ ] Ecological, evolutionary & environmental sciences

For a reference copy of the document with all sections, see nature.com/documents/nr-reporting-summary-flat.pdf

Life sciences study design

All studies must disclose on these points even when the disclosure is negative.

Sample size	All in vitro experiments were performed with at least 3 independent replicates. All animal experiments were conducted with at least 5 animals per group.
Data exclusions	No data was excluded.
Replication	All experiments were conducted using a minimum of 3 independent replicates.
Randomization	Cells and mice were randomized to experimental groups.
Blinding	All groups were not blinded as sample preparation followed uniform protocols. This is not relevant to biological experiments and samples were not blinded.

Reporting for specific materials, systems and methods

We require information from authors about some types of materials, experimental systems and methods used in many studies. Here, indicate whether each material, system or method listed is relevant to your study. If you are not sure if a list item applies to your research, read the appropriate section before selecting a response.

Materials & experimental systems

n/a	Involved in the study
	[x] Antibodies
	[x] Eukaryotic cell lines
[x]	Palaeontology and archaeology
	[x] Animals and other organisms
[x]	Clinical data
[x]	Dual use research of concern
[x]	Plants

Methods

n/a	Involved in the study
[x]	ChIP-seq
[x]	Flow cytometry
[x]	MRI-based neuroimaging

Antibodies

Antibodies used: TET1 (1:500, MilliporeSigma #SAB2700730), TET2 (1:1000, ProteinTech #21207-1-AP), TET3 (1:500, Abcam #ab153724), PTEN (1:1000, CST #9188), phospho-Akt (Ser473) (1:1000, CST #4060), Akt (1:1000, CST #4691), Bcl-2 (1:1000, CST #15071), Bax (1:1000, Abcam #ab32503), cleaved Caspase-3 (1:1000, CST #9664), Histone H3 (1:2000, CST #9715), β-actin (1:10000, Millipore #A5441), Tubulin (1:5000, CST #2146), FLAG (1:200, MilliporeSigma #F1804) , HA (1:200, CST #3724), Alexa Fluor 488-conjugated anti-mouse IgG (1:500, Invitrogen #A-10680), Alexa Fluor 594-conjugated anti-rabbit IgG (1:500, Invitrogen #A-11012), 5hmC (1:10000, Active Motif #39769).

Validation: Antibodies were only chosen if there were validated references available.

Eukaryotic cell lines

Policy information about cell lines and Sex and Gender in Research

Cell line source(s): HEK293T cells were obtained from ATCC (CRL-11268). MLO-Y4 osteocyte-like cells were generously provided by Dr. Lynda Bonewald (University of Missouri-Kansas City).

Authentication: Cell lines are not authenticated due to their reliable sources.

Mycoplasma contamination: No mycoplasma contamination was detected in all cell lines.

Commonly misidentified lines (See ICLAC register): No commonly misidentified lines were used.

Animals and other research organisms

Policy information about studies involving animals; ARRIVE guidelines recommended for reporting animal research, and Sex and Gender in Research

Laboratory animals: Twelve-week-old male BALB/c mice were randomly divided into groups (n = 5 per group) and housed under standard specific pathogen-free conditions with a 12 h light/12 h dark cycle, an ambient temperature of 22 ± 2°C, and relative humidity of 50% – 60%, with free access to food and water.

Wild animals: There were no wild animals used in this study.

Reporting on sex: male mice

Field-collected samples: No field-collected samples were used in this study.

Ethics oversight: All animal experiments were conducted in accordance with Guidelines for Care and Use of Laboratory Animals and approved by the Institutional Animal Care and Use Committee of Tianjin Hospital. The approval number for this study is 2021YLS047.

Note that full information on the approval of the study protocol must also be provided in the manuscript.

Plants

Seed stocks	Not available
Novel plant genotypes	Not available
Authentication	Not available
