# Supplementary material for: Photoactivatable CRISPR/Cas13d via upconversion nanoparticles for deep tissue RNA engineering and orthopedic therapy
Source: Nat Commun. 2026 Apr 20;17:5216. doi: 10.1038/s41467-026-72181-6 (PMC13261002; doi:10.1038/s41467-026-72181-6)
Supplement: Supplementary file 3 — Transparent Peer Review file [file 41467_2026_72181_MOESM3_ESM.pdf]

nature portfolio

Peer Review File

Photoactivatable CRISPR/Cas13d via upconversion nanoparticles for deep tissue RNA engineering and orthopedic therapy

Corresponding Author: Professor Xinlong Ma

This file contains all reviewer reports in order by version, followed by all author rebuttals in order by version.

Version 0:

Reviewer comments:

Reviewer #1

(Remarks to the Author)
This paper presents an ambitious approach to achieving spatiotemporal control of RNA editing via near-infrared irradiation using UCNPs-PEI@paCas13d. However, as listed below, numerous concerns and unresolved issues remain (many of which are fundamental matters that should be addressed before conducting biological experiments). As such, I cannot recommend publication of this paper in its current form.

1. The paper claims that “470 nm light irradiation causes CRY2PHR to undergo a rapid structural change and form a dimer with CIBN.” However, the absorption spectrum of CRY2PHR is not shown, and there is no direct evidence of dimer formation under blue light irradiation.

2. Why does CRY2PHR, which shows no affinity for CIBN prior to blue light irradiation, exhibit affinity for CIBN after undergoing structural changes due to blue light irradiation? Furthermore, while it is stated that the structural change in CRY2PHR upon blue light irradiation is reversible, what is the evidence for this? What is the stability of the dimer after formation?

3. There is absolutely no detailed information on the UCNPs synthesis method or what kind of UCNPs were obtained. Furthermore, since as-synthesized UCNPs are typically hydrophobic, a proper explanation is needed on how surface modification with the water-soluble polymer PEI was achieved. Furthermore, if the as-synthesized UCNPs are already water dispersible, the synthesis method and the nature of the surface ligands become a point of concern. Additionally, information is needed on the solvents used for the DLS measurements (Fig. 2b) and zeta potential measurements (Fig. S3a) of both UCNPs and UCNPs-PEI. Why are the UCNPs negatively charged?

4. The statement “The XRD pattern confirmed the formation of core-shell structured UCNPs-PEI (Fig. 2d)” is entirely incorrect.

5.The statement “Under 980 nm laser excitation, UCNPs-PEI exhibited a sharp emission peak at approximately 470 nm (blue light) (Fig. 2f)” should be supplemented. To demonstrate how PEI surface modification affects wavelength conversion efficiency, the emission spectra of both UCNPs and UCNPs-PEI (before and after PEI surface modification) should be presented, and the emission intensities compared.

6. It is stated that “UCNPs-PEI exhibited good biocompatibility (Figure S3c).” However, PEI is known to be highly cytotoxic. Why does UCNPs-PEI not show cytotoxicity even at a high concentration of 1 mg/mL?

7. It is stated that “this NIR-induced editing efficiency was equivalent to that achieved by direct blue light activation of the padCas13 system.” However, the intensity of blue light emitted directly from a blue light source should be vastly different from that emitted from NIR-excited UCNPs. Why, then, is the editing efficiency nearly equivalent?

8. UCNPs-PEI@paCas13d was administered to cells and mice to investigate therapeutic effects. How was UCNPs-PEI@paCas13d synthesized in the first place? If paCas13d is merely electrostatically adsorbed onto UCNPs-PEI, questions

remain about its stability within cells and in the bloodstream. In addition, what pathway is used for UCNPs-PEI@paCas13d uptake into cells? If it is taken up via the endocytosis pathway, how can CRY2PHR and CIBN be delivered to the cell nucleus? If UCNPs-PEI@paCas13d is directly transfected into the cytoplasm, why do CRY2PHR and CIBN detach from UCNPs-PEI? Does xenophagy occur before this?

9. How is UCNPs-PEI metabolized after remaining in the body?

Reviewer #2

(Remarks to the Author)
The research group developed a novel photoactivatable CRISPR/Cas13d (paCas13d) system for precise, non-invasive RNA targeting in deep tissues. Furthermore, they engineered upconversion nanoparticles (UCNPs-PEI) to convert near-infrared light to blue light, enabling activation in bone tissue. In an in vivo mouse model of steroid-induced osteonecrosis, this system effectively knocked down TET3, preventing bone loss while preserving glucocorticoid function. This reported platform seems to offers spatiotemporal RNA control, combining CRISPR programmability with optical precision for localized therapeutic interventions with minimal systemic effects. Nevertheless, the chosen experimental design setup doesn't demonstrate the clinical need to such advanced system, most due to the omission of conventional controls.

Figure 1
The authors demonstrate photo-spatiotemporal control of the paCas13d system under blue light (470 nm). However, to confirm the specificity of the system, it is important to also show its lack of responsiveness to near-infrared (NIR) light under the same experimental conditions. This would strengthen the claim that the system is selectively activated by blue light and not by NIR, which is used elsewhere in the manuscript.

Fig.1F & table S1.
The manuscript does not specify which in silico CRISPR gRNA design platform was used to generate the gRNA sequences. Additionally, there is no information provided on the predicted knockdown (KD) efficiency of the designed gRNAs. Including these details is essential for evaluating the rationale behind the selection of gRNA sequences and their expected performance.

Fig.2b
Please include the polydispersity index (PDI) of UCNPs-PEI both before and after loading with the paCas13d system. Also, report the change in particle size upon loading. These parameters are important for assessing the stability and uniformity of the nanoparticle formulation.

Fig. S3c What was the loading efficiency of the paCas13d system into UCNPs-PEI? This efficiency is important in order to understand the dosage used in vitro and in vivo.

Nanoparticle Uptake Mechanism
The authors should describe the proposed cellular uptake mechanism of UCNPs-PEI. Clarifying this would help readers understand how the delivery system interacts with target cells and contributes to therapeutic efficacy.

In-vivo study (figure 5)

It is unclear why a siRNA control group was not included to benchmark the therapeutic efficacy of the paCas13d system. The authors should address this omission in the limitations section.
The manuscript does not include a blue light control group to demonstrate the necessity of using UCNPs-PEI for NIR-to-blue light phototransduction. Without this comparison, it is difficult to assess whether the UCNPs-PEI system offers any advantage over direct blue light activation usage in vivo with paCAS13d system alone. This should be addresseddemonstrated experimentally, for example, using ex vivo tissue explant system.

Did the authors assess the in vivo bioavailability of UCNPs-PEI@paCas13d? If not, what rationale supports the decision to administer weekly intrafemoral injections? Clarifying this would help justify the dosing strategy used in the study.

The authors should explain why intrafemoral injection was performed and necessary versus a systemic injection. How does a localized anatomical delivery pose an advantage versus conventional localized delivery of a siRNA system? Could this be practically used in a clinical setting? This is important since the manuscript tittle implies a new therapy.

Version 1:

Reviewer comments:

Reviewer #1

(Remarks to the Author)
The authors' response to my previous comments/questions was largely satisfactory. However, two new significant concerns have arisen based on the newly added experimental data, as outlined below. Therefore, unfortunately, I cannot recommend

publication of the paper as it stands. A proper explanation based on solid experimental evidence is required for these two points of concern.

(1) Figure R1 (Supplementary Fig. 1) demonstrates that CRY2PHR-mCherry rapidly accumulates in the nucleus within one minute after 470 nm light exposure, via heterodimerisation with the nuclear-localised protein CIBN. Conversely, upon cessation of light exposure, nuclear CRY2PHR-mCherry gradually decreased over 30 minutes, returning to baseline levels of cytoplasmic distribution. While these results indirectly demonstrate CRY2PHR-mCherry heterodimerisation with CIBN, they raise new questions. Specifically, the following three points:

(i) If the CRY2PHR/CIBN heterodimer transported into the nucleus gradually dissociates in the dark, why does CRY2PHR-mCherry not persist in the nucleus?
(ii) If dissociation does not occur, does the heterodimer persist indefinitely within the nucleus?
(iii) By what mechanism is CRY2PHR-mCherry or the CRY2PHR/CIBN heterodimer expelled from the nucleus?

There is no explanation based on experimental evidence for these points, and it cannot be asserted that reversibility has been demonstrated.

(2) Based on my previous comments, the authors have now presented the emission spectra of UCNPs-PEI [Fig. R2a (Fig. 2g)] and ligand-free UCNPs [Fig. R2b (Supplementary Fig. 4a)]. They assert that, "As shown in Figure R2a and R2b, both samples exhibit a dominant blue emission band centered at ≈470 nm, consistent with Tm^{3+}-sensitized upconversion, indicating that PEI surface modification does not change the emission wavelength profile." However, the emission spectra have clearly changed; the emission peaks observed in the UV region and around 450 nm for UCNPs are completely absent in UCNPs-PEI. An explanation for this reason is required.

Reviewer #2

(Remarks to the Author)
The authors have adequately addressed all concerns raised. They have performed additional experiments and text alterations that bring added clarity to the developed system and its purported mechanism of action and future therapeutic application.
I have no further questions

Version 2:

Reviewer comments:

Reviewer #1

(Remarks to the Author)
The authors have provided appropriate responses and revisions to my comments and questions during the second round of peer review. Therefore, I recommend this paper for publication in your journal in its current state.

Open Access This Peer Review File is licensed under a Creative Commons Attribution 4.0 International License, which permits use, sharing, adaptation, distribution and reproduction in any medium or format, as long as you give appropriate credit to the original author(s) and the source, provide a link to the Creative Commons license, and indicate if changes were made.
In cases where reviewers are anonymous, credit should be given to 'Anonymous Referee' and the source.
The images or other third party material in this Peer Review File are included in the article's Creative Commons license, unless indicated otherwise in a credit line to the material. If material is not included in the article's Creative Commons license and your intended use is not permitted by statutory regulation or exceeds the permitted use, you will need to obtain permission directly from the copyright holder.
To view a copy of this license, visit https://creativecommons.org/licenses/by/4.0/

Our Replies to the Reviewer's Comments and the Revisions Made to the Manuscript

Reviewer #1 (Remarks to the Author):

This paper presents an ambitious approach to achieving spatiotemporal control of RNA editing via near-infrared irradiation using UCNPs-PEI@paCas13d. However, as listed below, numerous concerns and unresolved issues remain (many of which are fundamental matters that should be addressed before conducting biological experiments). As such, I cannot recommend publication of this paper in its current form.

1. The paper claims that "470 nm light irradiation causes CRY2PHR to undergo a rapid structural change and form a dimer with CIBN." However, the absorption spectrum of CRY2PHR is not shown, and there is no direct evidence of dimer formation under blue light irradiation.

Response: We thank the reviewer for raising these important points regarding the absorption spectrum and direct evidence of dimer formation.

Regarding the absorption spectrum: The FAD chromophore within CRY2PHR exhibits well-characterized absorption in the blue light region (450-500 nm), as extensively documented in the optogenetics literature.[1, 2] To functionally validate this spectral characteristic in our system, we performed nucleus-cytoplasm translocation assays testing multiple wavelengths (Fig. R1). These experiments demonstrate that only 470 nm blue light induced rapid nuclear accumulation of CRY2PHR-mCherry within 1 minute through heterodimerization with nuclear-localized CIBN, while other tested wavelengths (365, 530, 635, 730 nm) outside the blue light absorption range elicited no response. This wavelength-specificity profile provides functional confirmation of CRY2PHR's blue light-selective absorption and directly validates the spectral characteristics reported in the literature.

Regarding direct evidence of dimer formation: The nucleus-cytoplasm translocation assays provide direct functional evidence for light-induced CRY2PHR/CIBN heterodimerization. Upon 470 nm illumination, cytoplasmic CRY2PHR-mCherry rapidly translocated to the nucleus within 1 minute by binding to nuclear-localized NLS-GFP-CIBN-NLS, demonstrating light-dependent association. Upon light cessation, nuclear CRY2PHR-mCherry gradually dissipated over 30 minutes, returning to baseline cytoplasmic distribution, demonstrating spontaneous dissociation with kinetics consistent with published parameters.[1, 2] Dark controls showed negligible spontaneous translocation throughout the observation period. These experiments directly demonstrate that CRY2PHR/CIBN heterodimerization occurs specifically and reversibly under blue light control, confirming the reliable operation of this well-

characterized optogenetic system in our split-Cas13d application.

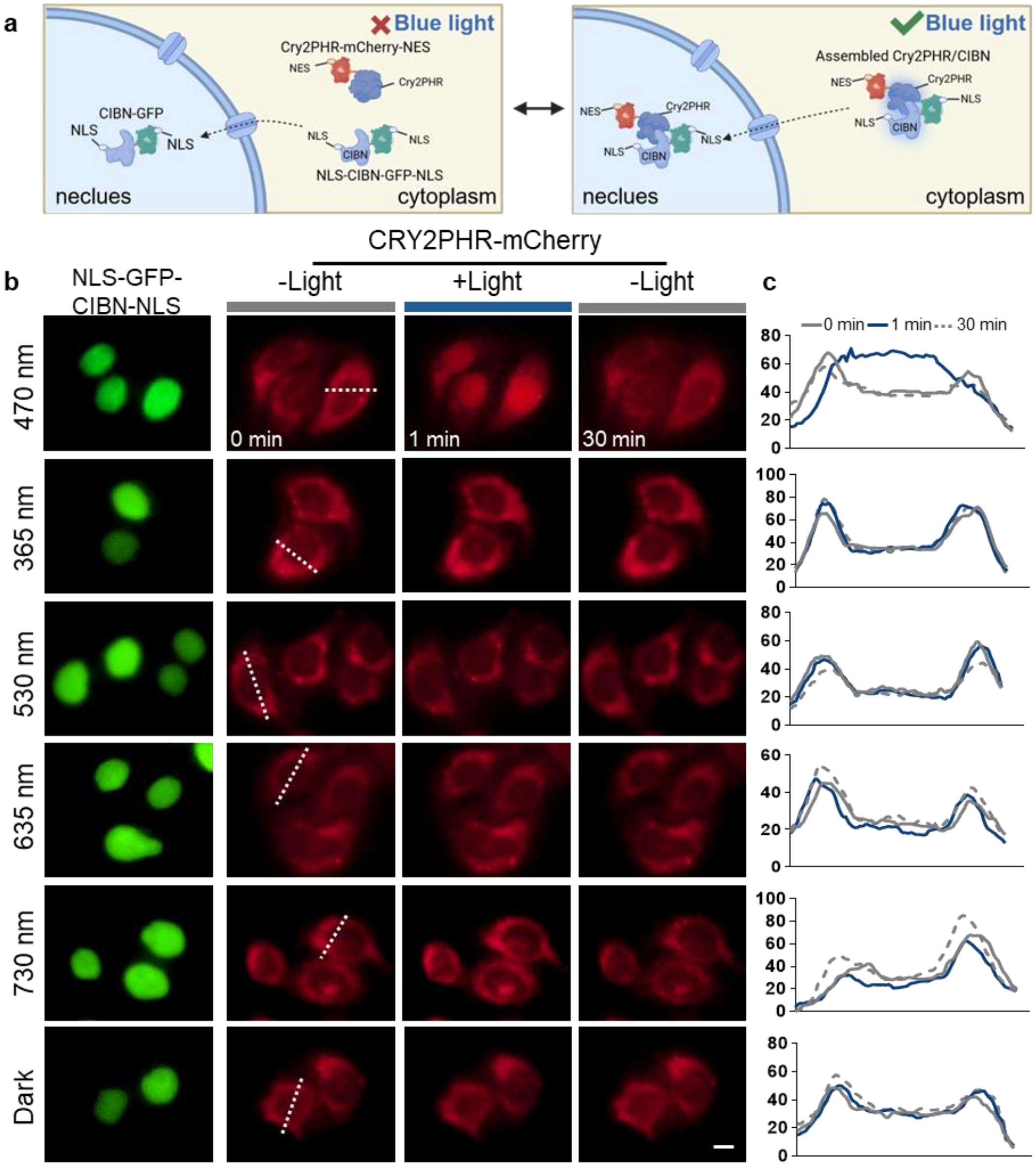

Fig. R1 Wavelength specificity and reversibility of CRY2PHR/CIBN light-inducible dimerization

(a) Schematic illustration of the nucleus-cytoplasm translocation assay. Nuclear-localized NLS-GFP-CIBN-NLS (green) and cytoplasmic CRY2PHR-mCherry-NES (red) undergo heterodimerization upon blue light (470 nm) illumination, inducing CRY2PHR-mCherry nuclear translocation. NLS: nuclear localization signal; NES: nuclear export signal. **(b)** Representative live-cell imaging of HEK293T cells expressing NLS-GFP-CIBN-NLS and CRY2PHR-mCherry-NES exposed to different wavelengths (470 nm, 365 nm, 530 nm, 635 nm, 730 nm) or maintained in darkness (Dark). Left column: NLS-GFP-CIBN-NLS nuclear localization. Middle columns: CRY2PHR-mCherry distribution before illumination (−Light, 0 min), 1 minute after light onset (+Light, 1 min), and 30 minutes after light cessation (−Light, 30 min). Scale

bar, 10 μm. **(c)** Quantitative line scan analysis of CRY2PHR-mCherry fluorescence intensity over time for each wavelength condition shown in **(b)**.

Changed in the “Revised manuscript” (Lines 122-146):

Then we implemented the well-characterized CRY2PHR/CIBN blue light-inducible dimerization system to control split Cas13d reconstitution (Fig. 1c). The molecular mechanism underlying this optogenetic switch has been extensively established: the CRY2PHR photolyase homology region contains a flavin adenine dinucleotide (FAD) chromophore that undergoes photoreduction upon blue light absorption, triggering a conformational change from a closed, CIBN-inaccessible state to an open conformation that exposes the CIBN-binding interface and enables high-affinity heterodimer formation (Kd ~1 μM).[1, 2] Upon light withdrawal, the FAD chromophore spontaneously re-oxidizes in the dark, reverting CRY2PHR to its closed conformation and causing dimer dissociation. This reversible photocycle enables light-gated spatial approximation of split Cas13d fragments, restoring catalytic activity exclusively under illumination while maintaining inactive separation in darkness. To directly validate the functional operation of this light-dependent interaction in our system, we performed nucleus-cytoplasm translocation assays using nuclear-localized NLS-GFP-CIBN-NLS and cytoplasmic CRY2PHR-mCherry (Supplementary Fig. 1a). Upon 470 nm illumination, CRY2PHR-mCherry rapidly accumulated in the nucleus within 1 minute through heterodimerization with nuclear-localized CIBN, confirming robust light-induced association. Critically, other wavelengths (365, 530, 635, 730 nm) outside the blue light absorption range elicited no response, demonstrating strict wavelength specificity consistent with the FAD chromophore absorption profile. Upon light cessation, nuclear CRY2PHR-mCherry gradually decreased over 30 minutes, returning to baseline cytoplasmic distribution, directly demonstrating complete reversibility with a dissociation half-life consistent with published kinetic parameters (Supplementary Fig. 1b, c).[1, 2] Dark controls showed negligible spontaneous translocation throughout the observation period. These functional validation experiments confirm that our paCas13d system operates through the well-established CRY2PHR/CIBN photocycle, providing wavelength-specific, and reversible optogenetic control over Cas13d activity.

Changed in the “Supplementary Information” (Supplementary Fig. 1)

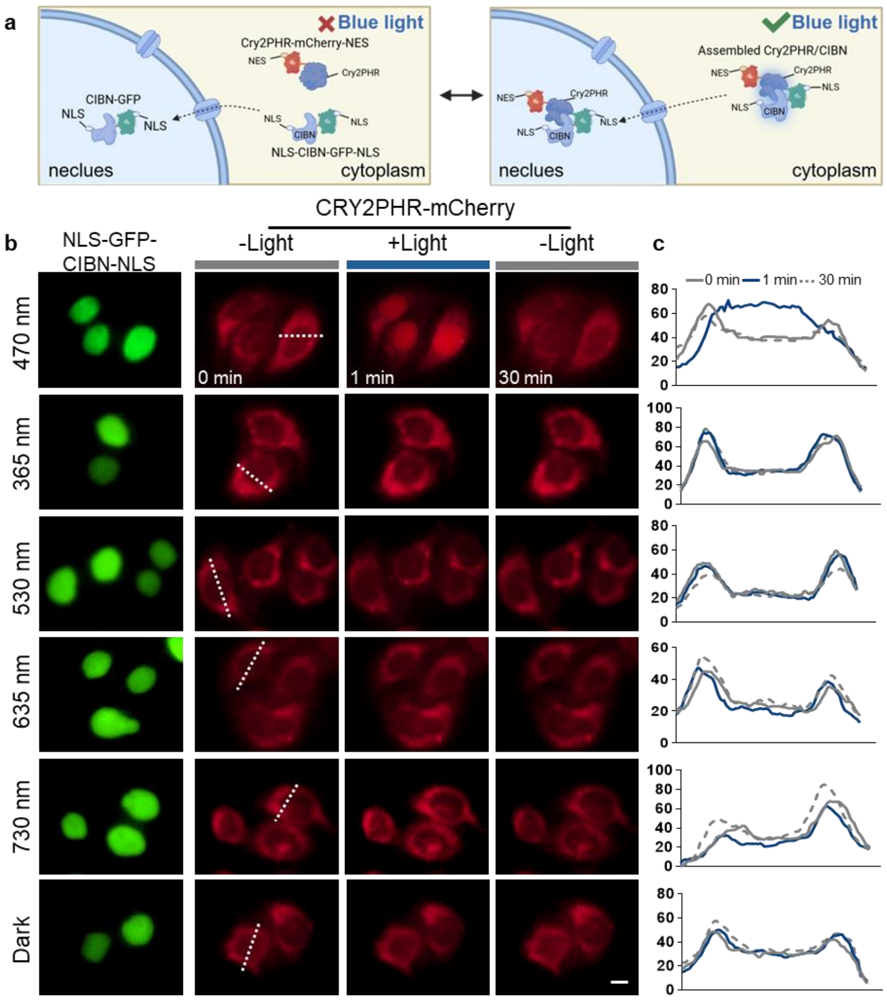

Supplementary Fig. 1 Wavelength specificity and reversibility of CRY2PHR/ CIBN light-inducible dimerization

(a) Schematic illustration of the nucleus-cytoplasm translocation assay. Nuclear-localized NLS-GFP-CIBN-NLS (green) and cytoplasmic CRY2PHR-mCherry-NES (red) undergo heterodimerization upon blue light (470 nm) illumination, inducing CRY2PHR-mCherry nuclear translocation. NLS: nuclear localization signal; NES: nuclear export signal. **(b)** Representative live-cell imaging of HEK293T cells expressing NLS-GFP-CIBN-NLS and CRY2PHR-mCherry-NES exposed to different wavelengths (470 nm, 365 nm, 530 nm, 635 nm, 730 nm) or maintained in darkness (Dark). Left column: NLS-GFP-CIBN-NLS nuclear localization. Middle columns: CRY2PHR-mCherry distribution before illumination (−Light, 0 min), 1 minute after light onset (+Light, 1 min), and 30 minutes after light cessation (−Light, 30 min). Scale bar, 10 μm. **(c)** Quantitative line scan analysis of CRY2PHR-mCherry fluorescence intensity over time for each wavelength condition shown in **(b)**.

2. Why does CRY2PHR, which shows no affinity for CIBN prior to blue light irradiation, exhibit affinity for CIBN after undergoing structural changes due to blue light irradiation? Furthermore, while it is stated that the structural change in CRY2PHR upon blue light irradiation is reversible, what is the evidence for this? What is the stability of the dimer after formation?

Response: We thank the reviewer for these mechanistic questions regarding the molecular basis of light-dependent CRY2PHR/CIBN interaction.

Why does CRY2PHR show no affinity for CIBN before blue light but exhibits high affinity after illumination? The light-dependent affinity switch arises from a FAD-mediated conformational change that has been extensively characterized in the cryptochrome literature.[1, 2] In the dark-adapted state, the FAD chromophore within CRY2PHR exists in an oxidized form, maintaining CRY2PHR in a closed, compact conformation that sterically occludes the CIBN-binding interface. Upon absorption of blue light (~470 nm), the FAD undergoes photoreduction, which destabilizes the closed conformation and triggers a conformational rearrangement. This light-induced opening exposes a hydrophobic binding surface that exhibits high affinity for CIBN (Kd ~1 μM), enabling heterodimerization. The dark-state closed conformation shows negligible CIBN binding because the interaction interface remains inaccessible.

What is the evidence for reversibility of the structural change? Our nucleus-cytoplasm translocation experiments provide direct functional evidence for complete reversibility (Fig. R1). Using the established translocation assay approach for validating inducible dimerization systems,[3] we designed nuclear-localized NLS-GFP-CIBN-NLS and cytoplasmic CRY2PHR-mCherry-NES to visualize the light-dependent association-dissociation cycle. Upon 470 nm illumination, CRY2PHR-mCherry rapidly accumulated in the nucleus within 1 minute, demonstrating light-induced heterodimerization that drives nuclear translocation through binding to nuclear-localized CIBN. Critically, upon light cessation, nuclear CRY2PHR-mCherry gradually decreased over 30 minutes, returning to baseline cytoplasmic distribution, directly demonstrating complete reversibility with a dissociation half-life consistent with published kinetic parameters.[1, 2]

What is the stability of the dimer after formation? Our translocation experiments demonstrate that dimer stability is strictly light-dependent. During 470 nm illumination, CRY2PHR-mCherry remained stably localized in the nucleus for the duration of light exposure, indicating that the heterodimer is stable as long as the FAD remains in the photoreduced state. However, once illumination ceases, the dimer spontaneously dissociates with a half-life of approximately 10-15 minutes as FAD re-oxidizes and CRY2PHR reverts to its closed conformation, consistent with published kinetic

parameters.[2] These functional data confirm that our paCas13d system operates through the well-characterized, reversible CRY2PHR/CIBN photocycle.

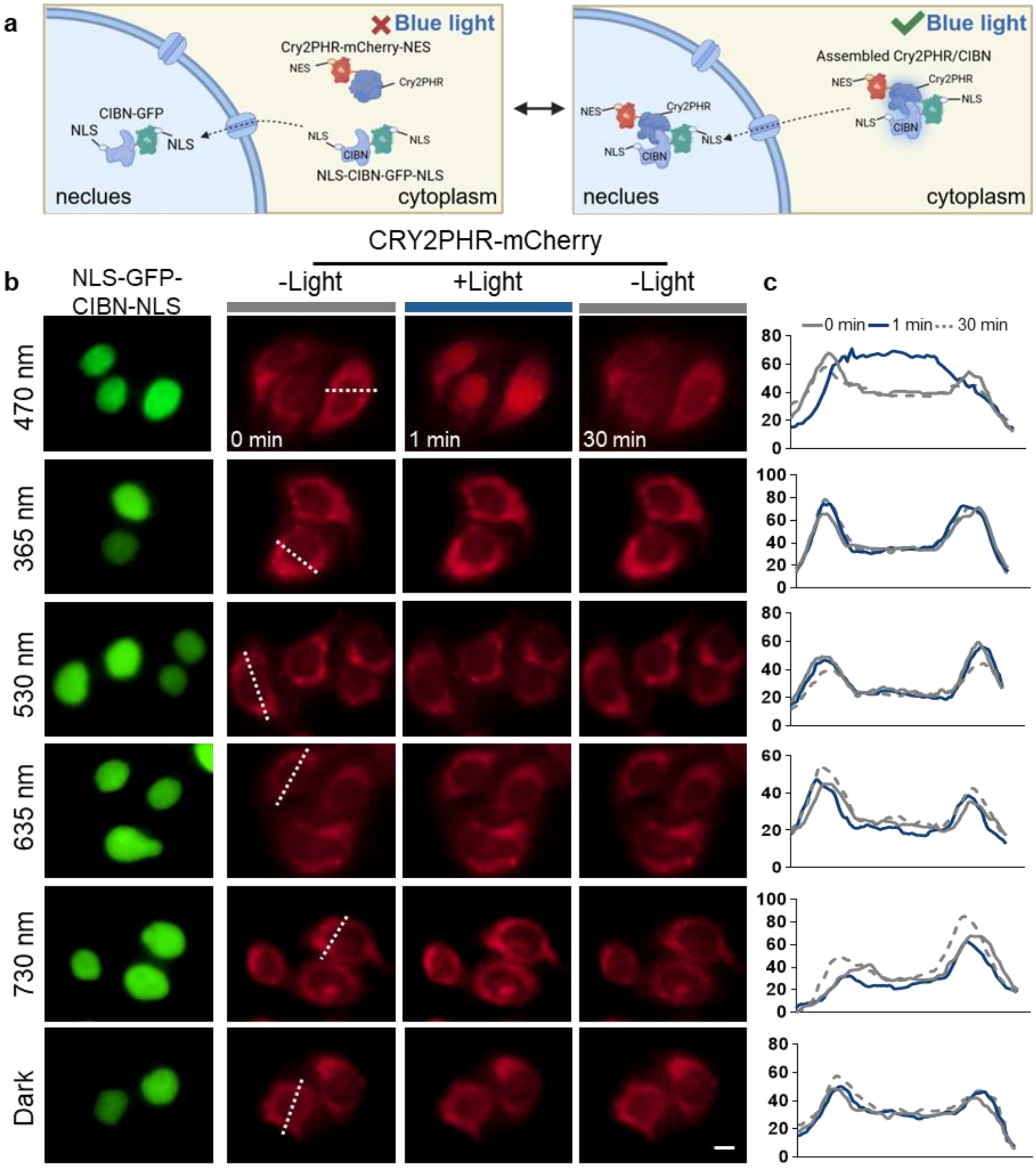

Fig. R1 Wavelength specificity and reversibility of CRY2PHR/CIBN light-inducible dimerization

(a) Schematic illustration of the nucleus-cytoplasm translocation assay. Nuclear-localized NLS-GFP-CIBN-NLS (green) and cytoplasmic CRY2PHR-mCherry-NES (red) undergo heterodimerization upon blue light (470 nm) illumination, inducing CRY2PHR-mCherry nuclear translocation. NLS: nuclear localization signal; NES: nuclear export signal. **(b)** Representative live-cell imaging of HEK293T cells expressing NLS-GFP-CIBN-NLS and CRY2PHR-mCherry-NES exposed to different wavelengths (470 nm, 365 nm, 530 nm, 635 nm, 730 nm) or maintained in darkness (Dark). Left column: NLS-GFP-CIBN-NLS nuclear localization. Middle columns: CRY2PHR-mCherry distribution before illumination (−Light, 0 min), 1 minute after light onset (+Light, 1 min), and 30 minutes after light cessation (−Light, 30 min). Scale

bar, 10 μm. **(c)** Quantitative line scan analysis of CRY2PHR-mCherry fluorescence intensity over time for each wavelength condition shown in (b).

Changed in the "Revised manuscript" (Lines 122-146):

Then we implemented the well-characterized CRY2PHR/CIBN blue light-inducible dimerization system to control split Cas13d reconstitution (Fig. 1c). The molecular mechanism underlying this optogenetic switch has been extensively established: the CRY2PHR photolyase homology region contains a flavin adenine dinucleotide (FAD) chromophore that undergoes photoreduction upon blue light absorption, triggering a conformational change from a closed, CIBN-inaccessible state to an open conformation that exposes the CIBN-binding interface and enables high-affinity heterodimer formation (Kd ~1 μM).[1, 2] Upon light withdrawal, the FAD chromophore spontaneously re-oxidizes in the dark, reverting CRY2PHR to its closed conformation and causing dimer dissociation. This reversible photocycle enables light-gated spatial approximation of split Cas13d fragments, restoring catalytic activity exclusively under illumination while maintaining inactive separation in darkness. To directly validate the functional operation of this light-dependent interaction in our system, we performed nucleus-cytoplasm translocation assays using nuclear-localized NLS-GFP-CIBN-NLS and cytoplasmic CRY2PHR-mCherry (Supplementary Fig. 1a). Upon 470 nm illumination, CRY2PHR-mCherry rapidly accumulated in the nucleus within 1 minute through heterodimerization with nuclear-localized CIBN, confirming robust light-induced association. Critically, other wavelengths (365, 530, 635, 730 nm) outside the blue light absorption range elicited no response, demonstrating strict wavelength specificity consistent with the FAD chromophore absorption profile. Upon light cessation, nuclear CRY2PHR-mCherry gradually decreased over 30 minutes, returning to baseline cytoplasmic distribution, directly demonstrating complete reversibility with a dissociation half-life consistent with published kinetic parameters (Supplementary Fig. 1b, c).[1, 2] Dark controls showed negligible spontaneous translocation throughout the observation period. These functional validation experiments confirm that our paCas13d system operates through the well-established CRY2PHR/CIBN photocycle, providing wavelength-specific, and reversible optogenetic control over Cas13d activity.

3. There is absolutely no detailed information on the UCNPs synthesis method or what kind of UCNPs were obtained. Furthermore, since as-synthesized UCNPs are typically hydrophobic, a proper explanation is needed on how surface modification with the water-soluble polymer PEI was achieved. Furthermore, if the as-synthesized UCNPs are already water dispersible, the synthesis method and the nature of the surface ligands become a point of concern. Additionally, information is needed on the solvents used for the DLS measurements (Fig. 2b) and zeta potential measurements (Fig. S3a) of both UCNPs and UCNPs-PEI. Why are the UCNPs negatively charged?

Response: Thank you for bringing this important concern to our attention. We apologize for not providing sufficient experimental details of the UCNPs synthesis and surface modification procedures in the initial submission. In the revised manuscript, we have now substantially expanded the UCNPs synthesis and characterization section, clearly specified the type/composition of UCNPs, and provided a detailed description of the PEI functionalization process.

In the revised manuscript, we now explicitly state that the blue-emitting UCNPs core is $NaYF_4$:20%Yb/0.5%Tm, synthesized via a thermal decomposition route, followed by epitaxial growth of an optically inert $NaYF_4$ shell to mitigate surface quenching (core-shell UCNPs). The full precursor amounts, temperatures, reaction times, purification steps, and dispersion solvent have been provided in the Experimental Section.

In our work, the as-synthesized core-shell UCNPs are oleate-capped and dispersed in cyclohexane (hydrophobic). They become water-dispersible only after ligand removal, and PEI coating is then performed on the ligand-free UCNPs. Specifically, we employed a two-step ligand-exchange strategy to obtain the hydrophilic UCNPs-PEI for further biological applications. The details have been provided in the Experimental Section.

In the revised manuscript, we clarify that DLS for both ligand-free UCNPs and UCNPs-PEI was measured in H_2O, and the zeta potentials of both nanoparticles were measured in PBS (pH 7.4). Under physiological pH, the negative zeta potential observed for ligand-free UCNPs is reasonable and has been reported previously. In particular, Bogdan *et al.* prepared ligand-free, water-dispersible lanthanide-doped UCNPs and investigated their surface charge as a function of pH, showing that ligand-free UCNPs can exhibit strongly negative zeta potentials at near-neutral pH.[4] This behavior is consistent with the formation of negatively charged surface species (e.g., deprotonated surface hydroxyls/coordination of surface rare earth sites with OH^- in aqueous media), which provides electrostatic stabilization at biologically relevant pH. However, after

PEI coating, the surface potential shifts toward positive values due to the amine-rich polymer layer, consistent with successful PEI functionalization.

Changed in the "Revised manuscript" (Lines 730-770):

Upconversion Nanoparticle Synthesis and Characterization: The blue-emitting UCNPs core ($NaYF_4$:20%Yb/0.5%Tm) were synthesized via a thermal decomposition method.[5, 6] Lanthanide precursors (0.795 mmol $Y(CH_3CO_2)_3$, 0.2 mmol $Yb(CH_3CO_2)_3$, 0.005 mmol $Tm(CH_3CO_2)_3$) were mixed with 6 mL oleic acid and 15 mL 1-octadecene, and then the mixture was slowly heated to 110 °C under vacuum with magnetic stirring for 1 h. Subsequently, the temperature of the mixed solution was increased to 160 ° C and maintained for 45 min under an argon atmosphere. After cooling to 50 °C, a methanol solution (8 mL) containing 2.5 mmol NaOH and 4 mmol NH_4F was added, followed by stirring for 30 min. Then, the reaction was heated to 110 ℃ under vacuum for 10 min to eliminate methanol, followed by heating to 300 ℃ and maintaining for 1.5 h under an argon atmosphere. UCNPs were purified by ethanol precipitation and centrifugation (6,000 rpm, 10 min), then redispersed in cyclohexane for further use.

To reduce surface - related quenching and preserve emission intensity, an inert $NaYF_4$ shell was epitaxially deposited on the as-prepared UCNPs core. In brief, 0.4 mmol $Y(CH_3CO_2)_3$ was combined with oleic acid (6 mL) and 1-octadecene (15 mL), degassed at 110 °C for 1 h, and then heated to 160 °C for 45 min under an inert atmosphere to obtain a clear, uniform solution. After the mixture was cooled to 50 °C, UCNP cores (1 mmol, dispersed in 4 mL cyclohexane) were added. The suspension was vacuum-treated at 110 °C for 20 min to remove cyclohexane. A methanolic precursor solution (6 mL) containing NaOH (1 mmol) and NH_4F (1.6 mmol) was then introduced at 45 °C, followed by stirring for 30 min. Methanol was subsequently removed under vacuum at 110 °C for another 20 min. The reaction temperature was ramped to 300 °C under inert gas and maintained for 1.5 h. After cooling to room temperature, the core-shell UCNPs were collected, washed three times with ethanol, and finally redispersed in 4 mL cyclohexane for subsequent use.

The surface modification of polyethylenimine (PEI) was carried out with a two-step ligand-exchange method.[7] The hydrophobic UCNPs (4 mL cyclohexane dispersion) were precipitated with ethanol and redispersed in 20 mL HCl solution (0.1 M). The mixture was sonicated at 45 °C for 1 h to remove oleate ligands, then the ligand-free UCNPs were collected by centrifugation and redispersed in H_2O (1 mL). Subsequently, the PEI (50 mg, Mw ~25,000) was dissolved in 9 mL deionized water, and the pH was adjusted to around 11 using 0.1 M NaOH. Ligand-free UCNPs (1 mL) were then added dropwise under continuous stirring and allowed to react for 2 h. The resulting dispersion was mixed with 10 mL diethylene glycol (DEG) and heated at 105 °C for 1 h, followed

by transfer into a Teflon-lined autoclave and incubation at 160 °C for 2 h. The UCNPs-PEI were harvested by centrifugation, sequentially washed with ethanol and deionized water, and finally stored in water at 4 ℃ for further use.

Dynamic Light Scattering (DLS) and Zeta Potential Analysis: Hydrodynamic size distribution and zeta potential of ligand-free UCNPs and UCNPs-PEI were measured using a Zetasizer Nano ZS (Malvern Panalytical) at 25 °C. For DLS, samples were dispersed in ultrapure water (0.1 mg/mL) and sonicated for 5 min prior to measurement. For zeta potential measurements, samples were dispersed in PBS (pH 7.4) at the same concentration (0.1 mg/mL) and sonicated for 5 min before measurement. Each sample was analyzed in triplicate.

4. The statement "The XRD pattern confirmed the formation of core-shell structured UCNPs-PEI (Fig. 2d)" is entirely incorrect.

Response: Thank you very much for pointing this out, and we apologize for the inaccurate wording in the original submission. We fully agree that XRD alone cannot confirm a core-shell architecture, and it also cannot verify the presence of a PEI coating (PEI is amorphous and does not produce characteristic diffraction peaks). Moreover, because both the core and the inert shell are based on $NaYF_4$, the diffraction pattern is expected to be dominated by the $NaYF_4$ lattice and is generally not sufficient to distinguish core-only from core-shell structures.

Accordingly, we have revised the manuscript to use revised Fig. 2c (previous Fig. 2d) only to demonstrate the crystalline phase and phase purity of the core-shell UCNPs after surface processing, i.e., the diffraction peaks match well with the standard hexagonal β-$NaYF_4$ pattern, and no detectable impurity peaks are observed. The evidence for PEI functionalization is now supported by the increase in hydrodynamic size and the zeta potential shift measured in PBS (pH 7.4), rather than by XRD.

Changed in the "Revised manuscript" (Lines 242-245):

The X-ray diffraction (XRD) pattern of the UCNPs-PEI after shell growth and subsequent ligand modification matches well with the standard hexagonal β-$NaYF_4$ phase with no detectable impurity peaks, indicating that the crystalline host lattice is preserved (Fig. 2c).

5. The statement "Under 980 nm laser excitation, UCNPs-PEI exhibited a sharp emission peak at approximately 470 nm (blue light) (Fig. 2f)" should be supplemented. To demonstrate how PEI surface modification affects wavelength conversion efficiency, the emission spectra of both UCNPs and UCNPs-PEI (before and after PEI surface modification) should be presented, and the emission intensities compared.

Response: Thanks a lot for this constructive suggestion, and we apologize that the original submission did not include the "before/after PEI" spectral comparison. As you suggested, we have now added the upconversion emission spectra of ligand-free UCNPs (before PEI modification) and UCNPs-PEI (after PEI modification) collected under identical 980 nm excitation conditions (same power density, geometry, and acquisition settings). As shown in Figure R2a and R2b, both samples exhibit a dominant blue emission band centered at ~470 nm, consistent with Tm^{3+}-sensitized upconversion, indicating that PEI surface modification does not change the emission wavelength profile. To directly evaluate whether PEI coating affects the wavelength conversion efficiency, we further quantified the blue output and compared the emitted intensity at the same excitation power densities. The results (Figure R2c) show that UCNPs-PEI retains strong blue emission with a similar power-dependent trend and comparable emitting intensities to ligand-free UCNPs across the tested excitation intensities. Therefore, the PEI modification primarily alters the surface charge/colloidal properties while preserving the upconversion blue emission performance under 980 nm excitation.

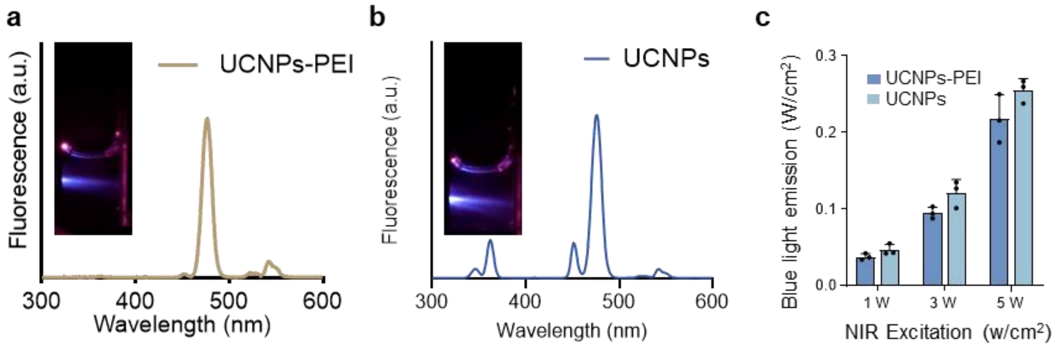

Fig. R2 Comparison of upconversion emission between UCNPs-PEI and ligand-free UCNPs

(a) Upconversion emission spectrum of UCNPs-PEI under 980 nm NIR excitation. Inset: photograph of UCNPs-PEI solution under 980 nm NIR illumination. **(b)** Upconversion emission spectrum of ligand-free UCNPs (before PEI modification) under 980 nm NIR excitation. Inset: photograph of the UCNPs solution under 980 nm NIR illumination. **(c)** Quantification of blue light emission intensity (~470 nm) from UCNPs-PEI and ligand-free UCNPs at different NIR excitation power densities (1, 3,

5 W/cm²). Data are mean ± SD from n=3 independent measurements.

Changed in the “Revised manuscript” (Lines 249-255):

Under 980 nm excitation, both ligand-free UCNPs (before PEI modification) and UCNPs-PEI (after PEI modification) exhibited a dominant blue emission band centered at ~470 nm (Fig. 2g and Supplementary Fig. 4a). A direct spectral comparison collected under identical acquisition settings indicates that PEI modification preserves the blue upconversion emission profile. The blue emission output was further quantified at the same excitation power densities, showing comparable blue light emission between ligand-free UCNPs and UCNPs-PEI (Supplementary Fig. 4b).

Changed in the “Revised manuscript” (Fig. 2, Page 14)

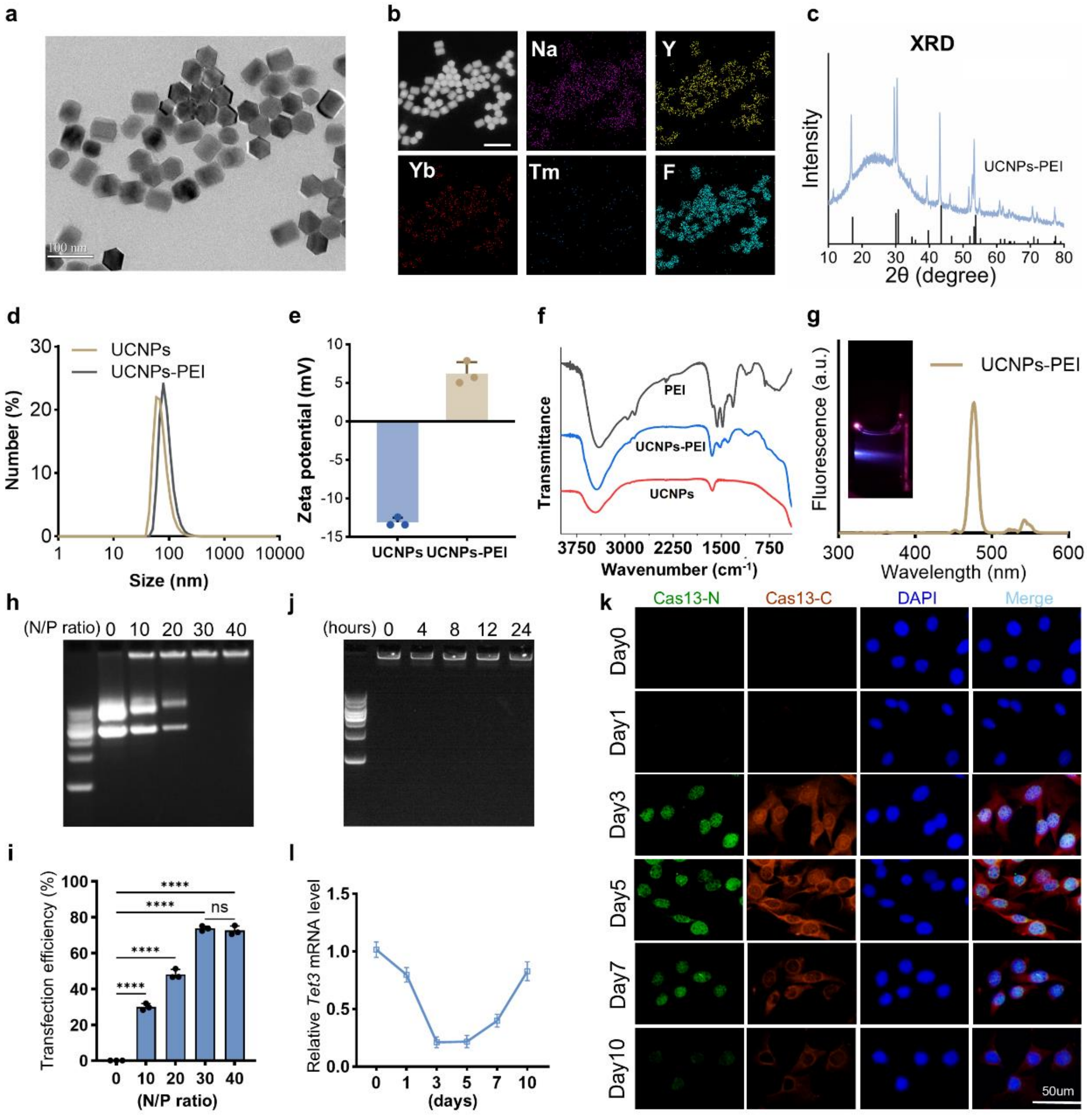

Fig. 2 Characterization of UCNPs-PEI

(a) Transmission electron microscopy (TEM) analysis showing monodisperse and uniform UCNPs-PEI (scale bar = 100 nm). **(b)** Elemental mapping analysis confirming

uniform distribution of Na, Y, Yb, Tm, and F within UCNPs-PEI. **(c)** X-ray diffraction (XRD) pattern of UCNPs-PEI. **(d)** DLS analysis of the hydrodynamic size distribution of ligand-free UCNPs and UCNPs-PEI. (e) Zeta potential analysis of UCNPs and UCNPs-PEI. (f) Fourier-transform infrared spectroscopy (FTIR) spectra analysis of UCNPs and UCNPs-PEI. (g) Upconversion emission spectrum of UCNPs-PEI under 980 nm NIR excitation. Inset: photograph of UCNPs-PEI solution under 980 nm NIR illumination. **(h)** Agarose gel electrophoresis of UCNPs-PEI@DNA complexes at different N/P ratios (0, 10, 20, 30, 40). **(i)** Transfection efficiency assay of UCNPs-PEI@GFP complexes at various N/P ratios. **(j)** Serum stability assay of UCNPs-PEI@paCas13d complexes over 24 hours. **(k)** Fluorescence microscopy of split-Cas13 expression (Cas13-N in green, Cas13-C in orange, DAPI in blue) at the indicated time points post-transfection in MLO-Y4 cells, scale bar = 50 µm. **(l)** Quantitative RT-PCR analysis of *Tet3* mRNA expression following UCNPs-PEI@paCas13d mediated knockdown over 10 days.

Changed in the "Supplementary Information" (Supplementary Fig.4, Page 8)

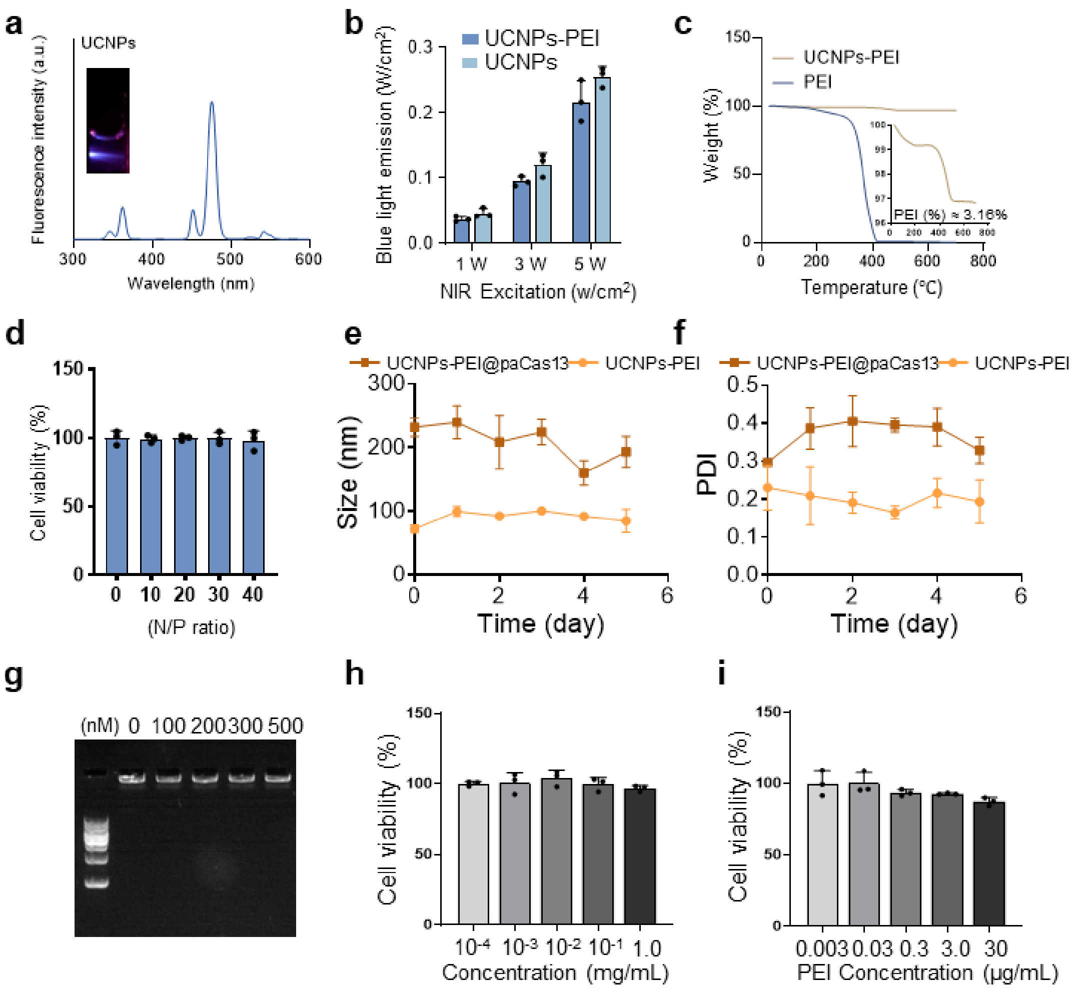

Supplementary Fig.4 Physicochemical and biological characterization of UCNPs-PEI nanoparticles

(a) Upconversion emission spectrum of UCNPs under 980 nm NIR excitation showing characteristic blue emission centered at ~470 nm. Inset: photograph of UCNPs under

980 nm laser illumination. **(b)** Quantification of blue light emission intensity from UCNPs-PEI and ligand-free UCNPs at different NIR excitation power densities (1, 3, 5 W/cm²). **(c)** Thermogravimetric analysis (TGA) of UCNPs-PEI. Inset: magnified view of the weight loss region. **(d)** Cell viability of HEK293T cells transfected with UCNPs-PEI@paCas13d complexes at N/P ratios from 0 to 40. **(e)** Hydrodynamic diameter of UCNPs-PEI and UCNPs-PEI@paCas13d measured by dynamic light scattering (DLS) over 5 days. **(f)** Polydispersity index (PDI) of UCNPs-PEI and UCNPs-PEI@paCas13d over 5 days. **(g)** Agarose gel electrophoresis of UCNPs-PEI@paCas13d complexes (N/P=30) after incubation in NaCl solutions of increasing ionic strength (0-500 mM) for 24 hours, demonstrating resistance to salt-induced dissociation. **(h)** Cell viability of HEK293T cells exposed to UCNPs-PEI at concentrations ranging from 10^{-4} to 1.0 mg/mL for 6 hours. **(i)** Cell viability of HEK293T cells exposed to free branched PEI (25 kDa) at concentrations equivalent to PEI content in UCNPs-PEI formulations (0.003-30 μg/mL) for 6 hours. Data are mean ± SEM (n=3).

6. It is stated that "UCNPs-PEI exhibited good biocompatibility (Figure S3c)." However, PEI is known to be highly cytotoxic. Why does UCNPs-PEI not show cytotoxicity even at a high concentration of 1 mg/mL?

Response: We thank the reviewer for raising this important safety concern. While free PEI is well-documented to exhibit concentration-dependent cytotoxicity, UCNPs-PEI demonstrates negligible toxicity due to the synergistic effect of low PEI loading and surface immobilization. Thermogravimetric analysis reveals that PEI constitutes only 3.16% of the UCNPs-PEI mass (Figure R3a, meaning that our highest tested concentration of 1 mg/mL UCNPs-PEI delivers only 31.6 μg/mL PEI—well below the established cytotoxic threshold.[8] Furthermore, surface-conjugated PEI exhibits dramatically reduced toxicity compared to free polymer, as nanoparticle immobilization constrains PEI conformation and shields cationic charges, thereby minimizing membrane disruption and cellular stress.[9] To rigorously validate these principles, we performed parallel cytotoxicity assays comparing UCNPs-PEI with equivalent concentrations of free PEI. As shown in Figures R3b and R3c, neither UCNPs-PEI nor free PEI at corresponding concentrations induced significant cytotoxicity, confirming that the effective PEI dose remains below toxic levels. Additionally, following standard PEI-mediated transfection protocols, cells were exposed to UCNPs-PEI for 6 hours, further limiting potential accumulation effects. The combination of low PEI content, surface immobilization, and limited exposure time accounts for the excellent biocompatibility of UCNPs-PEI despite the known toxicity of high-dose free PEI.

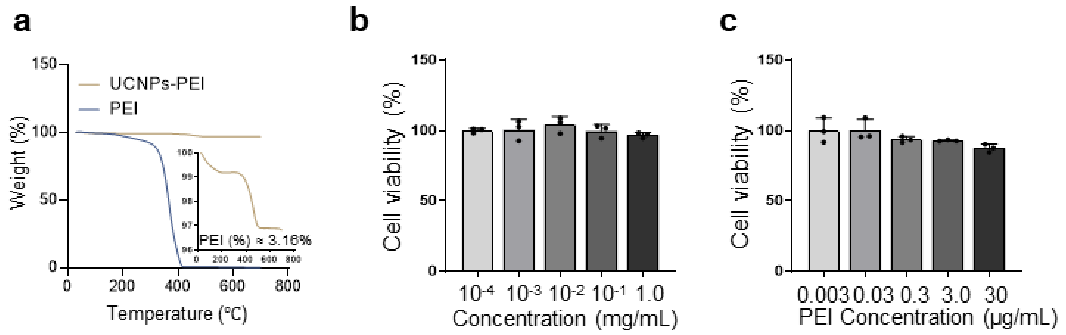

Fig. R3 Biocompatibility of UCNPs-PEI

(a) Thermogravimetric analysis showing PEI content in UCNPs-PEI. **(b)** Cell viability of MLO-Y4 cells after 6 h treatment with UCNPs-PEI at the indicated concentrations. **(c)** Cell viability after 6 h treatment with free PEI at concentrations equivalent to PEI content in UCNPs-PEI. Data are mean ± SEM (n=3).

Changed in the “Revised manuscript” (Lines 255-257)

In addition, thermogravimetric analysis indicated that PEI constituted approximately 3.16% of the UCNPs-PEI mass (Supplementary Fig. 4c).

Changed in the “Revised manuscript” (Lines 285-289)

Moreover, cell viability remained above 95% at UCNPs-PEI concentrations up to 1 mg/mL (Supplementary Fig. 4h). This acceptable biocompatibility is attributed to the low PEI content in the formulation (Supplementary Fig. 4c). Parallel testing with equivalent concentrations of free PEI showed no significant cytotoxicity (Supplementary Fig. 4i).

Changed in the “Supplementary Information” (Supplementary Fig.4, Page 8)

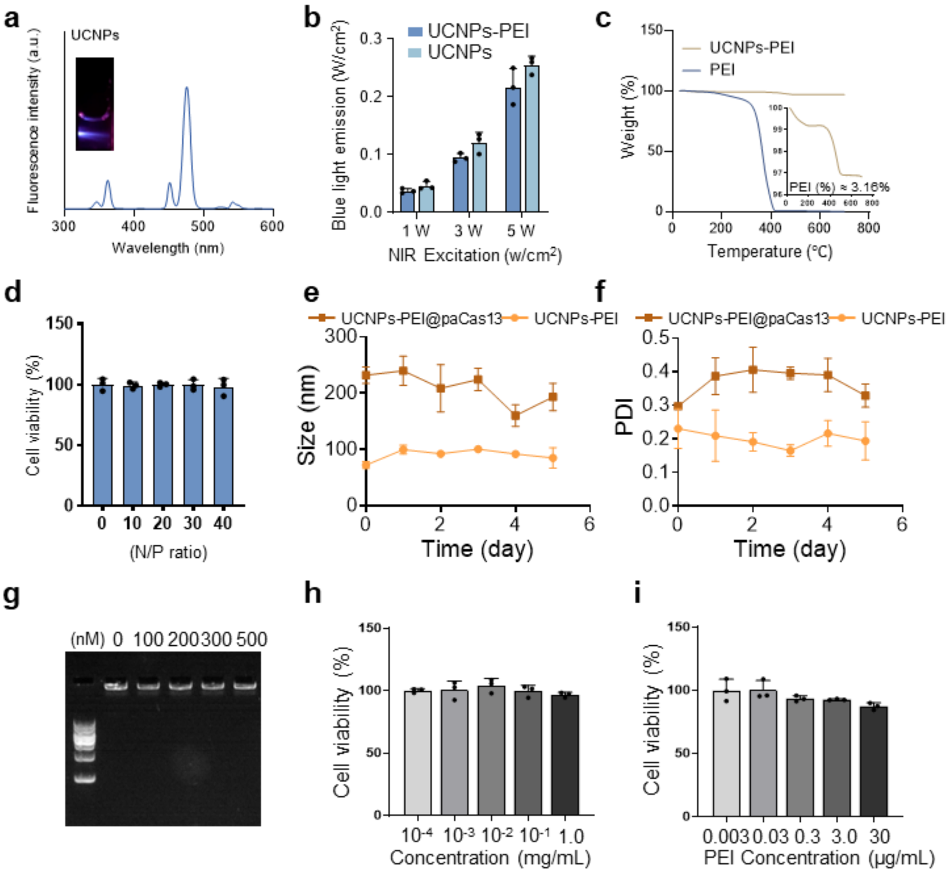

Supplementary Fig.4 Physicochemical and biological characterization of UCNPs-PEI nanoparticles

(a) Upconversion emission spectrum of UCNPs under 980 nm NIR excitation showing characteristic blue emission centered at ~470 nm. Inset: photograph of UCNPs under 980 nm laser illumination. **(b)** Quantification of blue light emission intensity from UCNPs-PEI and ligand-free UCNPs at different NIR excitation power densities (1, 3, 5 W/cm²). **(c)** Thermogravimetric analysis (TGA) of UCNPs-PEI. Inset: magnified

view of the weight loss region. **(d)** Cell viability of HEK293T cells transfected with UCNPs-PEI@paCas13d complexes at N/P ratios from 0 to 40. **(e)** Hydrodynamic diameter of UCNPs-PEI and UCNPs-PEI@paCas13d measured by dynamic light scattering (DLS) over 5 days. **(f)** Polydispersity index (PDI) of UCNPs-PEI and UCNPs-PEI@paCas13d over 5 days. **(g)** Agarose gel electrophoresis of UCNPs-PEI@paCas13d complexes (N/P=30) after incubation in NaCl solutions of increasing ionic strength (0-500 mM) for 24 hours, demonstrating resistance to salt-induced dissociation. **(h)** Cell viability of HEK293T cells exposed to UCNPs-PEI at concentrations ranging from 10^{-4} to 1.0 mg/mL for 6 hours. **(i)** Cell viability of HEK293T cells exposed to free branched PEI (25 kDa) at concentrations equivalent to PEI content in UCNPs-PEI formulations (0.003-30 μg/mL) for 6 hours. Data are mean ± SEM (n=3).

7. It is stated that "this NIR-induced editing efficiency was equivalent to that achieved by direct blue light activation of the padCas13 system." However, the intensity of blue light emitted directly from a blue light source should be vastly different from that emitted from NIR-excited UCNPs. Why, then, is the editing efficiency nearly equivalent?

Response: We thank the reviewer for this insightful question. To directly address this concern, we quantified the blue light emission from NIR-excited UCNPs and compared it with our direct blue light illumination conditions. As shown in Fig. R4, PEI-UCNPs under 1 W/cm² NIR excitation (980 nm) emit approximately 0.04 W/cm² of blue light (Fig. R4a), which exceeds the direct blue light intensity used in our experiments (0.002 W/cm²). Importantly, both illumination conditions deliver sufficient blue light to achieve maximal optogenetic activation, explaining the equivalent editing efficiencies (Fig. R4b). This demonstrates that NIR-mediated activation via upconversion achieves functional outcomes comparable to direct blue light activation, while offering the critical advantage of deep tissue penetration.

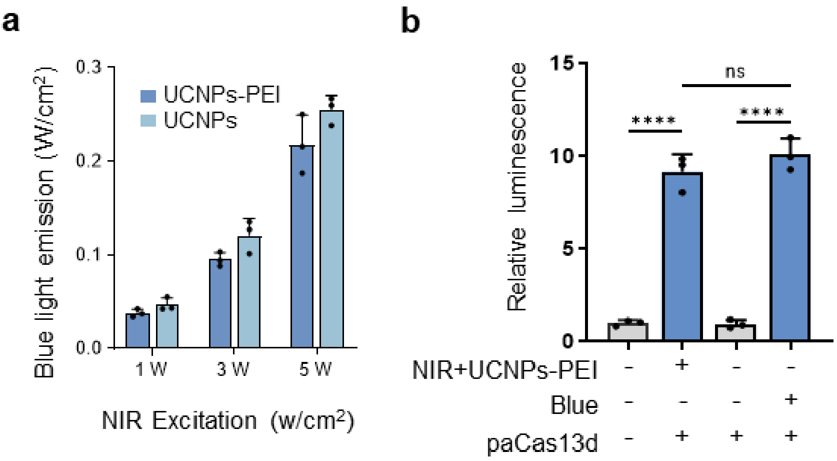

Fig. R4 Comparative analysis of blue light emission and RNA editing efficiency between NIR-excited UCNPs and direct blue light activation

(a) Quantification of blue light emission intensity from UCNPs-PEI and ligand-free UCNPs at different NIR excitation power densities (1, 3, 5 W/cm²). **(b)** A-to-I RNA base editing efficiency comparison using dual-luciferase reporter assay. NIR-excited UCNPs and direct blue light activation showed equivalent editing efficiency. (n=3, mean ± SD, $^{****}P < 0.0001$.

Changed in the "Revised manuscript" (Lines 338-347)

NIR illumination of cells transfected with UCNPs-PEI@padCas13 resulted in significant restoration of luciferase activity (Fig. 3c), achieving up to an 8.5-fold

increase comparable to direct blue light activation (Supplementary Fig.5). To understand why NIR-mediated activation achieved efficiency comparable to direct blue light, we quantified the blue light emission from NIR-excited UCNPs. Spectral analysis revealed that UCNPs under 1 W/cm² NIR excitation (980 nm) emit approximately 0.04 W/cm² of blue light (~470 nm), which exceeds the direct blue light intensity used in our experiments (0.002 W/cm², Supplementary Fig. 4b). Both illumination conditions thus deliver sufficient blue light doses to saturate optogenetic activation, explaining the equivalent editing efficiencies, while NIR offers the critical advantage of deep tissue penetration.

Changed in the “Supplementary Information” (Supplementary Fig.4, Page 8)

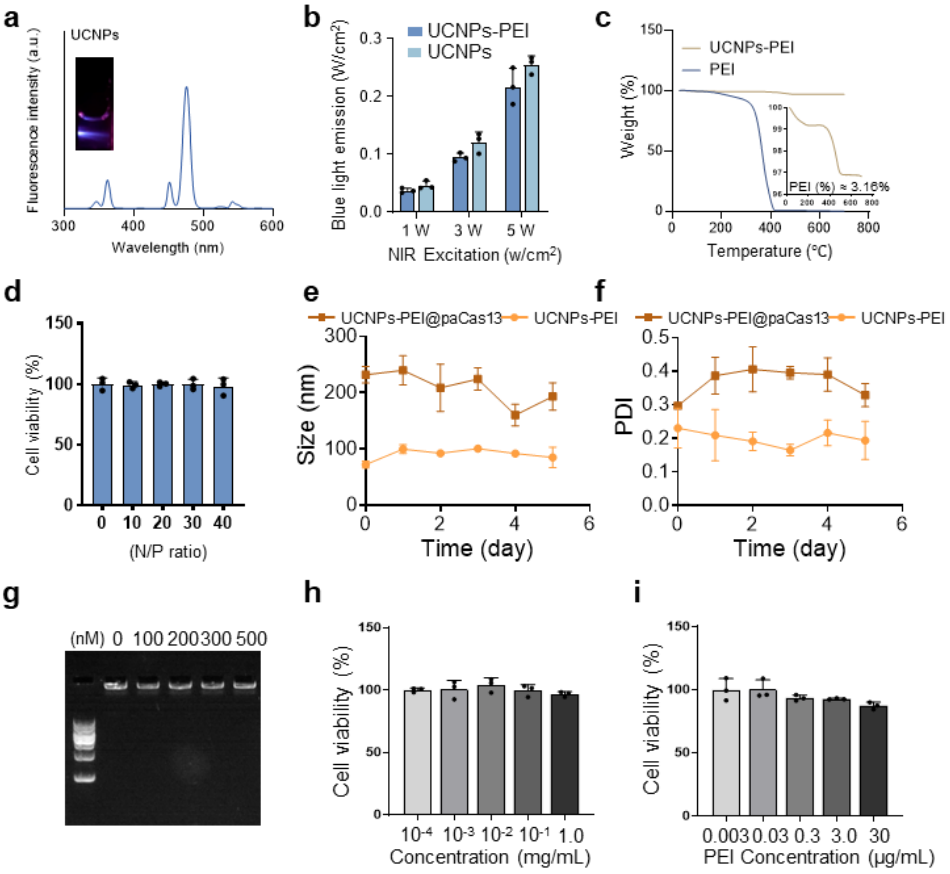

Supplementary Fig.4 Physicochemical and biological characterization of UCNPs-PEI nanoparticles

(a) Upconversion emission spectrum of UCNPs under 980 nm NIR excitation showing characteristic blue emission centered at ~470 nm. Inset: photograph of UCNPs under 980 nm laser illumination. **(b)** Quantification of blue light emission intensity from UCNPs-PEI and ligand-free UCNPs at different NIR excitation power densities (1, 3, 5 W/cm²). **(c)** Thermogravimetric analysis (TGA) of UCNPs-PEI. Inset: magnified view of the weight loss region. **(d)** Cell viability of HEK293T cells transfected with

UCNPs-PEI@paCas13d complexes at N/P ratios from 0 to 40. **(e)** Hydrodynamic diameter of UCNPs-PEI and UCNPs-PEI@paCas13d measured by dynamic light scattering (DLS) over 5 days. **(f)** Polydispersity index (PDI) of UCNPs-PEI and UCNPs-PEI@paCas13d over 5 days. **(g)** Agarose gel electrophoresis of UCNPs-PEI@paCas13d complexes (N/P=30) after incubation in NaCl solutions of increasing ionic strength (0-500 mM) for 24 hours, demonstrating resistance to salt-induced dissociation. **(h)** Cell viability of HEK293T cells exposed to UCNPs-PEI at concentrations ranging from 10^{-4} to 1.0 mg/mL for 6 hours. **(i)** Cell viability of HEK293T cells exposed to free branched PEI (25 kDa) at concentrations equivalent to PEI content in UCNPs-PEI formulations (0.003-30 μg/mL) for 6 hours. Data are mean ± SEM (n=3).

Changed in the “Supplementary Information” (Supplementary Fig.5, Page 9)

Supplementary Fig.5 Equivalent A-to-I editing efficiency between NIR-mediated

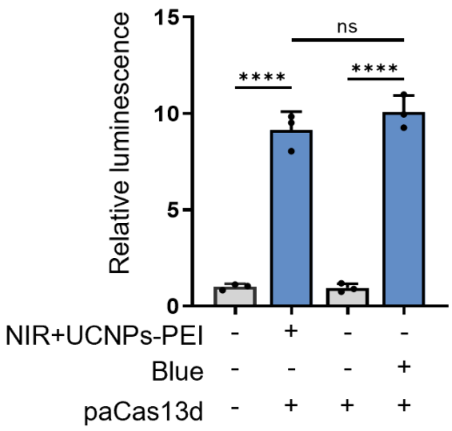

and direct blue light activation

Dual-luciferase reporter assay comparing A-to-I editing efficiency of padCas13d-$ADAR2_{DD}$ system under NIR-excited upconversion nanoparticles (NIR-UCNPs) versus direct blue light illumination. $^{****}P < 0.0001$.

8. UCNPs-PEI@paCas13d was administered to cells and mice to investigate therapeutic effects. How was UCNPs-PEI@paCas13d synthesized in the first place? If paCas13d is merely electrostatically adsorbed onto UCNPs-PEI, questions remain about its stability within cells and in the *bloodstream*.

Response: We thank the reviewer for this important methodological question. UCNPs-PEI@paCas13d complexes were prepared through electrostatic self-assembly by mixing UCNPs-PEI nanoparticles (1 mg/mL in 10 mM HEPES, pH 7.4) with plasmid DNA encoding the split paCas13d components at defined nitrogen-to-phosphate (N/P) ratios. We systematically optimized N/P ratios from 0 to 40 through gel retardation assay, transfection efficiency analysis, and cytotoxicity assessment. Complete DNA complexation was achieved at N/P $\geq$30, with transfection efficiency peaking at N/P = 30, beyond which further increases in N/P ratio yielded no additional improvement while maintaining excellent cell viability (Fig. R5a-R5c). Therefore, N/P = 30 was selected as the optimal formulation for all subsequent experiments.

Regarding stability concerns, we performed comprehensive validation across physiologically relevant conditions. Serum stability testing showed that complexes maintained structural integrity over 24 hours in the presence of serum proteins (Fig. R5d), confirming stability against protein-mediated displacement. Gel retardation assays demonstrated that UCNPs-PEI@paCas13d complexes remained stable in high ionic strength solutions (up to 500 mM NaCl, Fig. R5e), indicating resistance to dissociation under physiological salt concentrations. Most importantly, intracellular stability and functional persistence were directly demonstrated by transfecting MLO-Y4 cells with UCNPs-PEI@paCas13d and monitoring expression over time. Immunofluorescence analysis revealed sustained expression of both Cas13d-N and Cas13d-C fragments for up to 7 days post-transfection (Fig. R5f), with corresponding robust suppression of the target gene TET3 throughout this period (Fig. R5g). Collectively, these data demonstrate that electrostatic complexation provides sufficient stability for both cellular transfection and localized tissue delivery applications.

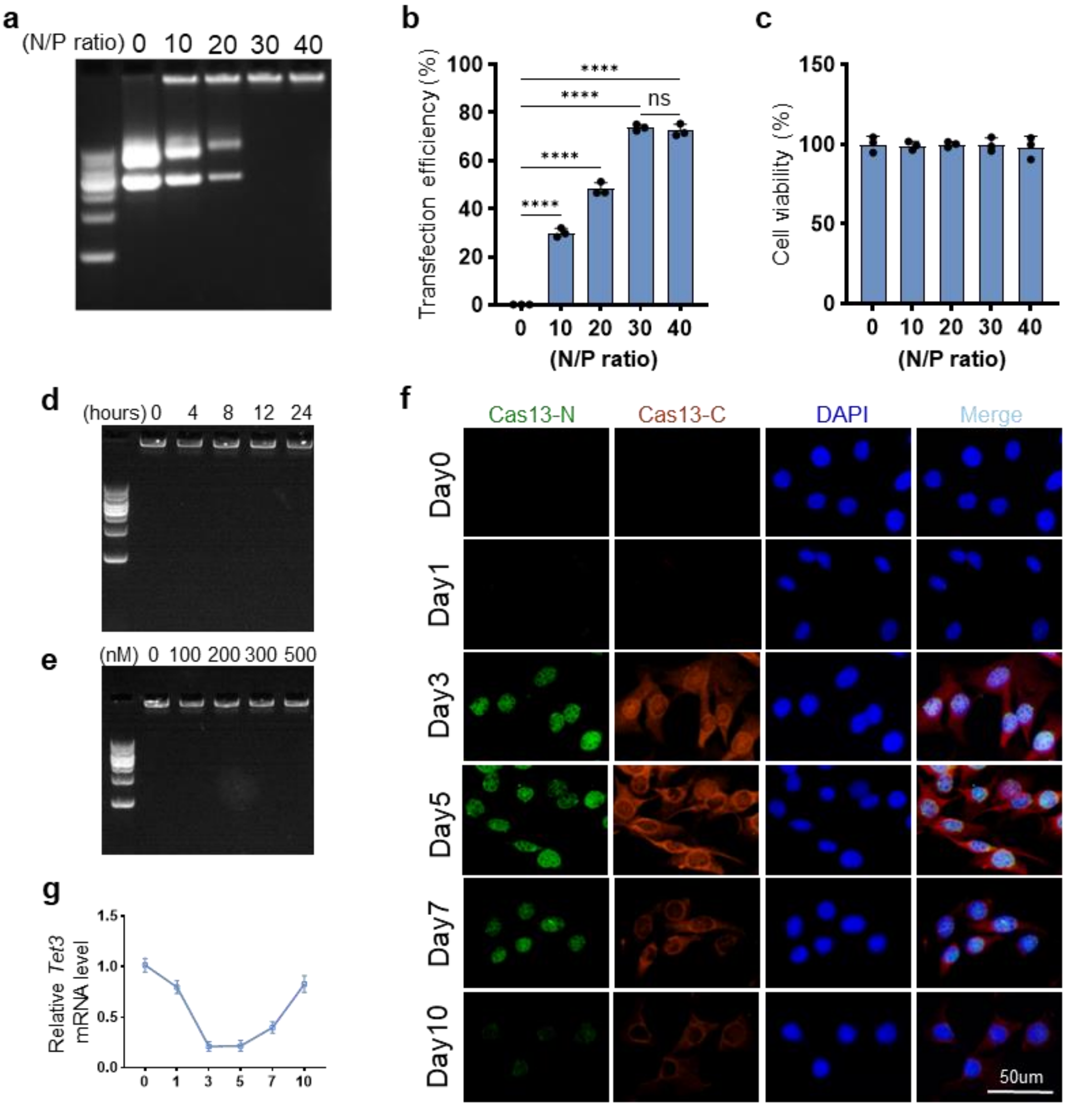

Fig. R5 Optimization of UCNPs-PEI mediated transfection of paCas13d

(a) Agarose gel electrophoresis of UCNPs-PEI@DNA complexes at different N/P ratios (0, 10, 20, 30, 40). **(b)** Transfection efficiency assay of UCNPs-PEI@GFP complexes in HEK293T cells at various N/P ratios. **(c)** Cell viability of HEK293T cells transfected with UCNPs-PEI@paCas13d complexes at N/P ratios from 0 to 40. **(d)** Serum stability assay of UCNPs-PEI@paCas13d complexes over 24 hours. **(e)** Agarose gel electrophoresis of UCNPs-PEI@paCas13d complexes (N/P=30) after incubation in NaCl solutions of increasing ionic strength (0-500 mM) for 24 hours, demonstrating resistance to salt-induced dissociation. **(f)** Fluorescence microscopy of split-Cas13 expression (Cas13-N in green, Cas13-C in orange, DAPI in blue) at the indicated time points post-transfection in MLO-Y4 cells, scale bar = 50 μm. **(g)** Quantitative RT-PCR analysis of *Tet3* mRNA expression following UCNPs-PEI@paCas13d mediated knockdown over 10 days.

Changed in the "Revised manuscript" (Lines 784-788)

UCNPs-PEI nanoparticles (1 mg/mL in 10 mM HEPES, pH 7.4) were mixed with plasmid DNA encoding the split paCas13d components at nitrogen-to-phosphate (N/P) ratios of 0-40. After systematic optimization, we determined that N/P = 30 provided optimal transfection efficiency with minimal cytotoxicity. The mixture was vortexed briefly and incubated for 30 min at room temperature to allow complex formation.

Changed in the "Revised manuscript" (Lines 258-285)

Having established the physicochemical properties of UCNPs-PEI, we systematically optimized the formulation parameters for efficient plasmid DNA delivery. The nitrogen-to-phosphate (N/P) ratio, representing the molar ratio of positively charged amine groups on PEI to negatively charged phosphate groups on DNA, is a critical determinant of complexation efficiency and transfection performance. Gel retardation assay showed complete DNA binding at N/P ≥ 30 (Fig. 2h), while transfection efficiency reached maximum at N/P = 30 with no further gains at higher ratios (Fig. 2i). Cell viability remained >95% across all tested conditions (Supplementary Fig. 4d). Based on these results, N/P = 30 was selected as the optimal formulation, achieving maximal gene delivery without compromising cell viability.

To assess stability under physiological conditions, we first characterized the complexation and colloidal properties of UCNPs-PEI@paCas13d. DLS measurements showed that plasmid loading increased the hydrodynamic diameter from 88.7 nm (UCNPs-PEI) to 232 nm (UCNPs-PEI@paCas13d), confirming successful DNA complexation (Supplementary Fig. 4e). Longitudinal assessment over 5 days revealed that both particle size and polydispersity index remained stable for DNA-loaded complexes (Supplementary Fig. 4e, f), indicating robust colloidal stability. We next challenged these complexes under physiologically relevant stress conditions. Gel electrophoresis confirmed that complexes remained largely intact after incubation in 10% fetal bovine serum (Fig. 2j) and resisted dissociation in 500 mM NaCl (Supplementary Fig. 4g), demonstrating resistance to both protein competition and ionic screening. These comprehensive stability assessments confirm the suitability of UCNPs-PEI@paCas13d for therapeutic applications. To assess functional persistence, we transfected MLO-Y4 osteoblast cells with UCNPs-PEI@paCas13d and monitored Cas13d expression and target gene suppression over time. Immunofluorescence analysis showed sustained expression of both Cas13d-N and Cas13d-C fragments for up to 7 days post-transfection, with peak expression at Days 3-5 (Fig. 2k). Correspondingly, *Tet3* mRNA levels remained significantly suppressed through Day 7 but began recovering toward baseline by Day 10 (Fig. 2l), defining a functional window of approximately one week and providing the rationale for weekly dosing in subsequent

in *vivo* studies.

Changed in the “Revised manuscript” (Fig. 2, Page 14)

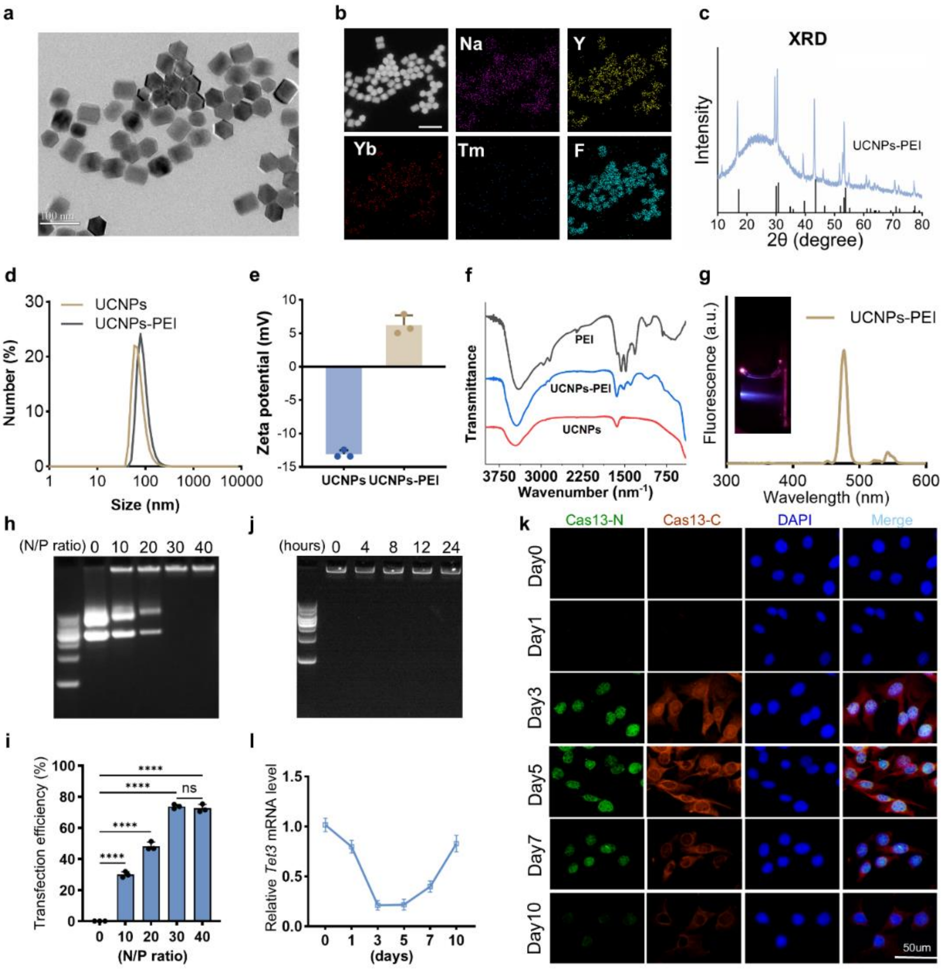

Fig. 2 Characterization of UCNPs-PEI

(a) Transmission electron microscopy (TEM) analysis showing monodisperse and uniform UCNPs-PEI (scale bar = 100 nm). **(b)** Elemental mapping analysis confirming uniform distribution of Na, Y, Yb, Tm, and F within UCNPs-PEI. **(c)** X-ray diffraction (XRD) pattern of UCNPs-PEI. **(d)** DLS analysis of the hydrodynamic size distribution of ligand-free UCNPs and UCNPs-PEI. **(e)** Zeta potential analysis of UCNPs and UCNPs-PEI. **(f)** Fourier-transform infrared spectroscopy (FTIR) spectra analysis of UCNPs and UCNPs-PEI. **(g)** Upconversion emission spectrum of UCNPs-PEI under 980 nm NIR excitation. Inset: photograph of UCNPs-PEI solution under 980 nm NIR illumination. **(h)** Agarose gel electrophoresis of UCNPs-PEI@DNA complexes at different N/P ratios (0, 10, 20, 30, 40). **(i)** Transfection efficiency assay of UCNPs-PEI@GFP complexes in HEK293T cells at various N/P ratios. **(j)** Serum stability assay of UCNPs-PEI@paCas13d complexes over 24 hours. **(k)** Fluorescence microscopy of split-Cas13 expression (Cas13-N in green, Cas13-C in orange, DAPI in blue) at indicated time points post-transfection in MLO-Y4 cells, scale bar = 50 μm. **(l)** Quantitative RT-PCR analysis of *Tet3* mRNA expression following UCNPs-

PEI@paCas13d mediated knockdown over 10 days.

Changed in the "Supplementary Information" (Supplementary Fig.4, Page 8)

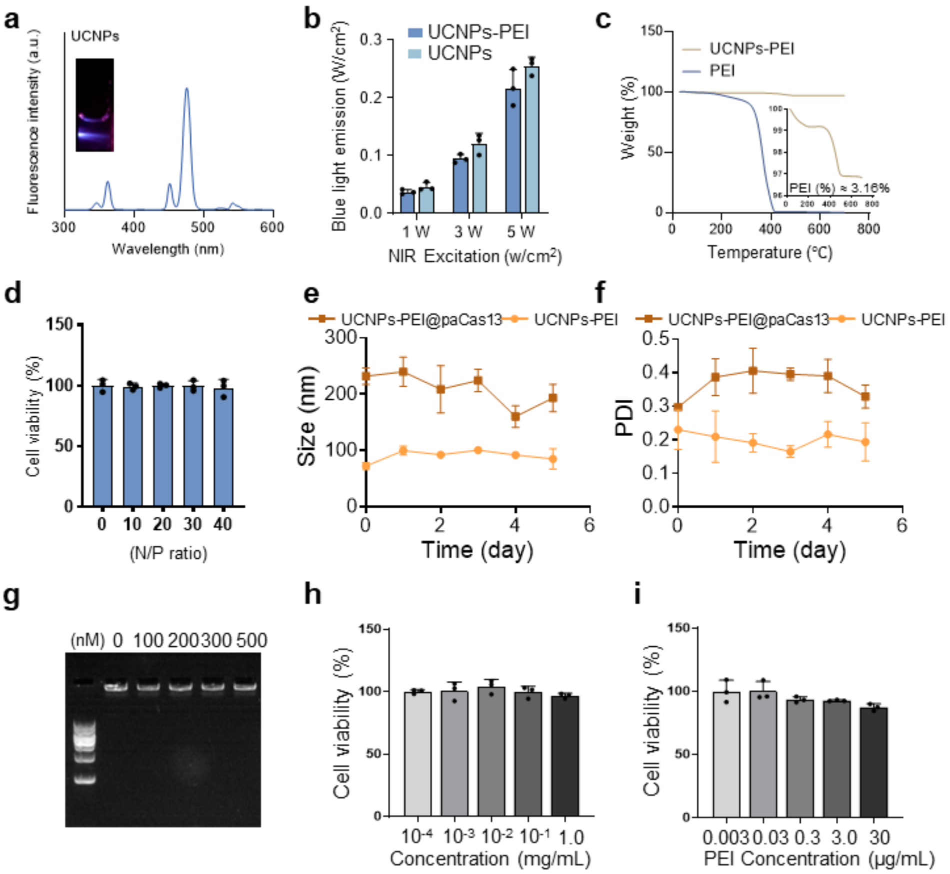

Supplementary Fig.4 Physicochemical and biological characterization of UCNPs-PEI nanoparticles

(a) Upconversion emission spectrum of UCNPs under 980 nm NIR excitation showing characteristic blue emission centered at ~470 nm. Inset: photograph of UCNPs under 980 nm laser illumination. **(b)** Quantification of blue light emission intensity from UCNPs-PEI and ligand-free UCNPs at different NIR excitation power densities (1, 3, 5 W/cm²). **(c)** Thermogravimetric analysis (TGA) of UCNPs-PEI. Inset: magnified view of the weight loss region. **(d)** Cell viability of HEK293T cells transfected with UCNPs-PEI@paCas13d complexes at N/P ratios from 0 to 40. **(e)** Hydrodynamic diameter of UCNPs-PEI and UCNPs-PEI@paCas13d measured by dynamic light scattering (DLS) over 5 days. **(f)** Polydispersity index (PDI) of UCNPs-PEI and UCNPs-PEI@paCas13d over 5 days. **(g)** Agarose gel electrophoresis of UCNPs-PEI@paCas13d complexes (N/P=30) after incubation in NaCl solutions of increasing ionic strength (0-500 mM) for 24 hours, demonstrating resistance to salt-induced dissociation. **(h)** Cell viability of HEK293T cells exposed to UCNPs-PEI at concentrations ranging from 10^{-4} to 1.0 mg/mL for 6 hours. **(i)** Cell viability of

HEK293T cells exposed to free branched PEI (25 kDa) at concentrations equivalent to PEI content in UCNPs-PEI formulations (0.003-30 μg/mL) for 6 hours. Data are mean ± SEM (n=3).

9. In addition, what pathway is used for UCNPs-PEI@paCas13d uptake into cells? If it is taken up via the endocytosis pathway, how can CRY2PHR and CIBN be delivered to the cell nucleus? If UCNPs-PEI@paCas13d is directly transfected into the cytoplasm, why do CRY2PHR and CIBN detach from UCNPs-PEI? Does xenophagy occur before this?

Response: We appreciate the reviewer's mechanistic question. UCNPs-PEI@paCas13d essentially functions as a typical PEI/DNA polyplex nanocarrier, and the intracellular delivery cascade of PEI-based gene vectors has been extensively described in the literature.[10, 11] In brief, the cationic PEI surface promotes adsorption to the cell membrane and initiates energy-dependent endocytosis (often dominated by clathrin-mediated uptake, with potential contributions from other endocytic routes depending on cell type and formulation). Following internalization, PEI-containing polyplexes can promote endosomal escape, which is often discussed in the context of PEI's buffering capacity (the "proton-sponge" framework), allowing the complexes to access the cytosol. Subsequently, polyplex decondensation releases plasmid DNA, enabling downstream nuclear entry and gene expression. Importantly, CRY2PHR and CIBN are not carried as proteins by the nanoparticles into the nucleus, and they are encoded by the delivered plasmids. After plasmid entry into the nucleus, the constructs are transcribed/translated, and the resulting fusion proteins reach their intended subcellular locations via canonical NLS/NES-guided trafficking and nuclear import machinery.

Regarding xenophagy, a number of nanoparticle and PEI-containing systems have been reported to interact with or induce autophagy pathways, which may contribute to intracellular processing/clearance after uptake.[12, 13] However, we did not directly assay xenophagy in this work. Nonetheless, our functional readouts indicate that sufficient plasmid delivery and expression occur within the experimental time window. We have added this clarification and toned down any causal claims.

Changed in the "Revised manuscript" (Lines 313-322)

The UCNPs-PEI@paCas13d essentially functions as a typical PEI/DNA polyplex nanocarrier, and the intracellular delivery cascade of PEI-based gene vectors has been extensively described in the literature.[10, 11] In brief, the cationic PEI surface promotes adsorption to the cell membrane and initiates energy-dependent endocytosis (often dominated by clathrin-mediated uptake, with potential contributions from other endocytic routes depending on cell type and formulation). Following internalization, PEI-containing polyplexes can promote endosomal escape, which is often discussed in the context of PEI's buffering capacity (the "proton-sponge" framework), allowing the complexes to access the cytosol. Subsequently, polyplex decondensation releases plasmid DNA, enabling downstream nuclear entry and gene expression (Fig. 3a).

Changed in the “Revised manuscript” (Fig. 3, Pages 17)

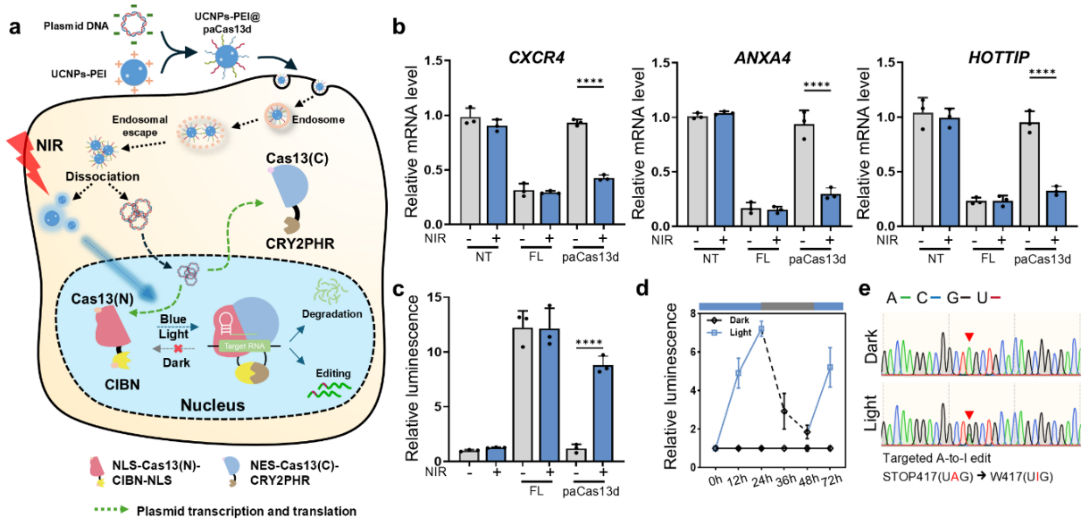

Fig. 3 UCNPs-PEI mediated paCas13d for NIR-activated RNA manipulation

(a) Schematic illustration of UCNPs-PEI delivery system for NIR-activated paCas13d. Split Cas13d fragments are delivered via PEI-functionalized upconversion nanoparticles. Upon NIR illumination (980 nm), UCNPs convert NIR to blue light (470 nm), triggering CRY2PHR/CIBN dimerization and functional reconstitution of paCas13d for targeted RNA degradation or base editing. **(b)** NIR-activated RNA knockdown efficiency of endogenous transcripts CXCR4, ANXA4, and HOTTIP. Comparison between non-targeting control (NT), full-length Cas13d (FL), and paCas13d under dark (-) and NIR illumination (+) conditions. **(c)** A-to-I RNA base editing activity assessed through dual-luciferase reporter rescue assay. padCas13-$ADAR2_{DD}$ system restores firefly luciferase activity by converting premature UAG stop codon to functional UGG codon exclusively under NIR illumination. **(d)** Temporal dynamics and reversibility of NIR-activated RNA base editing. Time-course analysis demonstrates progressive luciferase activity increase during NIR exposure followed by rapid decline upon light withdrawal, confirming temporal precision and reversible control. **(e)** Molecular confirmation of A-to-I editing through Sanger sequencing. Chromatograms show A-to-G conversion (red arrowheads) at position 417 in NIR-treated samples, converting UAG stop codon to UIG (read as UGG), while dark controls maintain original adenosine residue. Data are mean ± SEM (n=3). $^{****}P < 0.0001$.

10. How is UCNPs-PEI metabolized after remaining in the body?

Response: We thank the reviewer for this critical question regarding UCNPs-PEI metabolism and biodistribution. To comprehensively address this concern, we performed longitudinal *in vivo* imaging and tissue analysis following both local (intrafemoral) and systemic (intravenous) administration routes. For local intrafemoral injection, *in vivo* imaging revealed that UCNPs-PEI remained predominantly localized to the injected femur, with peak signals observed at Days 1-5 and gradual decline over 10 days (Fig. R6a). Importantly, we also detected accumulation in the liver, indicating hepatic clearance as the primary metabolic pathway. Immunofluorescence analysis of femoral tissue showed robust expression of Cas13d-N and Cas13d-C fragments at Days 3-7, followed by a significant reduction by Day 10 (Fig. R6b, R6c), consistent with gradual nanoparticle clearance and plasmid degradation. Correspondingly, *Tet3* mRNA levels in femoral tissue remained significantly suppressed through Day 7 but returned toward baseline by Day 10, while hepatic *Tet3* expression remained unchanged throughout the observation period, confirming target specificity (Fig. R6f). Based on this pharmacokinetic profile, indicating a therapeutic window of approximately 7 days, we implemented a weekly dosing regimen in our SAON treatment protocol to maintain sustained *Tet3* suppression.

To evaluate systemic distribution and metabolism, we performed intravenous administration of UCNPs-PEI@paCas13d. As expected for nanoparticles of this size (~200 nm), systemic injection resulted in rapid hepatic accumulation via reticuloendothelial system uptake, with peak signals at Days 1-5 and subsequent clearance by Day 10 (Fig. R7a). Notably, femoral accumulation was minimal following intravenous delivery, underscoring the advantage of local administration for bone-targeted therapy (Fig. R7b, R7c). Hepatic tissue analysis revealed transient Cas13d expression (Days 3-7, Fig. R7d, R7e), yet crucially, hepatic *Tet3* mRNA levels remained unchanged (Fig. R7f), indicating that despite hepatic nanoparticle accumulation, off-target gene silencing was negligible. This observation can be attributed to two factors: first, the spatial restriction of NIR light activation to the femoral site in our therapeutic protocol; second, the relatively low hepatic nanoparticle dose compared to the targeted femur. Taken together, these data demonstrate that UCNPs-PEI undergoes hepatobiliary clearance over 7-10 days, with our local delivery strategy achieving effective bone-targeted therapy while minimizing systemic exposure and off-target effects.

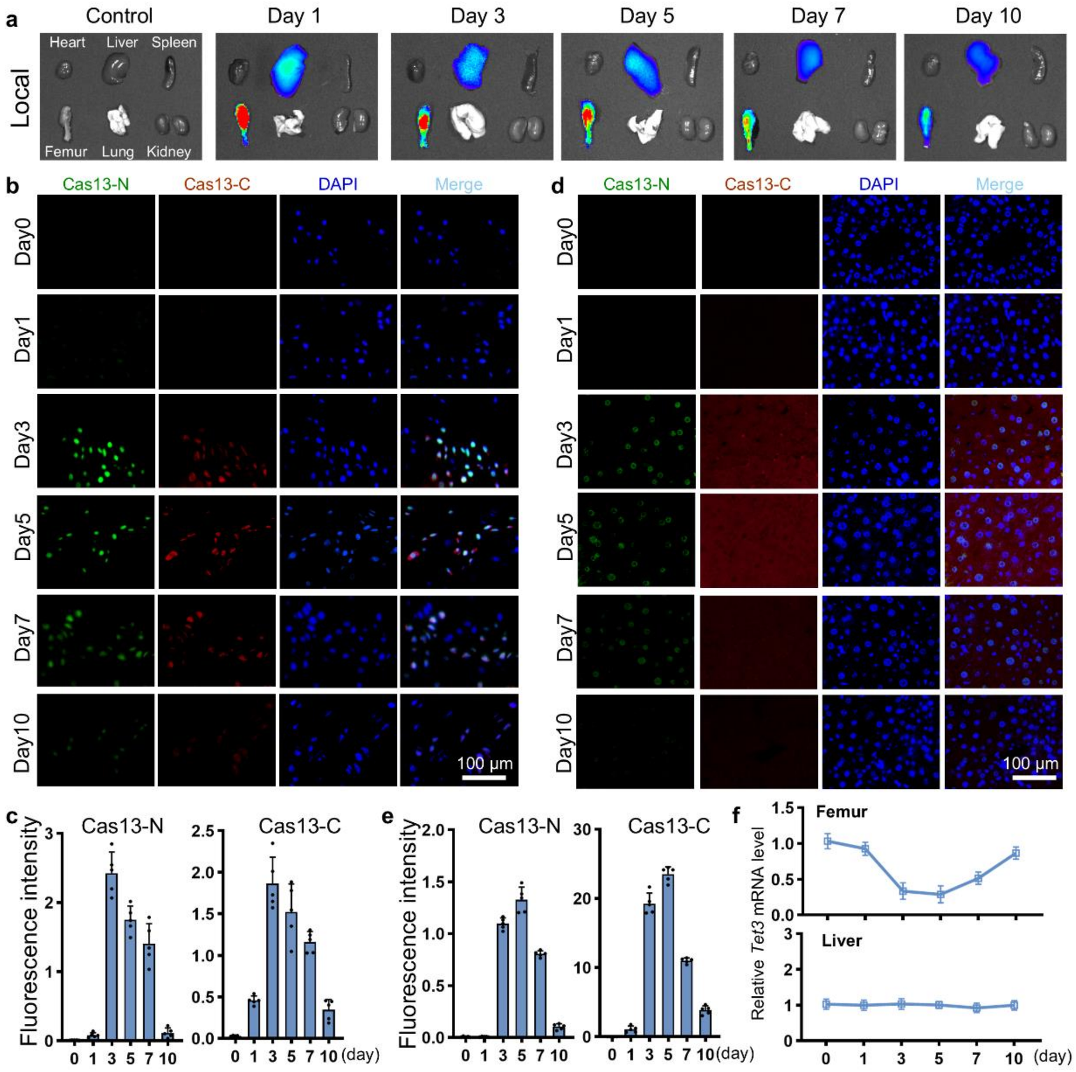

Fig. R6 Pharmacokinetics and biodistribution of UCNPs-PEI@paCas13d following intrafemoral injection

(a) Representative *in vivo* fluorescence imaging showing biodistribution of UCNPs-PEI@paCas13d at Days 1, 3, 5, 7, and 10 following intrafemoral injection. **(b)** Time-course immunofluorescence imaging of split-Cas13d expression in femoral tissue. Cas13d-N (green), Cas13d-C (orange), and nuclei (DAPI, blue) were detected at Days 0, 1, 3, 5, 7, and 10 post-injection. Scale bar = 100 μm. **(c)** Quantitative analysis of Cas13d-N and Cas13d-C fluorescence intensity in femoral tissue over time. **(d)** Immunofluorescence imaging of Cas13d expression in hepatic tissue at the same time points. Scale bar = 100 μm. **(e)** Quantitative analysis of Cas13d-N and Cas13d-C fluorescence intensity in liver tissue. **(f)** Quantitative RT-PCR analysis of *Tet3* mRNA levels in femur and liver tissues over 10 days. Data are mean ± SEM from n=5 animals per timepoint.

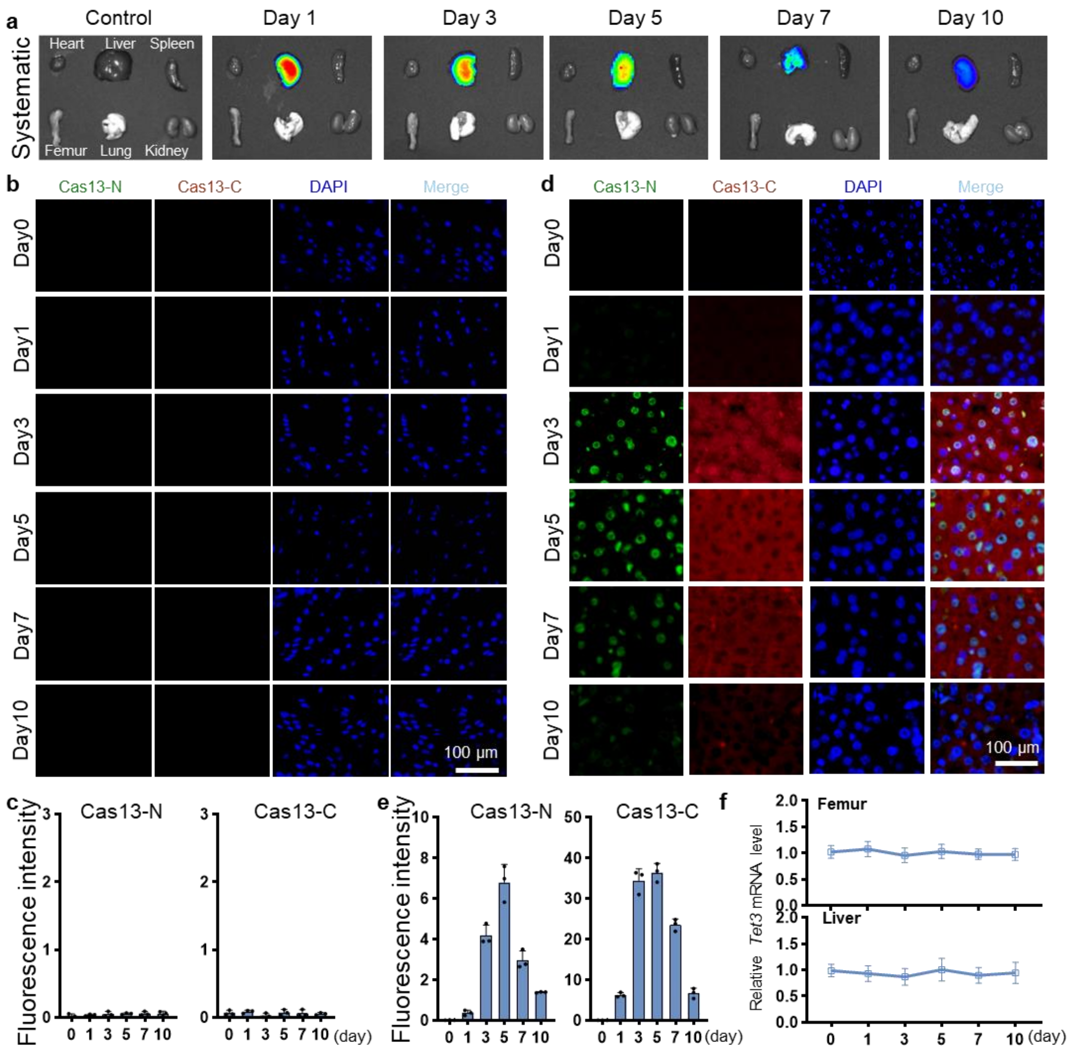

Fig. R7 Pharmacokinetics and biodistribution of UCNPs-PEI@paCas13d following systemic administration

(a) Representative *in vivo* imaging (heart, liver, spleen, femur, lung, kidney) following intravenous injection of UCNPs-PEI@paCas13d at Days 1, 3, 5, 7, and 10 post-injection. **(b,d)** Immunofluorescence staining of Cas13d-N (green) and Cas13d-C (red) in femoral bone tissue **(b)** and hepatic tissue **(d)** at indicated time points following intravenous administration. DAPI (blue) indicates nuclei. Scale bars, 100 μm. **(c,e)** Quantification of Cas13d-N and Cas13d-C fluorescence intensity in femoral **(c)** and hepatic **(e)** tissues over time. Data show mean fluorescence intensity in arbitrary units per timepoint across five animals. **(f)** Quantitative RT-PCR analysis of *Tet3* mRNA levels in femoral and hepatic tissues following intravenous administration. Data are mean ± SEM from n=5 animals per timepoint.

Changed in the "Revised manuscript" (Lines 451-488)

2.6 Pharmacokinetics and biodistribution of UCNPs-PEI@paCas13d *in vivo*

For safe and effective clinical translation, we comprehensively assessed the pharmacokinetics and biodistribution of UCNPs-PEI@paCas13d *in vivo*, to establish a rational dosing regimen and evaluate potential off-target effects. Following intrafemoral injection, longitudinal *in vivo* fluorescence imaging revealed that UCNPs-PEI remained predominantly localized to the injected femur, with peak fluorescence signals observed at Days 1-5 and gradual decline over 10 days (Fig. 5a). Notably, progressive hepatic accumulation was also detected, indicating hepatobiliary clearance as the primary metabolic pathway. To correlate nanoparticle distribution with functional gene delivery, we performed immunofluorescence analysis of femoral and hepatic tissues at multiple time points. In the femur, robust expression of both Cas13d-N and Cas13d-C fragments was observed at Days 3-7, with significant reduction by Day 10 (Fig. 5b, 5c), consistent with nanoparticle clearance and plasmid degradation kinetics. Most importantly, functional assessment revealed that femoral *Tet3* mRNA levels remained significantly suppressed through Day 7 but returned toward baseline by Day 10 (Fig. 5f), defining a therapeutic window of approximately 7 days. Critically, despite detectable Cas13d-N and Cas13d-C expression in hepatic tissue (Fig. 5d, 5e), hepatic *Tet3* mRNA levels remained unchanged throughout the 10-day observation period (Fig. 5f), demonstrating that paCas13d remains functionally inactive in non-illuminated tissues even when successfully delivered. Based on this pharmacokinetic profile, we implemented a weekly intrafemoral injection regimen for the subsequent SAON treatment protocol.

To directly compare delivery routes and further validate the necessity of local administration, we performed parallel biodistribution analysis following intravenous injection of UCNPs-PEI@paCas13d. As expected for nanoparticles in this size range (~200 nm), systemic administration resulted in rapid and predominant hepatic sequestration via reticuloendothelial system uptake, with peak signals at Days 1-5 and gradual clearance by Day 10 (Supplementary Fig. 8a). In stark contrast to intrafemoral injection, femoral accumulation following intravenous delivery was minimal, with correspondingly negligible Cas13d expression in femoral tissue (Supplementary Fig. 8b and 8c), rendering systemic delivery fundamentally inefficient for bone-targeted applications. Immunofluorescence analysis revealed transient hepatic Cas13d expression at Days 3-7 following intravenous administration (Supplementary Fig. 8d and 8e), yet hepatic *Tet3* mRNA levels remained unchanged (Supplementary Fig. 8f), indicating negligible functional gene silencing despite substantial nanoparticle accumulation. This critical observation demonstrates that the requirement for NIR light

activation provides spatial confinement of therapeutic activity—even when nanoparticles distribute systemically, gene editing occurs exclusively in illuminated tissues. These pharmacokinetic studies established the rationale for weekly intrafemoral dosing and confirmed light-gated spatial specificity. We therefore proceeded to evaluate therapeutic efficacy in a murine model of SAON.

Changed in the “Revised manuscript” (Fig. 5, Pages 23)

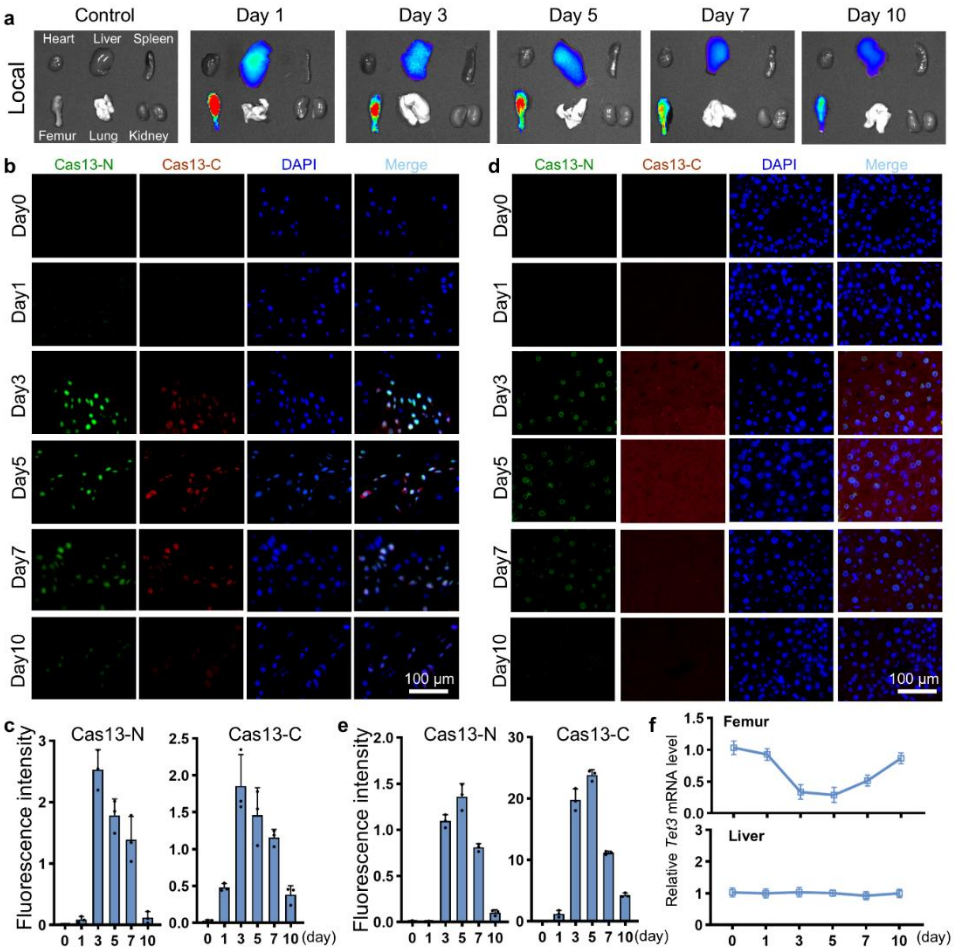

Fig. 5 Pharmacokinetics and biodistribution of UCNPs-PEI@paCas13d following intrafemoral injection

(a) Representative *in vivo* fluorescence imaging showing biodistribution of UCNPs-PEI@paCas13d at Days 1, 3, 5, 7, and 10 following intrafemoral injection. **(b)** Time-course immunofluorescence imaging of split-Cas13d expression in femoral tissue. Cas13d-N (green), Cas13d-C (orange), and nuclei (DAPI, blue) were detected at Days 0, 1, 3, 5, 7, and 10 post-injection. Scale bar = 100 μm. **(c)** Immunofluorescence imaging of Cas13d expression in hepatic tissue at the same time points. Scale bar = 100 μm. **(d)** Quantitative analysis of Cas13d-N and Cas13d-C fluorescence intensity in femoral tissue over time. **(e)** Quantitative analysis of Cas13d-N and Cas13d-C fluorescence intensity in liver tissue. **(f)** Quantitative RT-PCR analysis of *Tet3* mRNA levels in femur and liver tissues over 10 days. Data are mean ± SEM from n=5 animals per timepoint.

Changed in the "Supplementary Information" (Supplementary Fig.8, Pages 12)

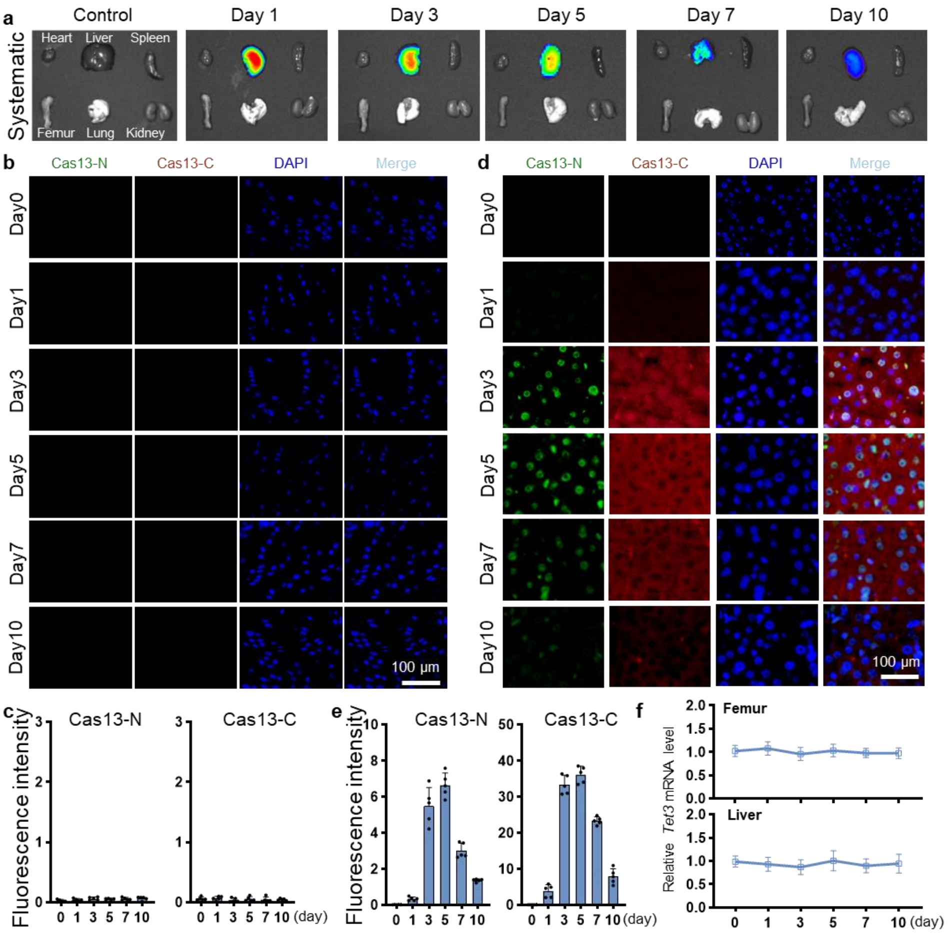

Supplementary Fig.8 Pharmacokinetics and biodistribution of UCNPs-PEI@paCas13d following systemic administration

(a) Representative *in vivo* imaging (heart, liver, spleen, femur, lung, kidney) following intravenous injection of UCNPs-PEI@paCas13d at Days 1, 3, 5, 7, and 10 post-injection. **(b,d)** Immunofluorescence staining of Cas13d-N (green) and Cas13d-C (red) in femoral bone tissue **(b)** and hepatic tissue **(d)** at indicated time points following intravenous administration. DAPI (blue) indicates nuclei. Scale bars, 100 μm. **(c,e)** Quantification of Cas13d-N and Cas13d-C fluorescence intensity in femoral **(c)** and hepatic **(e)** tissues over time. Data show mean fluorescence intensity in arbitrary units per timepoint across five animals. **(f)** Quantitative RT-PCR analysis of *Tet3* mRNA levels in femoral and hepatic tissues following intravenous administration. Data are mean ± SEM from n=5 animals per timepoint.

Reviewer #2 (Remarks to the Author):

The research group developed a novel photoactivatable CRISPR/Cas13d (paCas13d) system for precise, non-invasive RNA targeting in deep tissues. Furthermore, they engineered upconversion nanoparticles (UCNPs-PEI) to convert near-infrared light to blue light, enabling activation in bone tissue. In an in vivo mouse model of steroid-induced osteonecrosis, this system effectively knocked down TET3, preventing bone loss while preserving glucocorticoid function. This reported platform seems to offers spatiotemporal RNA control, combining CRISPR programmability with optical precision for localized therapeutic interventions with minimal systemic effects. Nevertheless, the chosen experimental design setup doesn't demonstrate the clinical need to such advanced system, most due to the omission of conventional controls.

1. Figure 1 The authors demonstrate photo-spatiotemporal control of the paCas13d system under blue light (470 nm). However, to confirm the specificity of the system, it is important to also show its lack of responsiveness to near-infrared (NIR) light under the same experimental conditions. This would strengthen the claim that the system is selectively activated by blue light and not by NIR, which is used elsewhere in the manuscript.

Response: We thank the reviewer for this important suggestion to demonstrate the blue light specificity of the paCas13d system. To directly address this concern, we performed control experiments exposing cells to NIR light (980 nm) in the absence of UCNPs. As shown in Fig. R8a, NIR illumination alone failed to induce knockdown of endogenous transcripts (*CXCR4*, *ANXA4*, *HOTTIP*), with mRNA levels remaining comparable to non-illuminated controls. Similarly, the padCas13-ADAR2 A-to-I editing system showed no luciferase rescue activity under NIR exposure without UCNPs (Fig. R8b), confirming the absence of direct NIR photosensitivity. These results unequivocally demonstrate that the paCas13d system is selectively activated by blue light (~470 nm) and exhibits no intrinsic responsiveness to NIR wavelengths. The functional activation observed with UCNPs+NIR treatment is therefore entirely attributable to upconversion-mediated generation of blue light, validating the wavelength specificity of our optogenetic control mechanism.

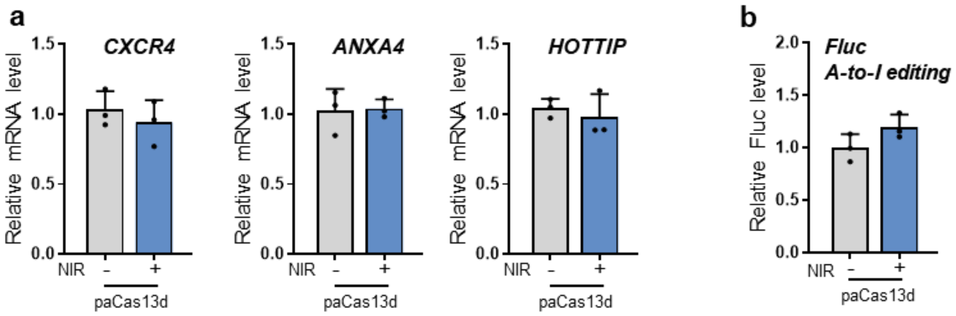

Fig. R8 Engineering of padCas13d for precision RNA base editing

(a) Quantitative RT-PCR analysis of endogenous transcript levels (*CXCR4*, *ANXA4*, *HOTTIP*) in HEK293T cells transfected with paCas13d and exposed to NIR light (980 nm) in the absence of upconversion nanoparticles. **(b)** Dual-luciferase reporter assay measuring A-to-I editing efficiency in cells transfected with padCas13d and exposed to NIR light without upconversion nanoparticles. Data are mean ± SEM (n=3).

Changed in the "Revised manuscript" (Lines 207-214)

To confirm that the photoactivatable system responds exclusively to blue light and not to NIR wavelengths used for upconversion activation, we performed critical control experiments. Exposure to NIR light (980 nm) in the absence of UCNPs failed to activate either paCas13d-mediated RNA degradation (Supplementary Fig. 2b) or padCas13-$ADAR2_{DD}$-mediated base editing (Supplementary Fig. 2c). These results unequivocally demonstrate that the CRY2PHR/CIBN optogenetic switch exhibits no intrinsic photosensitivity to NIR wavelengths, confirming that functional activation observed with UCNPs+NIR treatment is entirely attributable to upconversion-mediated generation of blue light.

Changed in the “Supplementary Information” (Supplementary Fig. 2, Pages 6)

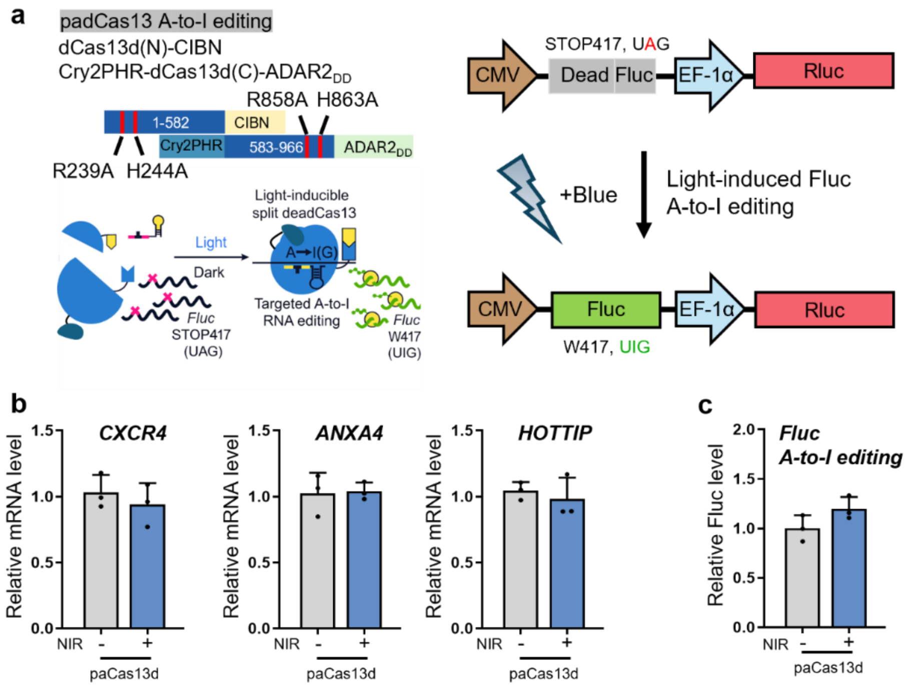

Supplementary Fig. 2 Engineering of padCas13d for precision RNA base editing **(a)** Schematic representation of the photoactivatable deadCas13d (padCas13d) system engineered for A-to-I RNA base editing. The catalytically inactive dCas13d is split at the optimized N582/C583 site, with the N-terminal fragment (1-582) fused to CIBN and the C-terminal fragment (583-966) fused to CRY2PHR and the ADAR2 deaminase domain ($ADAR2_{DD}$). Key catalytic residues R239A and H244A in the N-terminal HEPN domain and R858A and H863A in the C-terminal HEPN domain are mutated to abolish nuclease activity while preserving RNA binding. Upon blue light illumination (~470 nm), CRY2PHR undergoes conformational changes enabling heterodimerization with CIBN, reconstituting the split padCas13d-$ADAR2_{DD}$ complex for targeted A-to-I editing. **(b)** Quantitative RT-PCR analysis of endogenous transcript levels (*CXCR4*, *ANXA4*, *HOTTIP*) in HEK293T cells transfected with paCas13d and exposed to NIR light (980 nm) in the absence of upconversion nanoparticles. **(c)** Dual-luciferase reporter assay measuring A-to-I editing efficiency in cells transfected with padCas13d and exposed to NIR light without upconversion nanoparticles. Data are mean ± SEM (n=3).

2. Fig.1F & table S1. The manuscript does not specify which in silico CRISPR gRNA design platform was used to generate the gRNA sequences. Additionally, there is no information provided on the predicted knockdown (KD) efficiency of the designed gRNAs. Including these details is essential for evaluating the rationale behind the selection of gRNA sequences and their expected performance.

Response: We thank the reviewer for pointing out this important methodological detail. All gRNA sequences were designed using the Cas13 Design computational platform Cas13 Design (https://cas13design.nygenome.org/).[14] This platform employs machine learning algorithms trained on large-scale Cas13 screening datasets to predict on-target knockdown efficiency while minimizing off-target effects. For each target gene, multiple candidate crRNAs were computationally generated and ranked by predicted efficacy scores. The top-ranked sequence for each target was synthesized and employed in all cellular and *in vivo* experiments, demonstrating robust knockdown efficiency across all tested genes. These experimental results confirm the reliability of computational prediction in guiding crRNA selection. We have added this complete design methodology to the revised Methods section with appropriate citations, and all selected crRNA sequences are listed in Supplementary Table S1.

Changed in the “Revised manuscript” (Lines 711-718)

Guide RNAs targeting human *CXCR4*, *ANXA4*, *HOTTIP*, and mouse *Tet3* were designed using the Cas13 Design computational platform Cas13 Design (https://cas13design.nygenome.org/).[14] This platform employs machine learning algorithms trained on large-scale Cas13 screening datasets to predict on-target knockdown efficiency while minimizing off-target effects. For each target gene, multiple candidate crRNAs were computationally designed and ranked by predicted efficacy scores. Top-ranked sequences were synthesized and used in all subsequent cellular and *in vivo* experiments, and all guide sequences are listed in Supplementary Table S1.

3.Fig.2b Please include the polydispersity index (PDI) of UCNPs-PEI both before and after loading with the paCas13d system. Also, report the change in particle size upon loading. These parameters are important for assessing the stability and uniformity of the nanoparticle formulation.

Response: Thank you for such valuable reminders. As you suggested, we have now included both the hydrodynamic size and polydispersity index (PDI) of UCNPs-PEI before loading and UCNPs-PEI@paCas13d after loading, measured by DLS under identical conditions. As shown in Fig. R9, loading with the paCas13d plasmid increases the apparent hydrodynamic diameter relative to UCNPs-PEI, consistent with polyplex formation/complexation with nucleic acids. The average size increased from approximately 88.7 nm to 232 nm upon loading. Importantly, we also provide the time-dependent stability data (0-5 days), showing that both the particle size and PDI remain within a comparable range over time (Figure R9), supporting acceptable colloidal stability and formulation uniformity for downstream experiments.

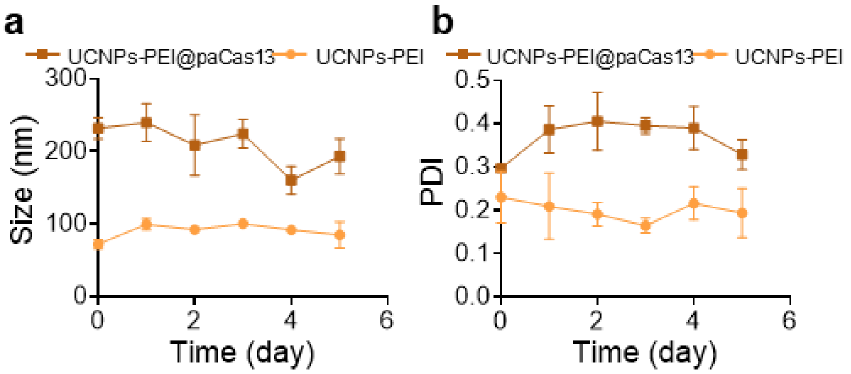

Fig. R9 Stability and uniformity of UCNPs-PEI@paCas13d complexes

(a) Hydrodynamic diameter of UCNPs-PEI and UCNPs-PEI@paCas13d measured by dynamic light scattering (DLS) over 5 days. **(b)** Polydispersity index (PDI) of UCNPs-PEI and UCNPs-PEI@paCas13d over 5 days.

Changed in the "Revised Manuscript" (Lines 267-273)

To assess stability under physiological conditions, we first characterized the complexation and colloidal properties of UCNPs-PEI@paCas13d. DLS measurements showed that plasmid loading increased the hydrodynamic diameter from 88.7 nm (UCNPs-PEI) to 232 nm (UCNPs-PEI@paCas13d), confirming successful DNA complexation (Supplementary Fig. 4e). Longitudinal assessment over 5 days revealed that both particle size and polydispersity index remained stable for DNA-loaded complexes (Supplementary Fig. 4e, f), indicating robust colloidal stability.

Changed in the "Supplementary Information" (Supplementary Fig. 4, Pages 8)

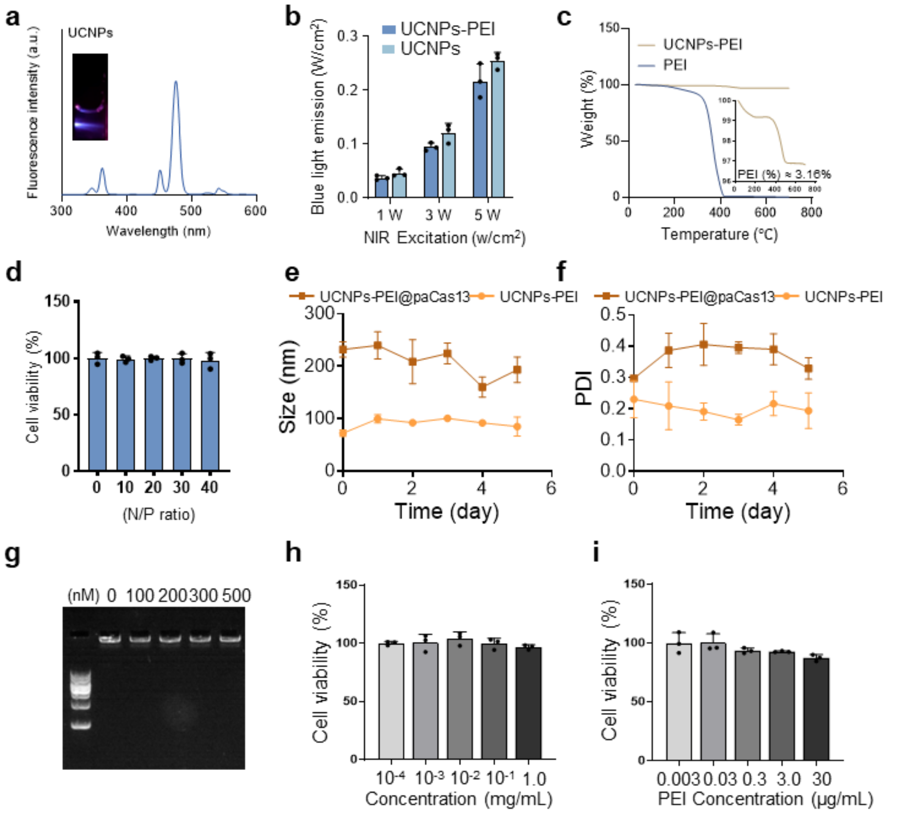

Supplementary Fig.4 Physicochemical and biological characterization of UCNPs-PEI nanoparticles

(a) Upconversion emission spectrum of UCNPs under 980 nm NIR excitation showing characteristic blue emission centered at ~470 nm. Inset: photograph of UCNPs under 980 nm laser illumination. **(b)** Quantification of blue light emission intensity from UCNPs-PEI and ligand-free UCNPs at different NIR excitation power densities (1, 3, 5 W/cm²). **(c)** Thermogravimetric analysis (TGA) of UCNPs-PEI. Inset: magnified view of the weight loss region. **(d)** Cell viability of HEK293T cells transfected with UCNPs-PEI@paCas13d complexes at N/P ratios from 0 to 40. **(e)** Hydrodynamic diameter of UCNPs-PEI and UCNPs-PEI@paCas13d measured by dynamic light scattering (DLS) over 5 days. **(f)** Polydispersity index (PDI) of UCNPs-PEI and UCNPs-PEI@paCas13d over 5 days. **(g)** Agarose gel electrophoresis of UCNPs-PEI@paCas13d complexes (N/P=30) after incubation in NaCl solutions of increasing ionic strength (0-500 mM) for 24 hours, demonstrating resistance to salt-induced dissociation. **(h)** Cell viability of HEK293T cells exposed to UCNPs-PEI at concentrations ranging from 10^{-4} to 1.0 mg/mL for 6 hours. **(i)** Cell viability of HEK293T cells exposed to free branched PEI (25 kDa) at concentrations equivalent to PEI content in UCNPs-PEI formulations (0.003-30 μg/mL) for 6 hours. Data are mean ± SEM (n=3).

4. Fig. S3c What was the loading efficiency of the paCas13d system into UCNPs-PEI? This efficiency is important in order to understand the dosage used in vitro and *in vivo*.

Response: We thank the reviewer for this important question regarding loading efficiency. As described in our optimization of the UCNPs-PEI@paCas13d formulation, we systematically evaluated N/P ratios from 0 to 40 through gel retardation assay, transfection efficiency analysis, and cytotoxicity assessment. The gel retardation assay revealed that complete DNA complexation was achieved at N/P $\geq$30, with no detectable free plasmid migration (Fig R10a), indicating loading efficiency exceeding 95%. N/P = 30 was selected as the optimal formulation because transfection efficiency peaked at this ratio, beyond which further increases yielded no additional improvement while maintaining excellent biocompatibility (Fig. R10b and R10c). This high loading efficiency enabled precise dosage control for both *in vitro* and *in vivo* applications.

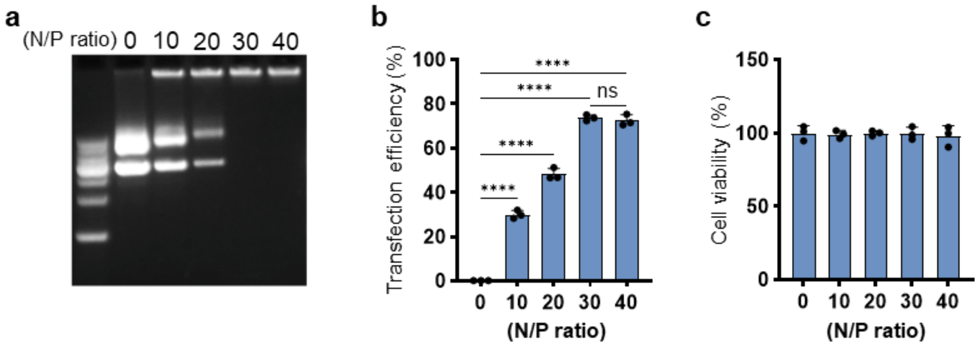

Fig R10 Optimization of N/P ratio and loading efficiency of UCNPs-PEI@paCas13d complexes

(a) Agarose gel electrophoresis of UCNPs-PEI@DNA complexes at different N/P ratios (0, 10, 20, 30, 40). **(b)** Transfection efficiency assay of UCNPs-PEI@GFP complexes at various N/P ratios. **(c)** Cell viability of HEK293T cells transfected with UCNPs-PEI@paCas13d complexes at N/P ratios from 0 to 40.

Changed in the "Revised Manuscript" (Lines 258-266)

Having established the physicochemical properties of UCNPs-PEI, we systematically optimized the formulation parameters for efficient plasmid DNA delivery. The nitrogen-to-phosphate (N/P) ratio, representing the molar ratio of positively charged amine groups on PEI to negatively charged phosphate groups on DNA, is a critical determinant of complexation efficiency and transfection performance. Gel retardation assay showed complete DNA binding at N/P $\geq$30 (Fig. 2h), while transfection efficiency reached maximum at N/P = 30 with no further gains at higher ratios (Fig. 2i). Cell viability remained >95% across all tested conditions (Supplementary Fig. 4d). Based on these results, N/P = 30 was selected as the optimal formulation, achieving maximal gene delivery without compromising cell viability.

Changed in the “Revised Manuscript” (Fig. 2 Pages 14)

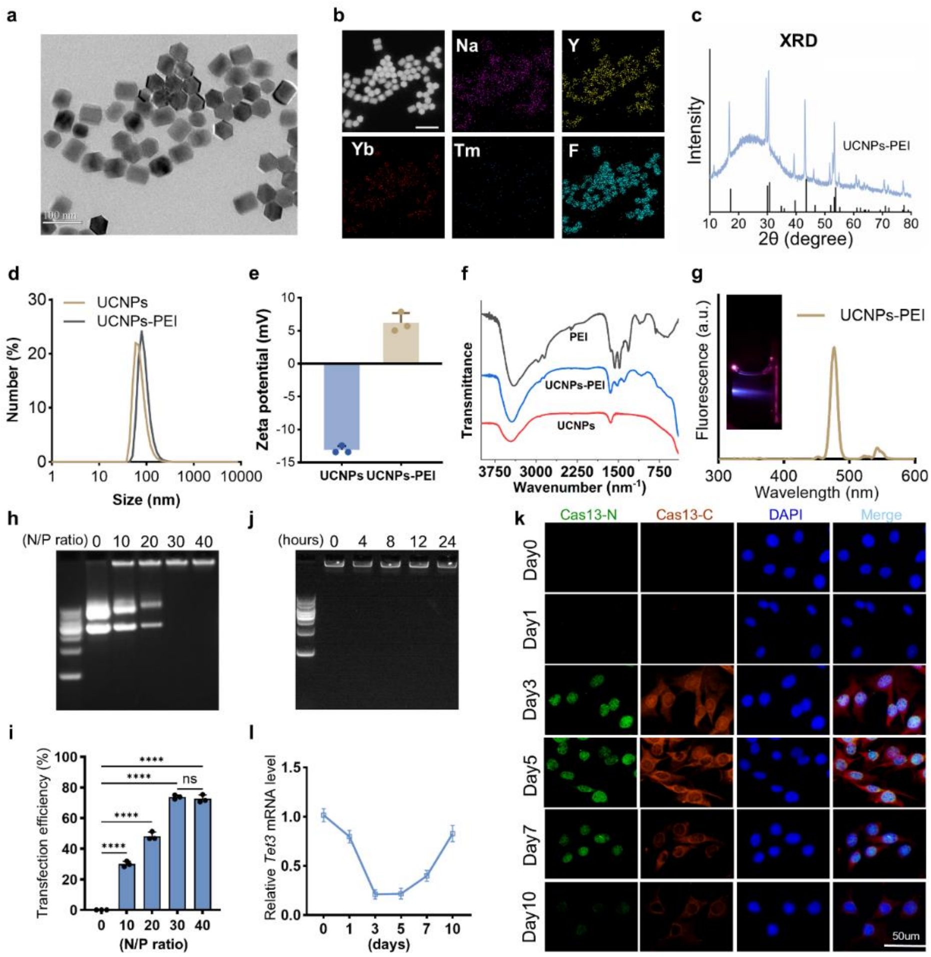

Fig. 2 Characterization of UCNPs-PEI

(a) Transmission electron microscopy (TEM) analysis showing monodisperse and uniform UCNPs-PEI (scale bar = 100 nm). **(b)** Elemental mapping analysis confirming uniform distribution of Na, Y, Yb, Tm, and F within UCNPs-PEI. **(c)** X-ray diffraction (XRD) pattern of UCNPs-PEI. **(d)** DLS analysis of hydrodynamic size distribution of ligand-free UCNPs and UCNPs-PEI. **(e)** Zeta potential analysis of UCNPs and UCNPs-PEI. **(f)** Fourier-transform infrared spectroscopy (FTIR) spectra analysis of UCNPs and UCNPs-PEI. **(g)** Upconversion emission spectrum of UCNPs-PEI under 980 nm NIR excitation. Inset: photograph of UCNPs-PEI solution under 980 nm NIR illumination. **(h)** Agarose gel electrophoresis of UCNPs-PEI@DNA complexes at different N/P ratios (0, 10, 20, 30, 40). **(i)** Transfection efficiency assay of UCNPs-PEI@GFP complexes at various N/P ratios. **(j)** Serum stability assay of UCNPs-PEI@paCas13d complexes over 24 hours. **(k)** Fluorescence microscopy of split-Cas13 expression (Cas13-N in green, Cas13-C in orange, DAPI in blue) at the indicated time points post-transfection in MLO-Y4 cells, scale bar = 50 μm. **(l)** Quantitative RT-PCR analysis of *Tet3* mRNA expression following UCNPs-PEI@paCas13d mediated knockdown over 10 days.

Changed in the "Supplementary Information" (Supplementary Fig. 4, Pages 8)

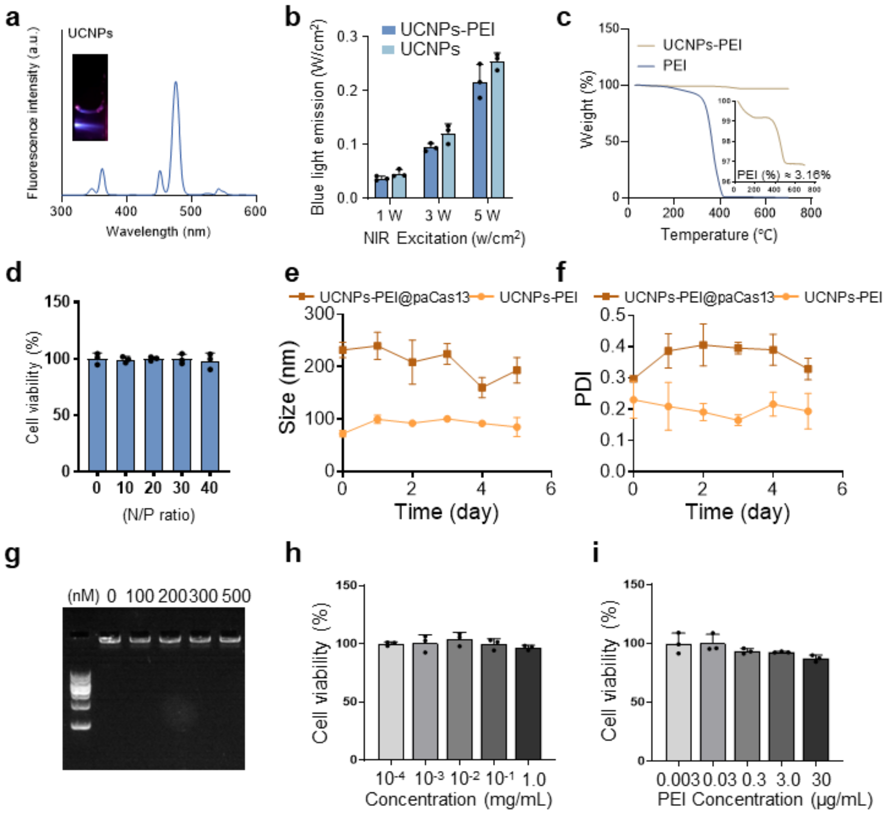

Supplementary Fig.4 Physicochemical and biological characterization of UCNPs-PEI nanoparticles

(a) Upconversion emission spectrum of UCNPs under 980 nm NIR excitation showing characteristic blue emission centered at ~470 nm. Inset: photograph of UCNPs under 980 nm laser illumination. **(b)** Quantification of blue light emission intensity from UCNPs-PEI and ligand-free UCNPs at different NIR excitation power densities (1, 3, 5 W/cm^2). **(c)** Thermogravimetric analysis (TGA) of UCNPs-PEI. Inset: magnified view of the weight loss region. **(d)** Cell viability of HEK293T cells transfected with UCNPs-PEI@paCas13d complexes at N/P ratios from 0 to 40. **(e)** Hydrodynamic diameter of UCNPs-PEI and UCNPs-PEI@paCas13d measured by dynamic light scattering (DLS) over 5 days. **(f)** Polydispersity index (PDI) of UCNPs-PEI and UCNPs-PEI@paCas13d over 5 days. **(g)** Agarose gel electrophoresis of UCNPs-PEI@paCas13d complexes (N/P=30) after incubation in NaCl solutions of increasing ionic strength (0-500 mM) for 24 hours, demonstrating resistance to salt-induced dissociation. **(h)** Cell viability of HEK293T cells exposed to UCNPs-PEI at concentrations ranging from 10^{-4} to 1.0 mg/mL for 6 hours. **(i)** Cell viability of HEK293T cells exposed to free branched PEI (25 kDa) at concentrations equivalent to PEI content in UCNPs-PEI formulations (0.003-30 μg/mL) for 6 hours. Data are mean ± SEM (n=3).

5. Nanoparticle Uptake Mechanism

The authors should describe the proposed cellular uptake mechanism of UCNPs-PEI. Clarifying this would help readers understand how the delivery system interacts with target cells and contributes to therapeutic efficacy.

Response: Thank you for such an important suggestion. The UCNPs-PEI@paCas13d essentially functions as a typical PEI/DNA polyplex nanocarrier, and the intracellular delivery cascade of PEI-based gene vectors has been extensively described in the literature.[10, 11] In brief, the cationic PEI surface promotes adsorption to the cell membrane and initiates energy-dependent endocytosis (often dominated by clathrin-mediated uptake, with potential contributions from other endocytic routes depending on cell type and formulation). Following internalization, PEI-containing polyplexes can promote endosomal escape, which is often discussed in the context of PEI's buffering capacity (the "proton-sponge" framework), allowing the complexes to access the cytosol. Subsequently, polyplex decondensation releases plasmid DNA, enabling downstream nuclear entry and gene expression.

Changed in the "Revised Manuscript" (Lines 313-322)

The UCNPs-PEI@paCas13d essentially functions as a typical PEI/DNA polyplex nanocarrier, and the intracellular delivery cascade of PEI-based gene vectors has been extensively described in the literature.[10, 11] In brief, the cationic PEI surface promotes adsorption to the cell membrane and initiates energy-dependent endocytosis (often dominated by clathrin-mediated uptake, with potential contributions from other endocytic routes depending on cell type and formulation). Following internalization, PEI-containing polyplexes can promote endosomal escape, which is often discussed in the context of PEI's buffering capacity (the "proton-sponge" framework), allowing the complexes to access the cytosol. Subsequently, polyplex decondensation releases plasmid DNA, enabling downstream nuclear entry and gene expression (Fig. 3a).

Changed in the “Revised Manuscript” (Fig. 3 Pages 17)

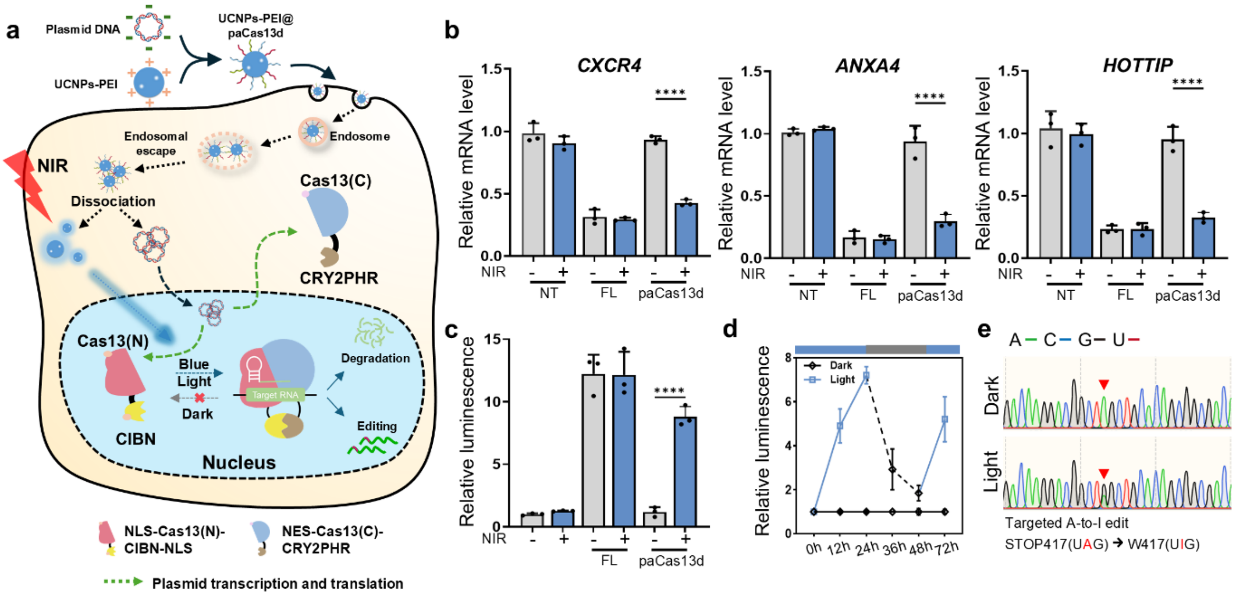

Fig. 3 UCNPs-PEI mediated paCas13d for NIR-activated RNA manipulation

(a) Schematic illustration of the UCNPs-PEI delivery system for NIR-activated paCas13d. Split Cas13d fragments are delivered via PEI-functionalized upconversion nanoparticles. Upon NIR illumination (980 nm), UCNPs convert NIR to blue light (470 nm), triggering CRY2PHR/CIBN dimerization and functional reconstitution of paCas13d for targeted RNA degradation or base editing. **(b)** NIR-activated RNA knockdown efficiency of endogenous transcripts CXCR4, ANXA4, and HOTTIP. Comparison between non-targeting control (NT), full-length Cas13d (FL), and paCas13d under dark (-) and NIR illumination (+) conditions. **(c)** A-to-I RNA base editing activity assessed through dual-luciferase reporter rescue assay. padCas13-$ADAR2_{DD}$ system restores firefly luciferase activity by converting premature UAG stop codon to functional UGG codon exclusively under NIR illumination. **(d)** Temporal dynamics and reversibility of NIR-activated RNA base editing. Time-course analysis demonstrates progressive luciferase activity increase during NIR exposure followed by rapid decline upon light withdrawal, confirming temporal precision and reversible control. **(e)** Molecular confirmation of A-to-I editing through Sanger sequencing. Chromatograms show A-to-G conversion (red arrowheads) at position 417 in NIR-treated samples, converting UAG stop codon to UIG (read as UGG), while dark controls maintain original adenosine residue. Data are mean ± SEM (n=3). $^{****}P < 0.0001$.

Changed in the “Revised Manuscript” (Fig. 7, Pages 29)

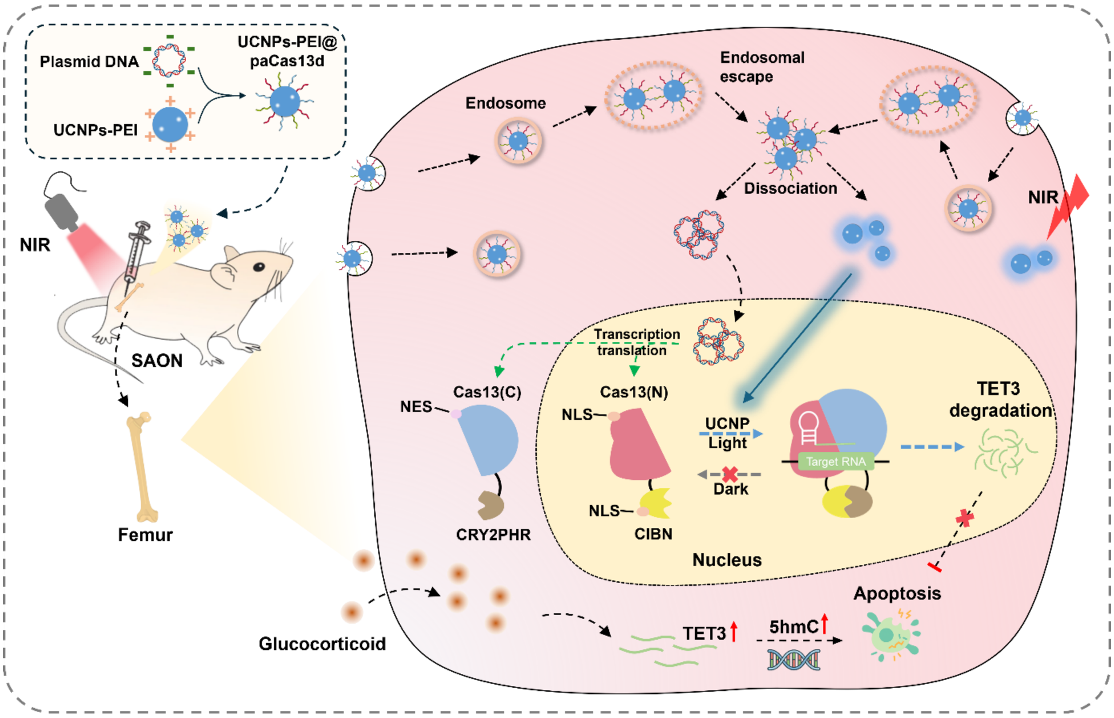

Fig. 7 Schematic illustration of the UCNPs-PEI@paCas13d system for preventing SAON

Comprehensive overview of the NIR-activated paCas13 system for localized prevention of SAON. UCNPs-PEI nanoparticles serve as gene delivery vectors and photon transducers, facilitating efficient transfection of split paCas13d components into target cells via intrafemoral injection to bone tissue. Upon NIR illumination, UCNPs convert NIR to blue light, triggering CRY2PHR/CIBN heterodimerization and functional reconstitution of Cas13d. The activated paCas13d specifically degrades TET3 mRNA, preventing glucocorticoid-induced TET3 upregulation and subsequent epigenetic reprogramming that leads to osteocyte apoptosis. This spatially-restricted intervention protects bone tissue from steroid-induced damage while preserving systemic glucocorticoid therapeutic effects. The modular design enables precise temporal and spatial control of RNA therapeutic intervention through non-invasive external light activation.

6. *In-vivo* study (figure 5) It is unclear why a siRNA control group was not included to benchmark the therapeutic efficacy of the paCas13d system. The authors should address this omission in the limitations section.

Response: We thank the reviewer for this important suggestion. We have now included a siRNA control group targeting TET3 to benchmark the therapeutic efficacy of our paCas13d system. As shown in Fig. R11, siRNA-mediated TET3 knockdown achieved comparable therapeutic outcomes in the femur, with significant suppression of femoral TET3 expression (Fig. R11a), preservation of trabecular bone microarchitecture (Fig. R11b, R11c), and reduction in osteocyte apoptosis (Fig. R11e, R11f). These results confirm that TET3 suppression is sufficient to prevent SAON, validating our therapeutic target.

However, critical differences emerged regarding off-target effects and safety profiles. *In vivo* biodistribution analysis revealed that while both delivery systems accumulated in the injected femur, siRNA also exhibited substantial hepatic accumulation (Fig. R11g). Importantly, this hepatic distribution resulted in unintended TET3 suppression in the liver (Fig. R11h), which was accompanied by significant elevation of serum transaminases (Fig. R11i), indicating hepatotoxicity. In stark contrast, the UCNPs-PEI@paCas13d group showed no hepatic TET3 suppression despite similar hepatic nanoparticle accumulation (Fig. R11g, R11j), and liver function remained normal (Fig. R11k). This critical distinction demonstrates the unique advantage of our photoactivatable system: the requirement for both molecular delivery and optical activation provide spatial specificity that prevents off-target gene editing in non-illuminated tissues, even when the delivery vehicle distributes systemically. While siRNA achieves equivalent on-target efficacy, the UCNPs-PEI@paCas13d system offers superior safety through light-gated control, making it more suitable for localized therapeutic applications where systemic gene suppression is undesirable.

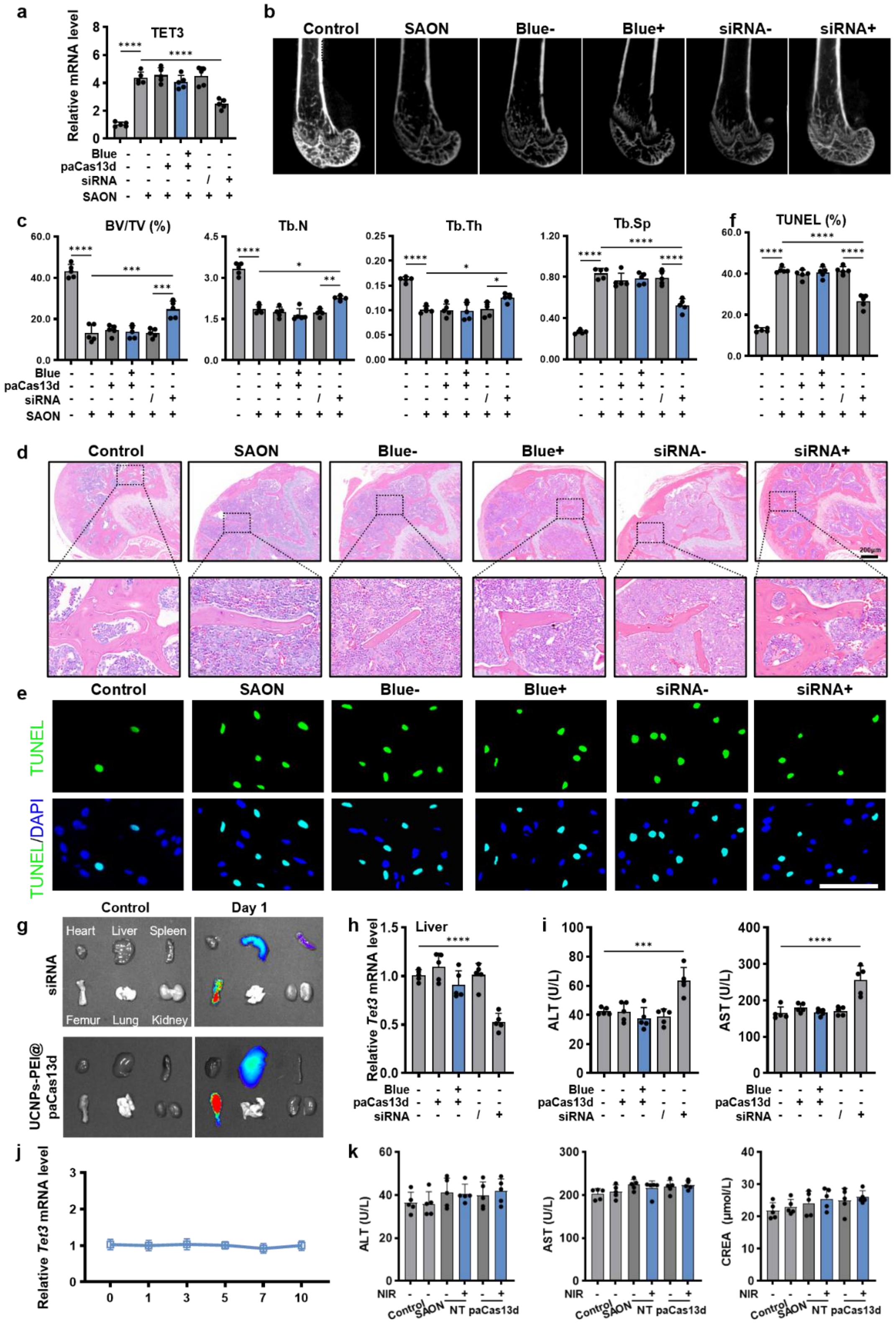

Fig. R11 Evaluation of blue light activation and siRNA-mediated *Tet3* knockdown in SAON model

(a) Quantitative RT-PCR analysis of femoral *Tet3* mRNA levels across treatment groups, including siRNA-mediated TET3 knockdown and direct blue light activation of paCas13d without UCNPs. / : scrambled siRNA control. **(b)** Representative micro-

CT 3D reconstruction images of femur trabecular bone. **(c)** Quantitative micro-CT analysis of trabecular bone parameters including BV/TV, Tb.N, Tb.Th, and Tb.Sp. **(d)** Hematoxylin and eosin (H&E) staining of femur trabecular bone. Upper panels show low magnification overview; lower panels show high magnification of trabecular microarchitecture within boxed regions. Scale bars = 200 μm. **(e)** Representative TUNEL staining (green) of femoral sections showing apoptotic osteocytes. DAPI (blue) counterstains nuclei. Scale bar, 50 μm. **(f)** Quantitative analysis of TUNEL-positive osteocytes. **(g)** *In vivo* organ imaging of UCNPs-PEI@paCas13d and siRNA at Day 1 post-injection. **(h)** Quantitative RT-PCR analysis of hepatic *Tet3* mRNA levels to assess potential off-target gene suppression across treatment groups. **(i)** Serum alanine aminotransferase (ALT) and aspartate aminotransferase (AST) levels are measured as indicators of liver function. **(j)** Quantitative RT-PCR analysis of *Tet3* mRNA levels in liver tissues over 10 days following intrafemoral administration of UCNPs-PEI@paCas13d. **(k)** Serum biochemical markers evaluating liver and kidney function following UCNPs-PEI@paCas13d treatment. Data are mean ± SEM, n=5 animals per group. $^{****}P < 0.0001$, $^{***}P < 0.001$, $^{**}P < 0.01$, $^{*}P < 0.05$.

Changed in the “Revised Manuscript” (Lines 573-589)

To further establish the necessity of UCNPs-mediated NIR activation and benchmark therapeutic efficacy *in vivo*, we evaluated two critical controls in the SAON model. We compared siRNA-mediated *Tet3* knockdown against our photoactivatable system and tested whether direct blue light could activate UCNPs-PEI@paCas13d in deep bone tissue without upconversion. Both controls revealed fundamental limitations that our photoactivatable nanoparticle system overcomes. The siRNA control group, administered via intrafemoral injection, achieved comparable therapeutic efficacy in the target femur, demonstrating significant *Tet3* suppression (Supplementary Fig. 14a), preservation of trabecular bone microarchitecture (Supplementary Fig. 14b-14d), and reduction in osteocyte apoptosis (Supplementary Fig. 14e and 14f). However, despite local administration, siRNA exhibited systemic redistribution to the liver, as evidenced by biodistribution imaging (Supplementary Fig. 14g). Critically, this hepatic accumulation resulted in unintended off-target *Tet3* suppression (Supplementary Fig. 14h) and significant hepatotoxicity, with elevated serum transaminases (Supplementary Fig. 14i). In stark contrast, the UCNPs-PEI@paCas13d+NIR group, despite similar hepatic nanoparticle accumulation, showed no hepatic *Tet3* suppression and maintained normal liver function (Supplementary Fig. 13), demonstrating that light-gated spatial control prevents off-target gene editing in non-illuminated tissues.

Changed in the “Revised Manuscript” (Fig. 5, Pages 23)

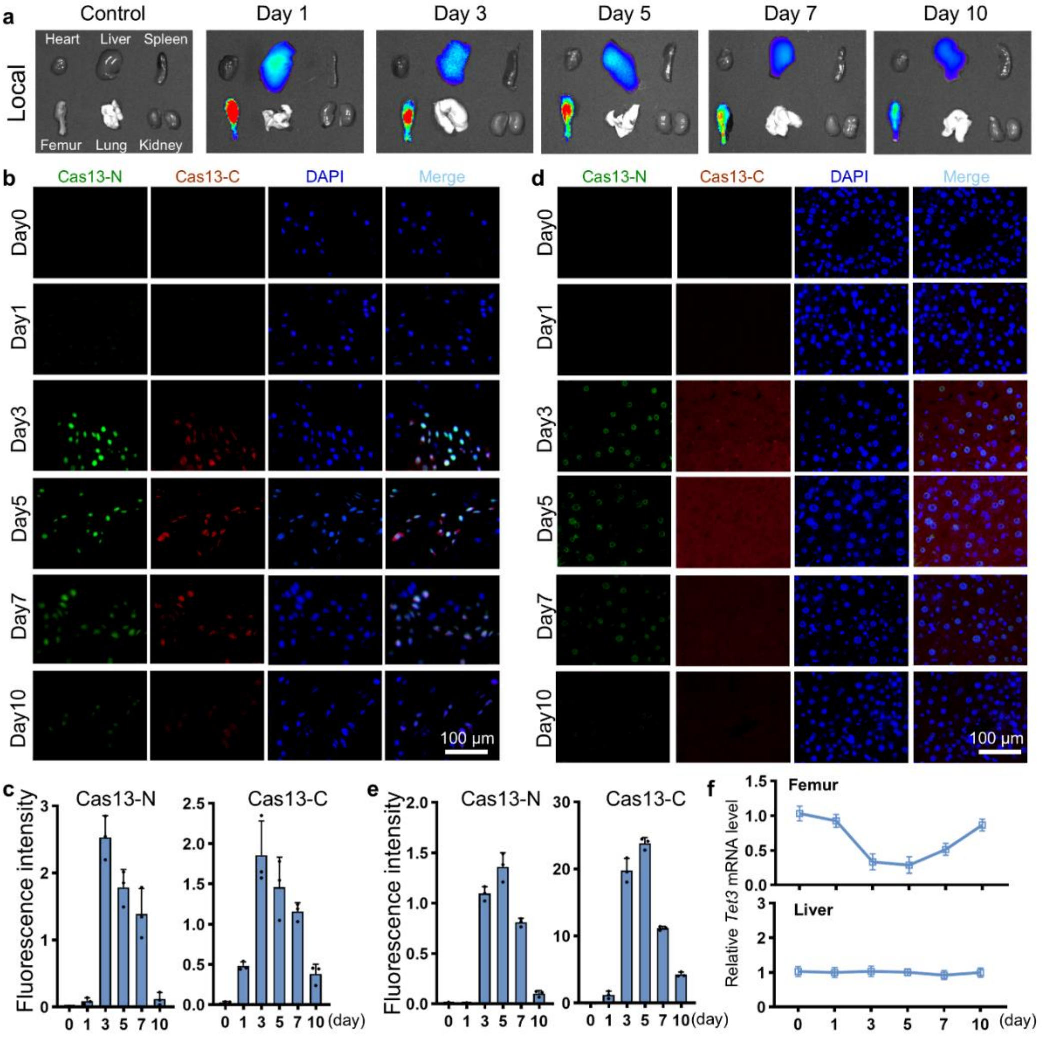

Fig. 5 Pharmacokinetics and biodistribution of UCNPs-PEI@paCas13d following intrafemoral injection

(a) Representative *in vivo* fluorescence imaging showing biodistribution of UCNPs-PEI@paCas13d at Days 1, 3, 5, 7, and 10 following intrafemoral injection. **(b)** Time-course immunofluorescence imaging of split-Cas13d expression in femoral tissue. Cas13d-N (green), Cas13d-C (orange), and nuclei (DAPI, blue) were detected at Days 0, 1, 3, 5, 7, and 10 post-injection. Scale bar = 100 μm. **(c)** Immunofluorescence imaging of Cas13d expression in hepatic tissue at the same time points. Scale bar = 100 μm. **(d)** Quantitative analysis of Cas13d-N and Cas13d-C fluorescence intensity in femoral tissue over time. **(e)** Quantitative analysis of Cas13d-N and Cas13d-C fluorescence intensity in liver tissue. **(f)** Quantitative RT-PCR analysis of *Tet3* mRNA levels in femur and liver tissues over 10 days. Data are mean ± SEM from n=5 animals per timepoint.

Changed in the "Supplementary Information" (Supplementary Fig. 13, Pages

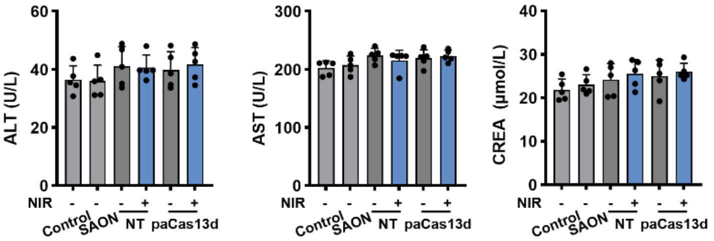

17)

Supplementary Fig.13 Hepatic and renal function safety assessment

Serum biochemical markers evaluating liver and kidney function following UCNPs-PEI@paCas13d treatment. Alanine aminotransferase (ALT), aspartate aminotransferase (AST), and creatinine (CREA) levels remain no significant change across all experimental groups, indicating absence of hepatotoxicity or nephrotoxicity. Data represent mean ± SEM (n=5 animals per group). Statistical analysis by one-way ANOVA showed no significant differences between groups (P > 0.05).

Changed in the "Supplementary Information" (Supplementary Fig. 14, Pages

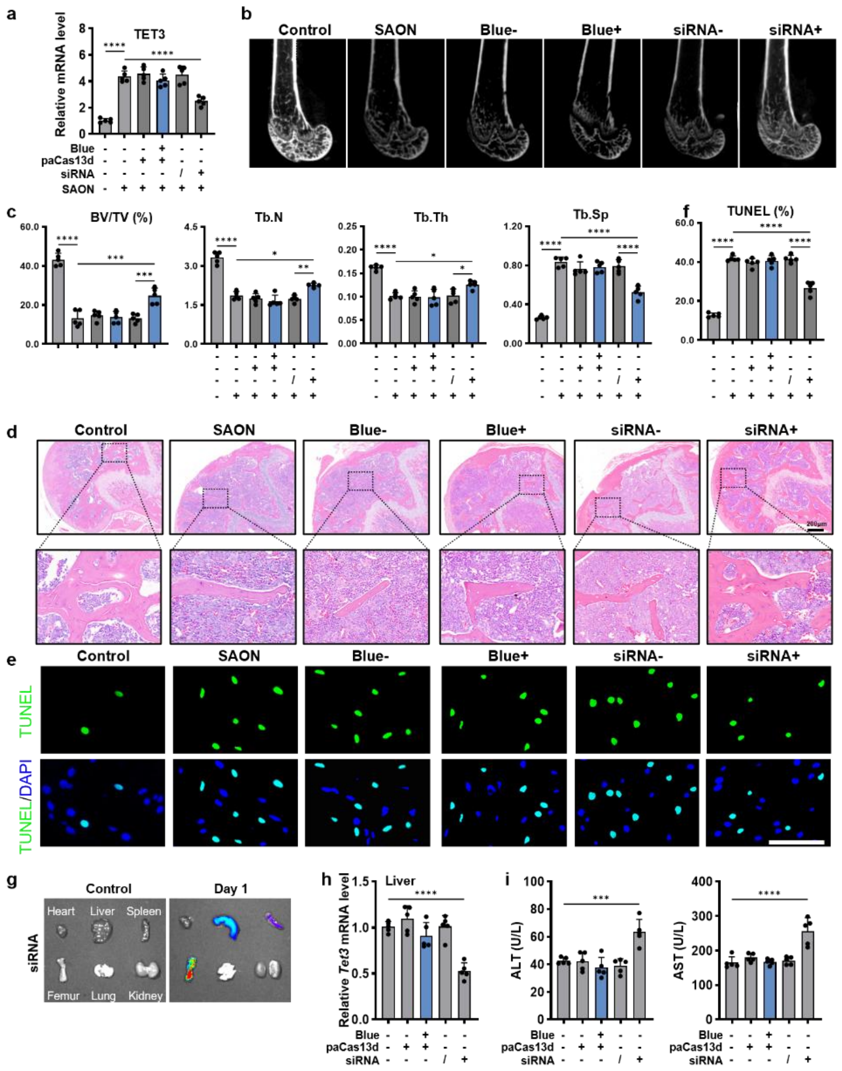

18)

Supplementary Fig.14 Evaluation of blue light activation and siRNA-mediated *Tet3* knockdown in SAON model

(a) Quantitative RT-PCR analysis of femoral *Tet3* mRNA levels across treatment groups, including siRNA-mediated TET3 knockdown and direct blue light activation of paCas13d without UCNPs. /: scrambled siRNA control. (b) Representative micro-CT 3D reconstruction images of femur trabecular bone. (c) Quantitative micro-CT analysis of trabecular bone parameters including BV/TV, Tb.N, Tb.Th, and Tb.Sp. (d) Hematoxylin and eosin (H&E) staining of femur trabecular bone. Upper panels show low magnification overview; lower panels show high magnification of trabecular microarchitecture within boxed regions. Scale bars = 200 μm. (e) Representative TUNEL staining (green) of femoral sections showing apoptotic osteocytes. DAPI (blue) counterstains nuclei. Scale bar, 50 μm. (f) Quantitative analysis of TUNEL-positive osteocytes. (g) *In vivo* organ imaging of siRNA at Day 1 post-injection. (h) Quantitative RT-PCR analysis of hepatic *Tet3* mRNA levels to assess potential off-target gene suppression across treatment groups. (i) Serum alanine aminotransferase (ALT) and aspartate aminotransferase (AST) levels measured as indicators of liver function. Data are mean ± SEM, n=5 animals per group. $^{****}P < 0.0001$, $^{***}P < 0.001$, $^{**}P < 0.01$, $^{*}P < 0.05$.

7. The manuscript does not include a blue light control group to demonstrate the necessity of using UCNPs-PEI for NIR-to-blue light phototransduction. Without this comparison, it is difficult to assess whether the UCNPs-PEI system offers any advantage over direct blue light activation usage *in vivo* with paCAS13d system alone. This should be addressed demonstrated experimentally, for example, using ex vivo tissue explant system.

Response: We thank the reviewer for this critical suggestion. We have now included a direct blue light control group to demonstrate the necessity of UCNPs-PEI-mediated NIR-to-blue light phototransduction. In this control group, UCNPs-PEI@paCas13d was administered via intrafemoral injection followed by direct blue light illumination of the femur (without NIR). As shown in the revised Supplementary Fig.14, the Blue light group failed to achieve therapeutic efficacy despite the presence of both UCNPs-PEI and paCas13d. Specifically, direct blue light exposure resulted in no significant reduction in femoral TET3 expression (Supplementary Fig.14a), no improvement in trabecular bone microarchitecture (Supplementary Fig.14b,c), and no reduction in osteocyte apoptosis (Supplementary Fig.14e,f), with outcomes statistically indistinguishable from the untreated SAON group. In stark contrast, the UCNPs-PEI@paCas13d + NIR group demonstrated robust TET3 suppression and complete therapeutic rescue (Fig. 6). These results directly demonstrate that external blue light cannot penetrate sufficiently through skin and muscle layers to reach the bone, and therefore cannot activate the paCas13d system at the target site. Only through NIR excitation of UCNPs can blue light be generated locally within the deep tissue compartment, enabling effective photoactivation of the optogenetic switch. This comparison unequivocally establishes that the NIR-to-blue phototransduction function of UCNPs-PEI is essential for achieving deep tissue optogenetic control *in vivo*.

Changed in the "Revised Manuscript" (Lines 590-602)

The direct blue light control group, where UCNPs-PEI@paCas13d was delivered via intrafemoral injection followed by transcutaneous blue light illumination (470 nm, 0.002 W/cm^2) instead of NIR, revealed the absolute necessity of upconversion-mediated photoactivation for deep tissue applications. Despite successful nanoparticle and paCas13d delivery to the femur, direct blue light activation failed to achieve any therapeutic benefit. Femoral *Tet3* expression remained elevated at levels comparable to untreated SAON controls (Supplementary Fig.14a), trabecular bone parameters showed no improvement (Supplementary Fig.14b-14d), and osteocyte apoptosis remained unabated (Supplementary Fig.14e and 14f). This complete absence of therapeutic effect demonstrates that external blue light cannot penetrate sufficiently through overlying skin, subcutaneous tissue, and muscle layers (~3-5 mm total depth) to reach the bone

tissues and activate the optogenetic switch. Only through NIR excitation of UCNPs can blue light be generated locally within the deep tissue compartment, enabling effective photoactivation where direct external blue light is physically incapable of reaching.

Changed in the "Supplementary Information" (Supplementary Fig. 14, Pages

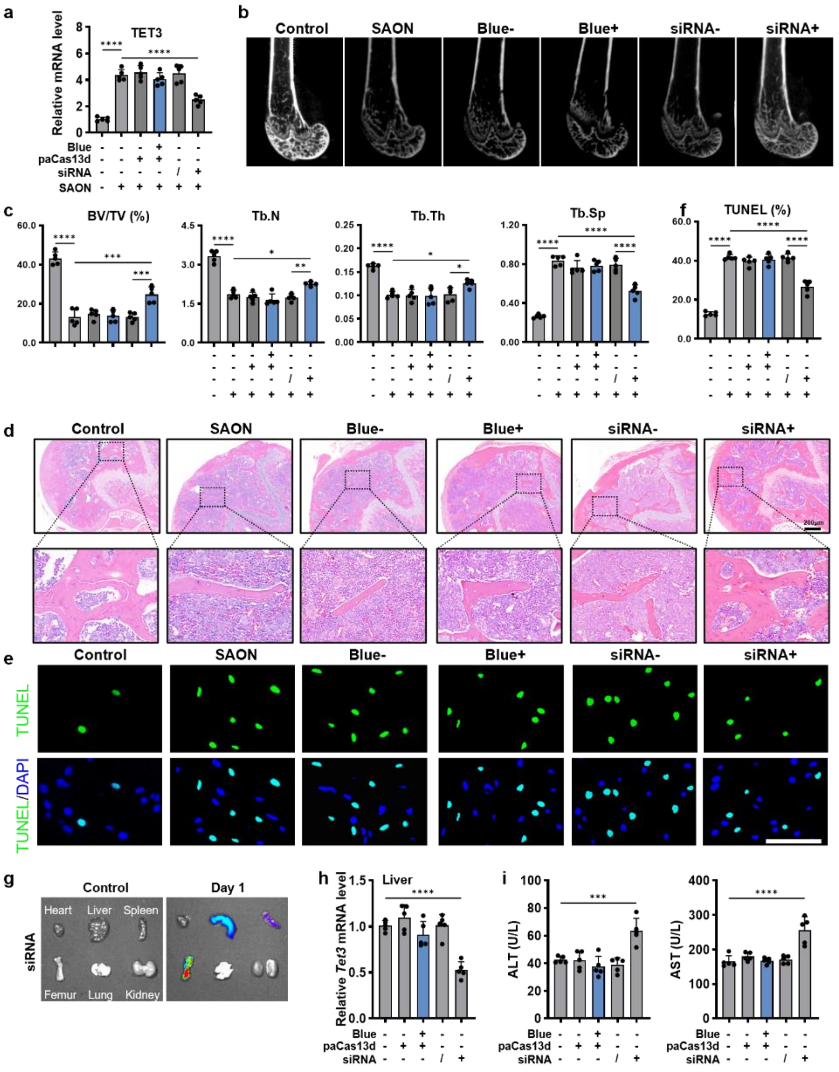

18)

Supplementary Fig.14 Evaluation of blue light activation and siRNA-mediated *Tet3* knockdown in SAON model

(a) Quantitative RT-PCR analysis of femoral *Tet3* mRNA levels across treatment groups, including siRNA-mediated TET3 knockdown and direct blue light activation of paCas13d without UCNPs. / : scrambled siRNA control. (b) Representative micro-CT 3D reconstruction images of femur trabecular bone. (c) Quantitative micro-CT analysis of trabecular bone parameters including BV/TV, Tb.N, Tb.Th, and Tb.Sp. (d) Hematoxylin and eosin (H&E) staining of femur trabecular bone. Upper panels show low magnification overview; lower panels show high magnification of trabecular microarchitecture within boxed regions. Scale bars = 200 μm. (e) Representative TUNEL staining (green) of femoral sections showing apoptotic osteocytes. DAPI (blue)

counterstains nuclei. Scale bar, 50 µm. (f) Quantitative analysis of TUNEL-positive osteocytes. (g) *In vivo* organ imaging of siRNA at Day 1 post-injection. (h) Quantitative RT-PCR analysis of hepatic *Tet3* mRNA levels to assess potential off-target gene suppression across treatment groups. (i) Serum alanine aminotransferase (ALT) and aspartate aminotransferase (AST) levels measured as indicators of liver function. Data are mean ± SEM, n=5 animals per group. $^{****}P < 0.0001$, $^{***}P < 0.001$, $^{**}P < 0.01$, $^{*}P < 0.05$.

8. Did the authors assess the *in vivo* bioavailability of UCNPs-PEI@paCas13d? If not, what rationale supports the decision to administer weekly intrafemoral injections? Clarifying this would help justify the dosing strategy used in the study.

Response: We thank the reviewer for this important question. We did assess the *in vivo* pharmacokinetics and bioavailability of UCNPs-PEI@paCas13d to establish a rational dosing strategy. Following intrafemoral injection, we performed longitudinal *in vivo* fluorescence imaging and molecular analysis over 10 days (Fig. R6). *In vivo* imaging revealed peak UCNPs-PEI signal in the femur at Days 1-5, followed by gradual decline through Day 10 (Fig. R6a). Correspondingly, immunofluorescence analysis of femoral tissue showed robust expression of Cas13d-N and Cas13d-C fragments at Days 3-7, followed by significant reduction by Day 10 (Fig. R6b, R6c). Most importantly, functional assessment showed that femoral *Tet3* mRNA levels remained significantly suppressed through Day 7 but returned toward baseline by Day 10 (Fig. R6f), defining a therapeutic window of approximately 7 days. Based on this pharmacokinetic profile, we implemented weekly intrafemoral injections to maintain sustained TET3 suppression throughout the SAON treatment protocol. This dosing regimen ensures continuous therapeutic coverage while minimizing the frequency of invasive procedures, balancing efficacy with practical feasibility for disease management.

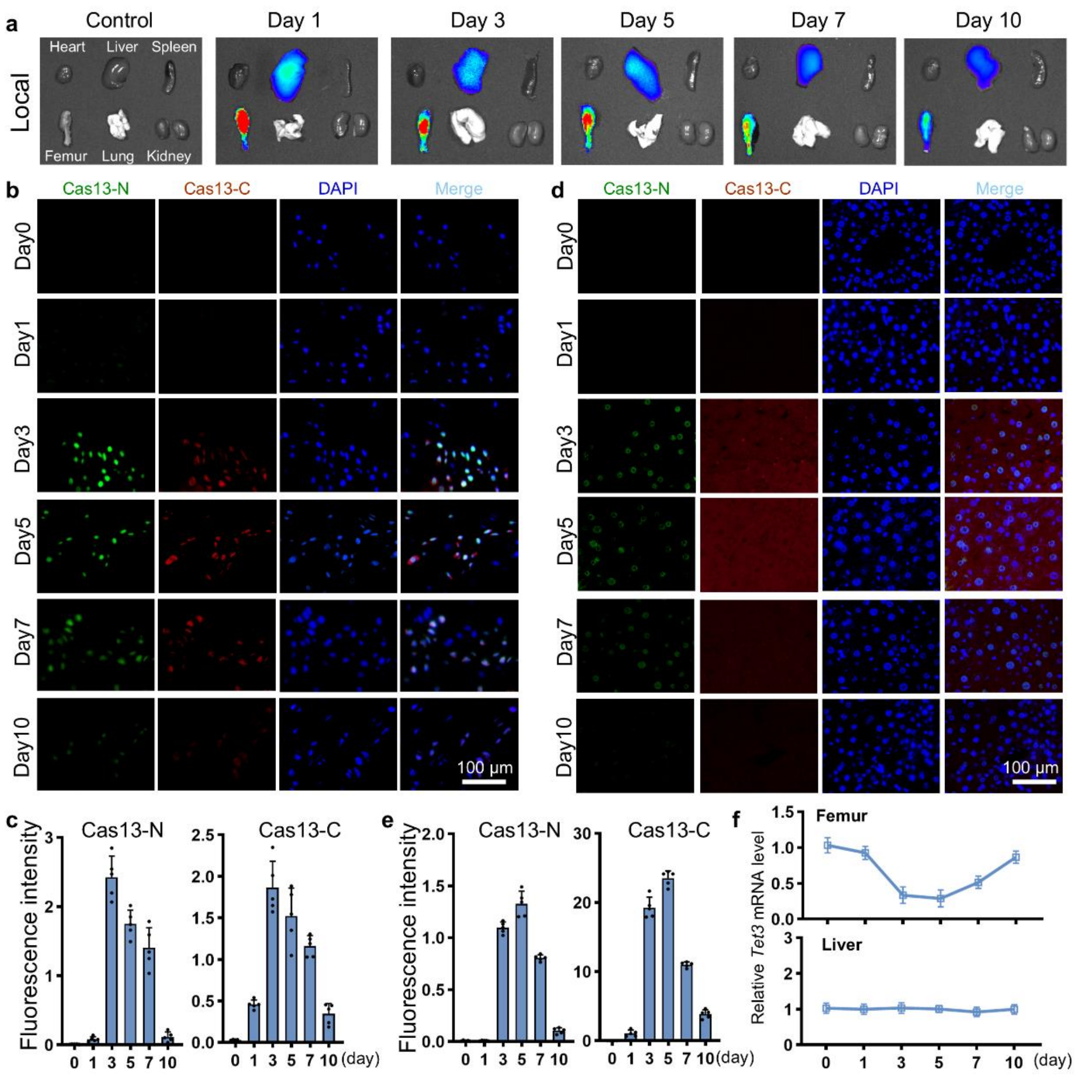

Fig. R6 Pharmacokinetics and biodistribution of UCNPs-PEI@paCas13d following intrafemoral injection

(a) Representative *in vivo* fluorescence imaging showing biodistribution of UCNPs-PEI@paCas13d at Days 1, 3, 5, 7, and 10 following intrafemoral injection. **(b)** Time-course immunofluorescence imaging of split-Cas13d expression in femoral tissue. Cas13d-N (green), Cas13d-C (orange), and nuclei (DAPI, blue) were detected at Days 0, 1, 3, 5, 7, and 10 post-injection. Scale bar = 100 μm. **(c)** Quantitative analysis of Cas13d-N and Cas13d-C fluorescence intensity in femoral tissue over time. **(d)** Immunofluorescence imaging of Cas13d expression in hepatic tissue at the same time points. Scale bar = 100 μm. **(e)** Quantitative analysis of Cas13d-N and Cas13d-C fluorescence intensity in liver tissue. **(f)** Quantitative RT-PCR analysis of *Tet3* mRNA levels in femur and liver tissues over 10 days. Data are mean ± SEM from n=5 animals per timepoint.

Changed in the “Revised Manuscript” (Lines 451-470)

2.6 Pharmacokinetics and biodistribution of UCNPs-PEI@paCas13d *in vivo*

For safe and effective clinical translation, we comprehensively assessed the pharmacokinetics and biodistribution of UCNPs-PEI@paCas13d *in vivo*, to establish a rational dosing regimen and evaluate potential off-target effects. Following intrafemoral injection, longitudinal *in vivo* fluorescence imaging revealed that UCNPs-PEI remained predominantly localized to the injected femur, with peak fluorescence signals observed at Days 1-5 and gradual decline over 10 days (Fig. 5a). Notably, progressive hepatic accumulation was also detected, indicating hepatobiliary clearance as the primary metabolic pathway. To correlate nanoparticle distribution with functional gene delivery, we performed immunofluorescence analysis of femoral and hepatic tissues at multiple time points. In the femur, robust expression of both Cas13d-N and Cas13d-C fragments was observed at Days 3-7, with significant reduction by Day 10 (Fig. 5b, 5c), consistent with nanoparticle clearance and plasmid degradation kinetics. Most importantly, functional assessment revealed that femoral *Tet3* mRNA levels remained significantly suppressed through Day 7 but returned toward baseline by Day 10 (Fig. 5f), defining a therapeutic window of approximately 7 days. Critically, despite detectable Cas13d-N and Cas13d-C expression in hepatic tissue (Fig. 5d, 5e), hepatic *Tet3* mRNA levels remained unchanged throughout the 10-day observation period (Fig. 5f), demonstrating that paCas13d remains functionally inactive in non-illuminated tissues even when successfully delivered. Based on this pharmacokinetic profile, we implemented a weekly intrafemoral injection regimen for the subsequent SAON treatment protocol.

Changed in the "Revised manuscript" (Fig. 5, Pages 23)

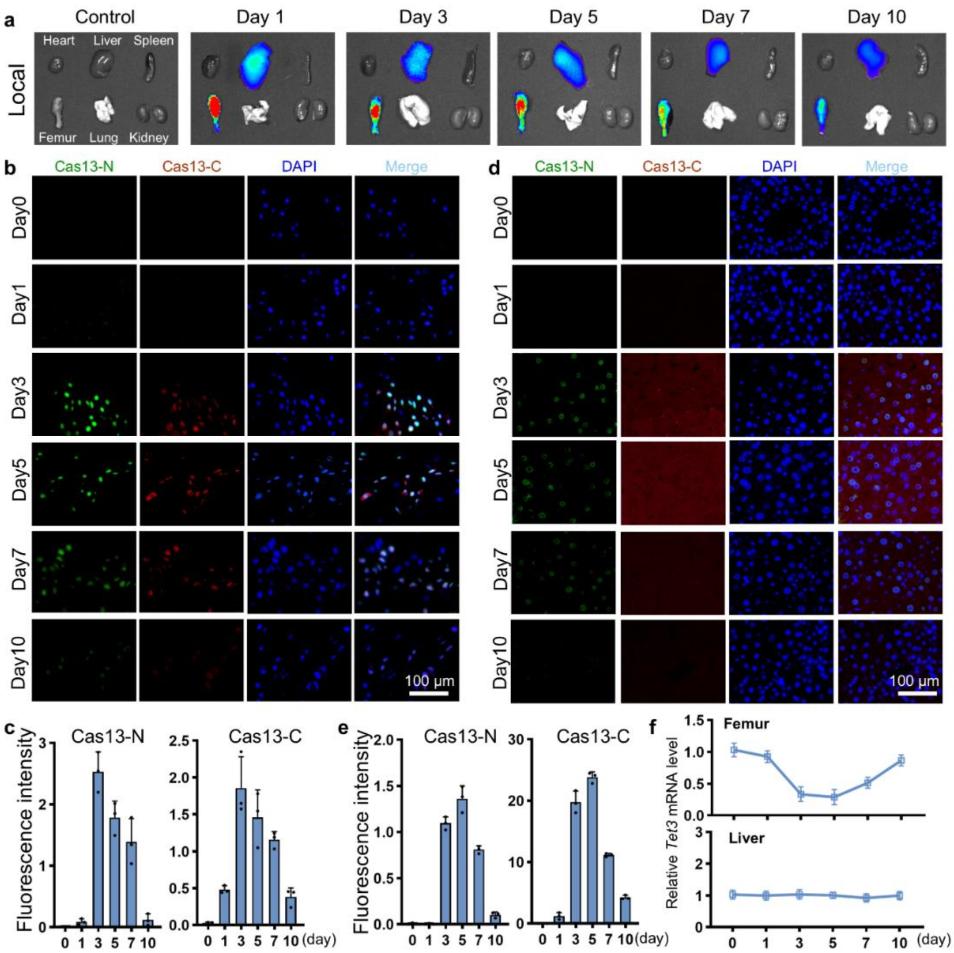

Fig. 5 Pharmacokinetics and biodistribution of UCNPs-PEI@paCas13d following intrafemoral injection

(a) Representative *in vivo* fluorescence imaging showing biodistribution of UCNPs-PEI@paCas13d at Days 1, 3, 5, 7, and 10 following intrafemoral injection. **(b)** Time-course immunofluorescence imaging of split-Cas13d expression in femoral tissue. Cas13d-N (green), Cas13d-C (orange), and nuclei (DAPI, blue) were detected at Days 0, 1, 3, 5, 7, and 10 post-injection. Scale bar = 100 μm. **(c)** Immunofluorescence imaging of Cas13d expression in hepatic tissue at the same time points. Scale bar = 100 μm. **(d)** Quantitative analysis of Cas13d-N and Cas13d-C fluorescence intensity in femoral tissue over time. **(e)** Quantitative analysis of Cas13d-N and Cas13d-C fluorescence intensity in liver tissue. **(f)** Quantitative RT-PCR analysis of *Tet3* mRNA levels in femur and liver tissues over 10 days. Data are mean ± SEM from n=5 animals per timepoint.

9. The authors should explain why intrafemoral injection was performed and necessary versus a systemic injection. How does a localized anatomical delivery pose an advantage versus conventional localized delivery of a siRNA system? Could this be practically used in a clinical setting? This is important since the manuscript title implies a new therapy.

Response: We thank the reviewer for these important questions that address the translational potential of our approach. First, regarding intrafemoral versus systemic delivery: our comparative biodistribution analysis (Fig. R6 and R7) demonstrates that intravenous administration results in predominant hepatic accumulation via RES uptake, with minimal femoral accumulation. This biodistribution pattern renders systemic delivery fundamentally inefficient for bone-targeted therapy. In contrast, intrafemoral injection resulted in UCNPs-PEI remaining predominantly localized to the injected femur over 7 days, achieving high local drug concentration at the disease site. This anatomical targeting is particularly important for SAON, which predominantly affects specific weight-bearing joints while sparing systemic bone, making localized intervention both more effective and safer than systemic approaches that would unnecessarily expose the entire skeleton and other organs.

Second, while both UCNPs-PEI@paCas13d and siRNA can be administered locally, our photoactivatable system offers a critical advantage through light-gated spatial control. As demonstrated in Fig. R11, although both delivery systems achieve comparable therapeutic efficacy in the target femur, siRNA exhibited systemic redistribution to the liver, resulting in unintended hepatic TET3 suppression and hepatotoxicity (Fig. R11h, R11i). This occurs because siRNA is constitutively active - once delivered to any cell, it silences target genes regardless of location. In contrast, UCNPs-PEI@paCas13d, despite showing similar hepatic accumulation (Fig. R11g), produced no hepatic gene suppression and maintained normal liver function (Fig. R11j, R11k). This fundamental difference arises because our system requires both molecular delivery and NIR light activation. Since NIR illumination is applied exclusively to the femoral region, only nanoparticles within the illuminated tissue can activate gene editing, preventing off-target effects even when the delivery vehicle redistributes systemically. This adds an essential layer of spatial selectivity beyond anatomical delivery alone, transforming local delivery from a passive anatomical restriction to an active, optically-controlled therapeutic intervention.

Regarding clinical feasibility, our approach is highly practical for SAON prevention. The target patient population - individuals requiring prolonged high-dose glucocorticoid therapy (e.g., organ transplant recipients, autoimmune disease patients) - represents a well-defined, high-risk cohort where prophylactic intervention is justified.

The intrafemoral injection procedure is minimally invasive and routinely performed in clinical practice for bone marrow aspiration or intraosseous access, requiring only local anesthesia.[15] NIR illumination (980 nm) is non-invasive, painless, and can be delivered transcutaneously using portable LED arrays, making treatment highly feasible.[6, 16] Importantly, this localized approach preserves the essential systemic anti-inflammatory and immunosuppressive effects of glucocorticoids while preventing skeletal complications - a therapeutic window impossible to achieve with systemic SAON treatments. The photoactivatable design also provides an inherent safety mechanism: if adverse effects occur, therapy can be immediately terminated by withholding light activation, unlike constitutively active gene therapies. These practical advantages, combined with the superior safety profile demonstrated in our preclinical studies, position this technology as a clinically viable strategy for targeted orthopedic gene therapy, with potential applications extending beyond SAON to other localized musculoskeletal disorders requiring spatially restricted RNA modulation.

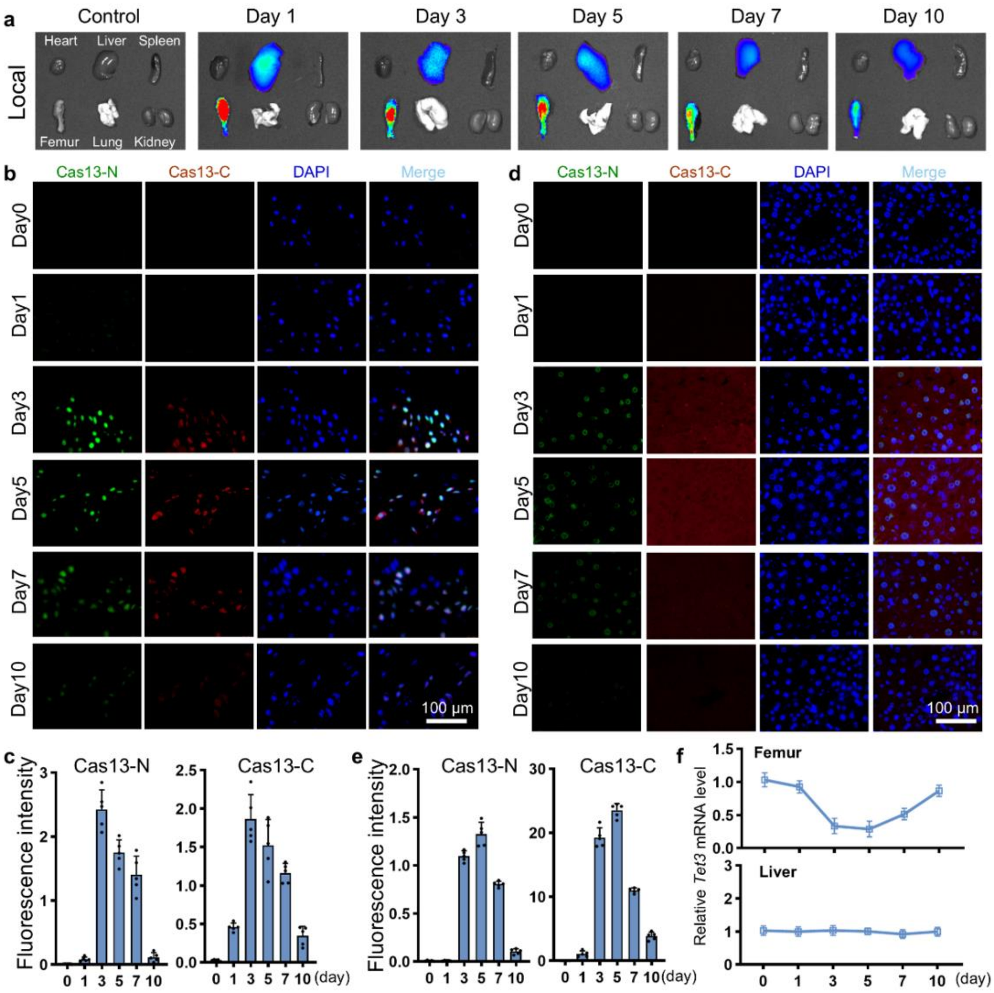

Fig. R6 Pharmacokinetics and biodistribution of UCNPs-PEI@paCas13d following intrafemoral injection

(a) Representative *in vivo* fluorescence imaging showing biodistribution of UCNPs-PEI@paCas13d at Days 1, 3, 5, 7, and 10 following intrafemoral injection. **(b)** Time-course immunofluorescence imaging of split-Cas13d expression in femoral tissue. Cas13d-N (green), Cas13d-C (orange), and nuclei (DAPI, blue) were detected at Days 0, 1, 3, 5, 7, and 10 post-injection. Scale bar = 100 µm. **(c)** Quantitative analysis of Cas13d-N and Cas13d-C fluorescence intensity in femoral tissue over time. **(d)** Immunofluorescence imaging of Cas13d expression in hepatic tissue at the same time points. Scale bar = 100 µm. **(e)** Quantitative analysis of Cas13d-N and Cas13d-C fluorescence intensity in liver tissue. **(f)** Quantitative RT-PCR analysis of *Tet3* mRNA levels in femur and liver tissues over 10 days. Data are mean ± SEM from n=5 animals per timepoint.

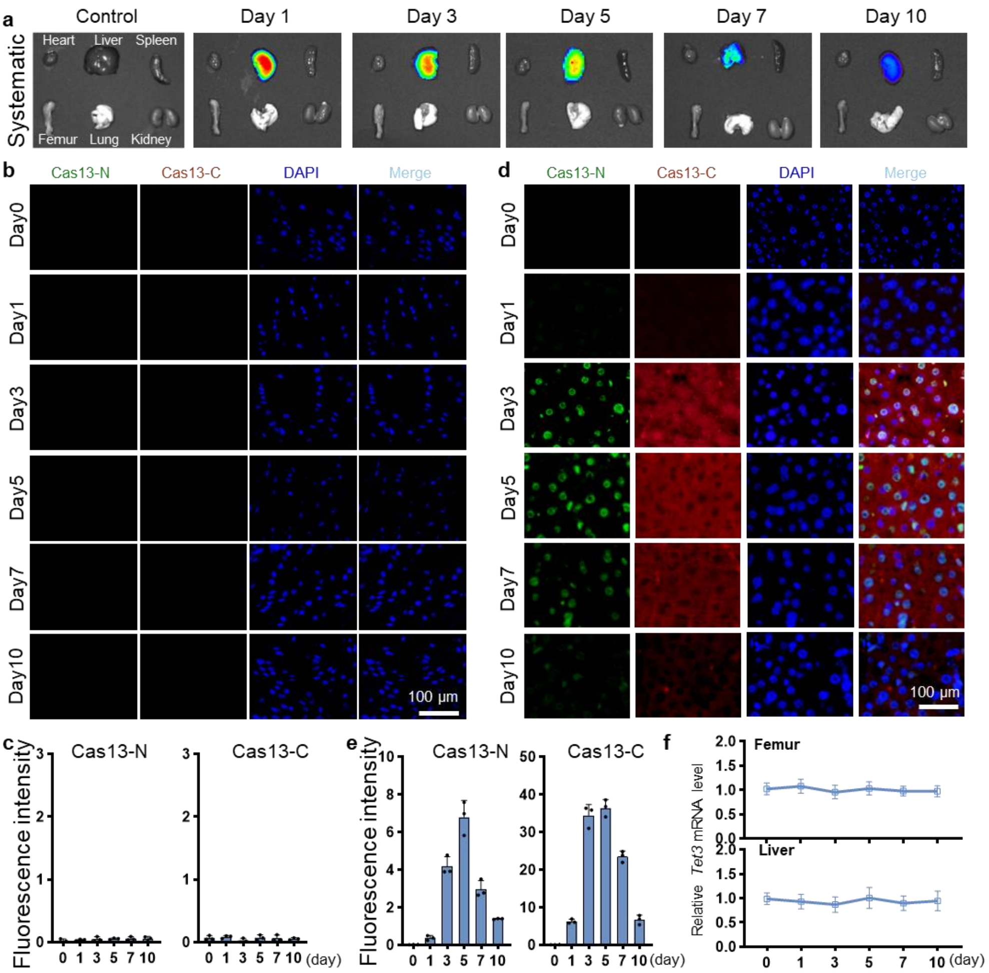

Fig. R7 Pharmacokinetics and biodistribution of UCNPs-PEI@paCas13d following systemic administration

(a) Representative *in vivo* imaging (heart, liver, spleen, femur, lung, kidney) following intravenous injection of UCNPs-PEI@paCas13d at Days 1, 3, 5, 7, and 10 post-injection. **(b,d)** Immunofluorescence staining of Cas13d-N (green) and Cas13d-C (red) in femoral bone tissue **(b)** and hepatic tissue **(d)** at indicated time points following intravenous administration. DAPI (blue) indicates nuclei. Scale bars, 100 µm. **(c,e)** Quantification of Cas13d-N and Cas13d-C fluorescence intensity in femoral **(c)** and

hepatic **(e)** tissues over time. Data show mean fluorescence intensity in arbitrary units per timepoint across five animals. **(f)** Quantitative RT-PCR analysis of *Tet3* mRNA levels in femoral and hepatic tissues following intravenous administration. Data are mean ± SEM from n=5 animals per timepoint.

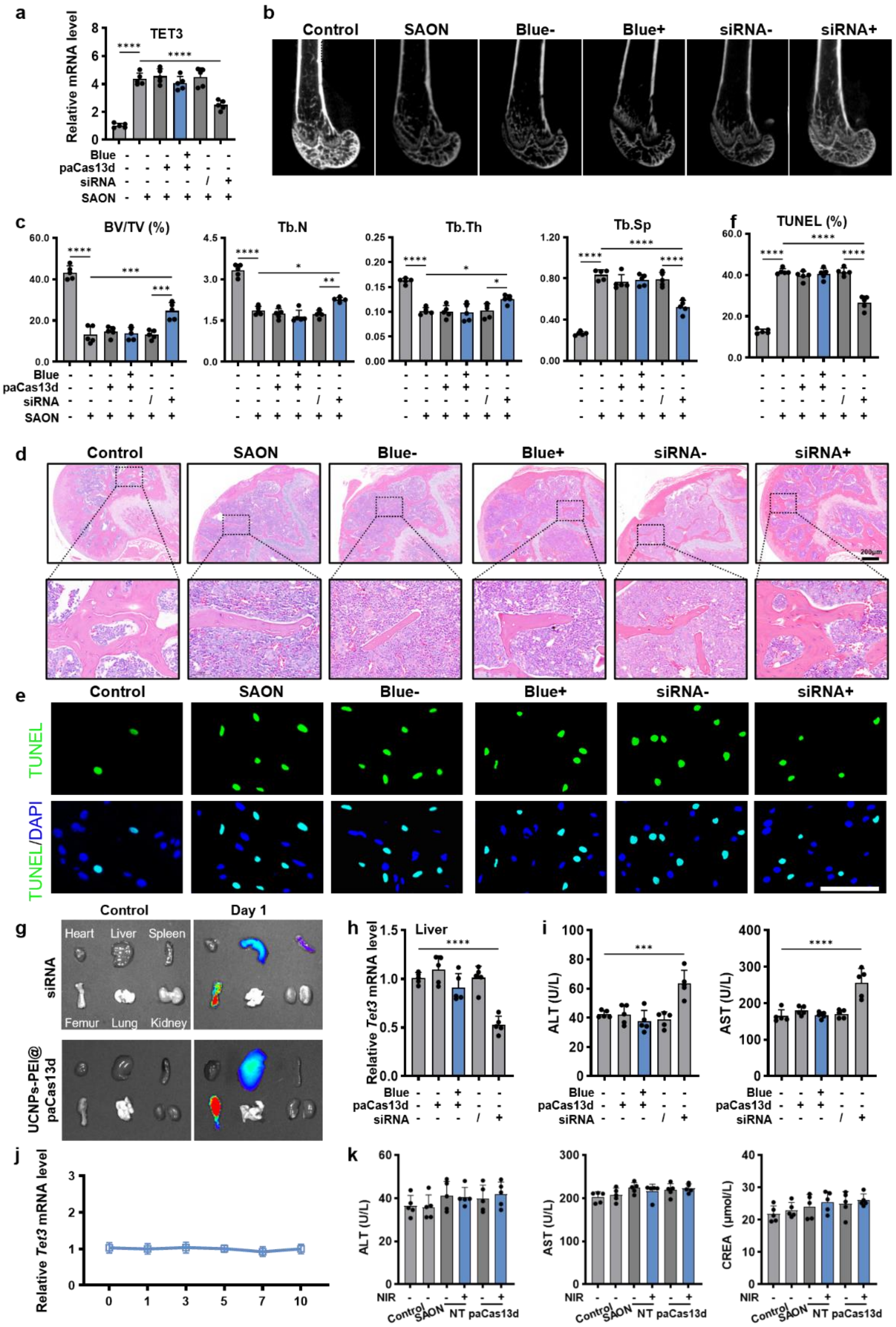

Fig. R11 Evaluation of blue light activation and siRNA-mediated *Tet3* knockdown in SAON model

(a) Quantitative RT-PCR analysis of femoral *Tet3* mRNA levels across treatment groups, including siRNA-mediated TET3 knockdown and direct blue light activation of paCas13d without UCNPs. / : scrambled siRNA control. **(b)** Representative micro-CT 3D reconstruction images of femur trabecular bone. **(c)** Quantitative micro-CT analysis of trabecular bone parameters including BV/TV, Tb.N, Tb.Th, and Tb.Sp. **(d)** Hematoxylin and eosin (H&E) staining of femur trabecular bone. Upper panels show low magnification overview; lower panels show high magnification of trabecular microarchitecture within boxed regions. Scale bars = 200 μm. **(e)** Representative TUNEL staining (green) of femoral sections showing apoptotic osteocytes. DAPI (blue) counterstains nuclei. Scale bar, 50 μm. **(f)** Quantitative analysis of TUNEL-positive osteocytes. **(g)** *In vivo* organ imaging of UCNPs-PEI@paCas13d and siRNA at Day 1 post-injection. **(h)** Quantitative RT-PCR analysis of hepatic *Tet3* mRNA levels to assess potential off-target gene suppression across treatment groups. **(i)** Serum alanine aminotransferase (ALT) and aspartate aminotransferase (AST) levels measured as indicators of liver function. **(j)** Quantitative RT-PCR analysis of *Tet3* mRNA levels in liver tissues over 10 days following intrafemoral administration of UCNPs-PEI@paCas13d. **(k)** Serum biochemical markers evaluating liver and kidney function following UCNPs-PEI@paCas13d treatment. Data are mean ± SEM, n=5 animals per group. $^{****}P < 0.0001$, $^{***}P < 0.001$, $^{**}P < 0.01$, $^{*}P < 0.05$.

Changed in the "Revised manuscript" (Lines 451-488)

2.6 Pharmacokinetics and biodistribution of UCNPs-PEI@paCas13d *in vivo*

For safe and effective clinical translation, we comprehensively assessed the pharmacokinetics and biodistribution of UCNPs-PEI@paCas13d *in vivo*, to establish a rational dosing regimen and evaluate potential off-target effects. Following intrafemoral injection, longitudinal *in vivo* fluorescence imaging revealed that UCNPs-PEI remained predominantly localized to the injected femur, with peak fluorescence signals observed at Days 1-5 and gradual decline over 10 days (Fig. 5a). Notably, progressive hepatic accumulation was also detected, indicating hepatobiliary clearance as the primary metabolic pathway. To correlate nanoparticle distribution with functional gene delivery, we performed immunofluorescence analysis of femoral and hepatic tissues at multiple time points. In the femur, robust expression of both Cas13d-N and Cas13d-C fragments was observed at Days 3-7, with significant reduction by Day 10 (Fig. 5b, 5c), consistent with nanoparticle clearance and plasmid degradation kinetics. Most importantly, functional assessment revealed that femoral *Tet3* mRNA levels remained significantly suppressed through Day 7 but returned toward baseline by Day 10 (Fig. 5f), defining a therapeutic window of approximately 7 days. Critically, despite detectable Cas13d-N and Cas13d-C expression in hepatic tissue (Fig. 5d, 5e), hepatic *Tet3* mRNA levels remained unchanged throughout the 10-day observation period (Fig. 5f), demonstrating that paCas13d remains functionally inactive in non-illuminated tissues even when successfully delivered. Based on this pharmacokinetic profile, we

implemented a weekly intrafemoral injection regimen for the subsequent SAON treatment protocol.

To directly compare delivery routes and further validate the necessity of local administration, we performed parallel biodistribution analysis following intravenous injection of UCNPs-PEI@paCas13d. As expected for nanoparticles in this size range (~200 nm), systemic administration resulted in rapid and predominant hepatic sequestration via reticuloendothelial system uptake, with peak signals at Days 1-5 and gradual clearance by Day 10 (Supplementary Fig. 8a). In stark contrast to intrafemoral injection, femoral accumulation following intravenous delivery was minimal, with correspondingly negligible Cas13d expression in femoral tissue (Supplementary Fig. 8b and 8c), rendering systemic delivery fundamentally inefficient for bone-targeted applications. Immunofluorescence analysis revealed transient hepatic Cas13d expression at Days 3-7 following intravenous administration (Supplementary Fig. 8d and 8e), yet hepatic *Tet3* mRNA levels remained unchanged (Supplementary Fig. 8f), indicating negligible functional gene silencing despite substantial nanoparticle accumulation. This critical observation demonstrates that the requirement for NIR light activation provides spatial confinement of therapeutic activity—even when nanoparticles distribute systemically, gene editing occurs exclusively in illuminated tissues. These pharmacokinetic studies established the rationale for weekly intrafemoral dosing and confirmed light-gated spatial specificity. We therefore proceeded to evaluate therapeutic efficacy in a murine model of SAON.

Changed in the “Revised Manuscript” (Lines 573-589)

To further establish the necessity of UCNPs-mediated NIR activation and benchmark therapeutic efficacy *in vivo*, we evaluated two critical controls in the SAON model. We compared siRNA-mediated *Tet3* knockdown against our photoactivatable system and tested whether direct blue light could activate UCNPs-PEI@paCas13d in deep bone tissue without upconversion. Both controls revealed fundamental limitations that our photoactivatable nanoparticle system overcomes. The siRNA control group, administered via intrafemoral injection, achieved comparable therapeutic efficacy in the target femur, demonstrating significant *Tet3* suppression (Supplementary Fig. 14a), preservation of trabecular bone microarchitecture (Supplementary Fig. 14b-14d), and reduction in osteocyte apoptosis (Supplementary Fig. 14e and 14f). However, despite local administration, siRNA exhibited systemic redistribution to the liver, as evidenced by biodistribution imaging (Supplementary Fig. 14g). Critically, this hepatic accumulation resulted in unintended off-target *Tet3* suppression (Supplementary Fig. 14h) and significant hepatotoxicity, with elevated serum transaminases (Supplementary Fig. 14i). In stark contrast, the UCNPs-PEI@paCas13d+NIR group, despite similar

hepatic nanoparticle accumulation, showed no hepatic *Tet3* suppression and maintained normal liver function (Supplementary Fig. 13), demonstrating that light-gated spatial control prevents off-target gene editing in non-illuminated tissues.

Changed in the "Revised manuscript" (Lines 663-673)

Regarding the clinical feasibility of our delivery and photoactivation strategy, both components employ established clinical procedures. Intrafemoral injection is routinely performed for bone marrow aspiration under local anesthesia with minimal invasiveness.[15] NIR illumination can be delivered transcutaneously using portable LED devices approved for clinical use, enabling non-invasive, painless activation in outpatient settings.[16] The light-gated control mechanism provides clinical advantages through spatial precision via targeted illumination and temporal control via on-demand activation, with the ability to immediately terminate therapy by withholding light if needed. Beyond SAON, the modular platform architecture enables rapid adaptation to diverse localized musculoskeletal disorders including osteoarthritis, fracture healing, and degenerative disc disease, positioning this technology as a clinically viable strategy for targeted orthopedic gene therapy.

References

1.Kennedy MJ, Hughes RM, Peteya LA, Schwartz JW, Ehlers MD, Tucker CL. Rapid blue-light-mediated induction of protein interactions in living cells. *Nature methods* **7**, 973-975 (2010).

2.Taslimi A*, et al.* An optimized optogenetic clustering tool for probing protein interaction and function. *Nature communications* **5**, 4925 (2014).

3.Zetsche B, Volz SE, Zhang F. A split-Cas9 architecture for inducible genome editing and transcription modulation. *Nat Biotechnol* **33**, 139-142 (2015).

4.Bogdan N, Vetrone F, Ozin GA, Capobianco JA. Synthesis of ligand-free colloidally stable water dispersible brightly luminescent lanthanide-doped upconverting nanoparticles. *Nano letters* **11**, 835-840 (2011).

5.Zhou J, Liu Q, Feng W, Sun Y, Li F. Upconversion luminescent materials: advances and applications. *Chem Rev* **115**, 395-465 (2015).

6.Zhao J, Li B, Ma J, Jin W, Ma X. Photoactivatable RNA N(6) -Methyladenosine Editing with CRISPR-Cas13. *Small* **16**, e1907301 (2020).

7.Kong W*, et al.* A General Strategy for Ligand Exchange on Upconversion Nanoparticles. *Inorg Chem* **56**, 872-877 (2017).

8.Zhao L, Kutikov A, Shen J, Duan C, Song J, Han G. Stem cell labeling using polyethylenimine conjugated (alpha-NaYbF4:Tm3+)/CaF2 upconversion nanoparticles. *Theranostics* **3**, 249-257 (2013).

9.Kaarjel K. Narayanasamy JCP, Marwan Merkhan, Ajile Elttayef, Jon Dobson, Neil D. Telling. Cytotoxic effect of PEI-coated magnetic nanoparticles on the regulation of cellular focal adhesions and actin stress fibres. *Materialia* **13**, 100848 (2020).

10.Lai WF, Rogach AL, Wong WT. Molecular design of upconversion nanoparticles for gene delivery. *Chemical science* **8**, 7339-7358 (2017).

11.Casper J, Schenk SH, Parhizkar E, Detampel P, Dehshahri A, Huwyler J. Polyethylenimine (PEI) in gene therapy: Current status and clinical applications. *Journal of controlled release : official journal of the Controlled Release Society* **362**, 667-691 (2023).

12.Wang GD, Tan YZ, Wang HJ, Zhou P. Autophagy promotes degradation of polyethyleneimine-alginate nanoparticles in endothelial progenitor cells. *Int J Nanomedicine* **12**, 6661-6675 (2017).

13.Raj EN, Lin YW, Chen CH, Liu KK, Chao JI. Selective Autophagy Pathway of Nanoparticles and Nanodrugs: Drug Delivery and Pathophysiological Effects. *Advanced Therapeutics* **3**, (2020).

14.Wessels HH, Mendez-Mancilla A, Guo X, Legut M, Daniloski Z, Sanjana NE. Massively parallel Cas13 screens reveal principles for guide RNA design. *Nat Biotechnol* **38**, 722-727 (2020).

15.Evans CH. Gene delivery to bone. *Advanced drug delivery reviews* **64**, 1331-1340 (2012).

16.Zou Q*, et al.* Photoactivatable base editors for spatiotemporally controlled genome editing in vivo. *Biomaterials* **302**, 122328 (2023).

Our Replies to the Reviewer's Comments and the Revisions Made to the Manuscript

Reviewer #1 (Remarks to the Author):

The authors' response to my previous comments/questions was largely satisfactory. However, two new significant concerns have arisen based on the newly added experimental data, as outlined below. Therefore, unfortunately, I cannot recommend publication of the paper as it stands. A proper explanation based on solid experimental evidence is required for these two points of concern.

(1) Figure R1 (Supplementary Fig. 1) demonstrates that CRY2PHR-mCherry rapidly accumulates in the nucleus within one minute after 470 nm light exposure, via heterodimerization with the nuclear-localized protein CIBN. Conversely, upon cessation of light exposure, nuclear CRY2PHR-mCherry gradually decreased over 30 minutes, returning to baseline levels of cytoplasmic distribution. While these results indirectly demonstrate CRY2PHR-mCherry heterodimerization with CIBN, they raise new questions. Specifically, the following three points:

(i) If the CRY2PHR/CIBN heterodimer transported into the nucleus gradually dissociates in the dark, why does CRY2PHR-mCherry not persist in the nucleus?

(ii) If dissociation does not occur, does the heterodimer persist indefinitely within the nucleus?

(iii) By what mechanism is CRY2PHR-mCherry or the CRY2PHR/CIBN heterodimer expelled from the nucleus?

There is no explanation based on experimental evidence for these points, and it cannot be asserted that reversibility has been demonstrated.

Response: We thank the reviewer for these insightful mechanistic questions regarding the reversibility of CRY2PHR/CIBN heterodimerization and nuclear translocation. The observed reversibility is achieved through a carefully engineered balance of nuclear localization and nuclear export signals in our construct design, a strategy that has been successfully employed in other inducible protein systems such as the rapamycin-inducible split-Cas9 (Fig. R1).[1] Specifically, CIBN-GFP contains dual nuclear localization signals (NLS) fused at both N- and C-termini creating a strong nuclear import bias, while CRY2PHR-mCherry contains a single nuclear export signal (NES) at the C-terminus directing cytoplasmic localization. In darkness, CIBN-GFP accumulates in the nucleus driven by its dual NLS, while CRY2PHR-mCherry remains predominantly cytoplasmic due to active nuclear export via the NES. Upon 470 nm illumination, CRY2PHR binds to CIBN with high affinity, and the resulting

heterodimer translocates into the nucleus because the dual NLS on CIBN dominates over the single NES on CRY2PHR, shifting the nucleocytoplasmic equilibrium toward nuclear accumulation (Fig. R2a).

(i) Why does CRY2PHR-mCherry not persist in the nucleus after dark-induced dissociation? CRY2PHR-mCherry does not persist in the nucleus because it possesses an active nuclear export signal (NES) that continuously mediates CRM1-dependent nuclear export.[2] Upon dissociation from CIBN in darkness, free CRY2PHR-mCherry-NES is no longer tethered to the nuclear-localized CIBN and is actively transported out of the nucleus back to the cytoplasm through the NES-mediated export machinery.

(ii) Would the heterodimer persist indefinitely if dissociation did not occur? Yes, if the CRY2PHR/CIBN heterodimer were stable and did not dissociate in darkness, it would indeed persist in the nucleus indefinitely due to the dominant dual NLS on CIBN. The single NES on CRY2PHR is insufficient to overcome the nuclear import bias of CIBN's dual NLS when the two proteins are bound together. However, the CRY2PHR/CIBN interaction is inherently reversible and light-dependent. The molecular basis for this reversibility lies in the photochemistry of CRY2PHR's FAD cofactor: blue light photoreduction of FAD triggers conformational changes that enable CIBN binding, while in darkness the FAD spontaneously reoxidizes, eliminating the conformational state required for CIBN interaction. This dark-state reversion occurs with a half-life of approximately 10-15 minutes, as extensively characterized by Kennedy et al. and Taslimi et al.[3, 4] Our experimental data directly demonstrate this reversibility, as nuclear CRY2PHR-mCherry gradually dissipates over 30 minutes following light cessation (Fig. R2b, c), with kinetics consistent with these published dissociation parameters.

(iii) By what mechanism is CRY2PHR-mCherry expelled from the nucleus? The nuclear expulsion of CRY2PHR-mCherry occurs through two sequential mechanistic steps. First, upon light cessation, the CRY2PHR/CIBN heterodimer undergoes spontaneous dissociation as the FAD cofactor in CRY2PHR reverts from its light-activated reduced state to the oxidized ground state, eliminating the conformational change required for CIBN binding. Second, once the proteins dissociate within the nucleus, free CRY2PHR-mCherry is then actively exported via its NES through the CRM1 nuclear export pathway. This NES-mediated transport is a continuous, energy-dependent process that moves CRY2PHR-mCherry from the nucleus to the cytoplasm. Meanwhile, CIBN-GFP remains nuclear due to its dual NLS and undergoes continuous nuclear reimport if any leakage to the cytoplasm occurs. This dual-step mechanism—dark-induced dissociation followed by NES-mediated active export—ensures complete

reversibility and maintains the spatial segregation of the two proteins in the absence of light stimulation.

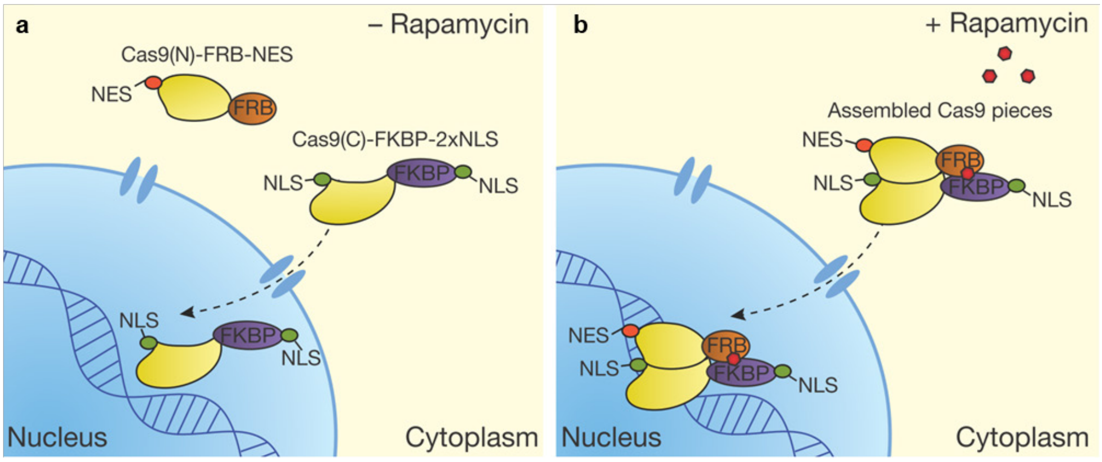

Fig. R1 Schematic illustration of the split-Cas9 system (adapted from Zetsche et al., 2015)[1]

(**a**) In the absence of rapamycin, Cas9(N)-FRB-NES is retained in the cytoplasm by its nuclear export signal (NES), while Cas9(C)-FKBP-2×NLS localizes to the nucleus via dual nuclear localization signals. (**b**) Upon rapamycin addition, FRB and FKBP heterodimerize, and the assembled Cas9 complex translocates to the nucleus driven by the dominant dual NLS, overriding the single NES. This design principle—balancing NES-mediated cytoplasmic retention against NLS-driven nuclear import—is directly analogous to our NLS-CIBN-GFP-NLS and CRY2PHR-mCherry-NES system, where light replaces rapamycin as the dimerization trigger.

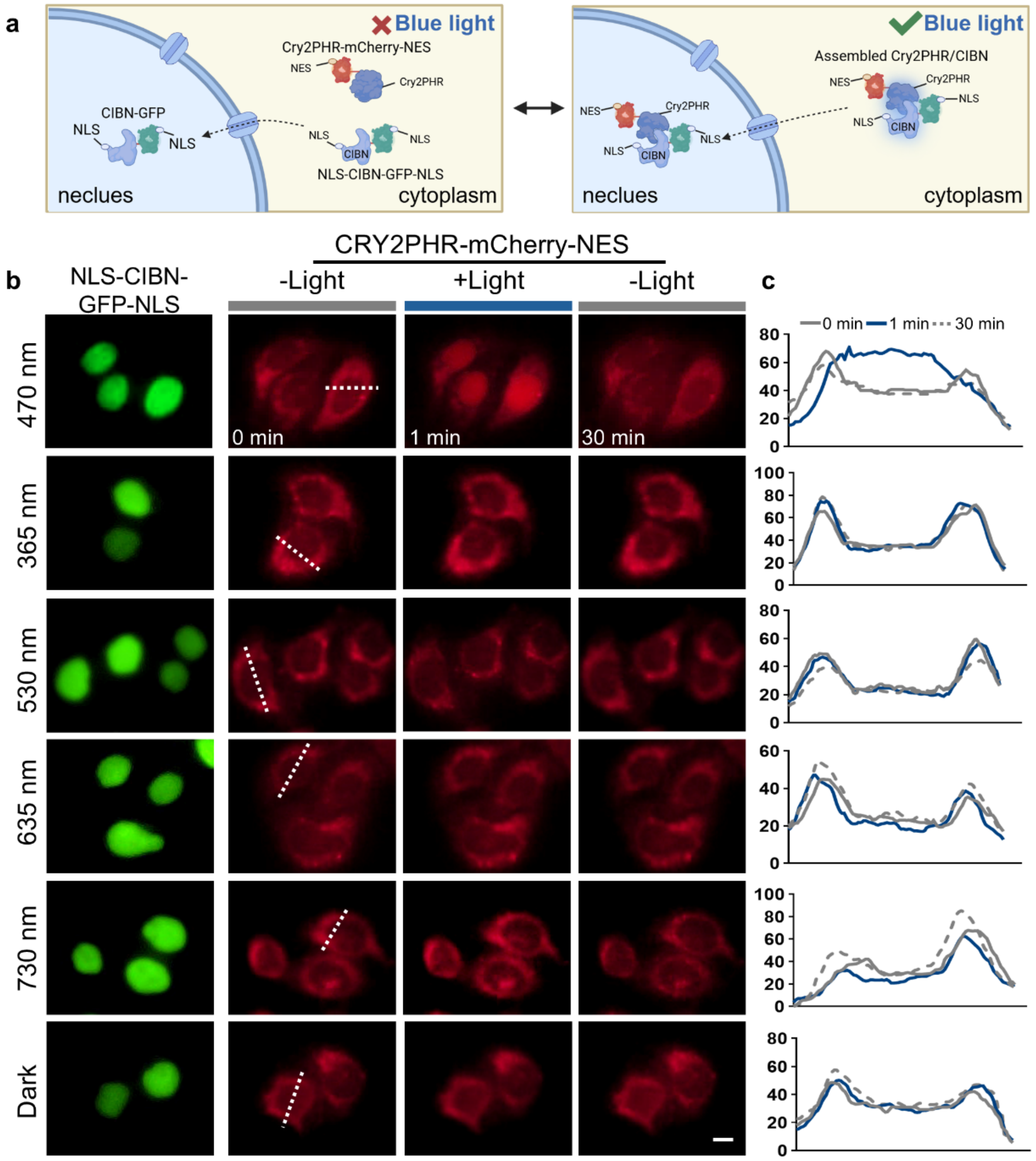

Fig. R2 Wavelength specificity and reversibility of CRY2PHR/CIBN light-inducible dimerization

(a) Schematic illustration of the nucleus-cytoplasm translocation assay. Nuclear-localized NLS-CIBN-GFP-NLS (green) and cytoplasmic CRY2PHR-mCherry-NES (red) undergo heterodimerization upon blue light (470 nm) illumination, inducing CRY2PHR-mCherry nuclear translocation. NLS: nuclear localization signal; NES: nuclear export signal. **(b)** Representative live-cell imaging of HEK293T cells expressing NLS-CIBN-GFP-NLS and CRY2PHR-mCherry-NES exposed to different wavelengths (470 nm, 365 nm, 530 nm, 635 nm, 730 nm) or maintained in darkness (Dark). Left column: NLS-CIBN-GFP-NLS nuclear localization. Middle columns: CRY2PHR-mCherry distribution before illumination (−Light, 0 min), 1 minute after light onset (+Light, 1 min), and 30 minutes after light cessation (−Light, 30 min). Scale bar, 10 μm. **(c)** Quantitative line scan analysis of CRY2PHR-mCherry fluorescence intensity over time for each wavelength condition shown in **(b)**.

Changed in the "Revised manuscript" (Lines 132-155):

Then we implemented the well-characterized CRY2PHR/CIBN blue light-inducible dimerization system to control split Cas13d reconstitution (Fig. 1c). The molecular mechanism underlying this optogenetic switch has been extensively established: the CRY2PHR photolyase homology region contains a flavin adenine dinucleotide (FAD) chromophore that undergoes photoreduction upon blue light absorption, triggering a conformational change from a closed, CIBN-inaccessible state to an open conformation that exposes the CIBN-binding interface and enables high-affinity heterodimer formation (Kd ~1 μM).[3, 4] Upon light withdrawal, the FAD chromophore spontaneously re-oxidizes in the dark, reverting CRY2PHR to its closed conformation and causing dimer dissociation. This reversible photocycle enables light-gated spatial approximation of split Cas13d fragments, restoring catalytic activity exclusively under illumination while maintaining inactive separation in darkness. To directly validate the functional operation of this light-dependent interaction, we performed nucleus-cytoplasm translocation assays that exploit competing nuclear localization and export signals to visualize heterodimerization dynamics. This design strategy, analogous to the inducible split-Cas9 system,[1] employs CIBN-GFP containing dual nuclear localization signals (NLS) at both termini to ensure strong nuclear accumulation, while CRY2PHR-mCherry contains a single nuclear export signal (NES) directing cytoplasmic retention (Supplementary Fig. 1a). In darkness, the two proteins remain spatially segregated with CIBN-GFP nuclear and CRY2PHR-mCherry cytoplasmic. Upon 470 nm illumination, CRY2PHR-mCherry rapidly accumulated in the nucleus within 1 minute, demonstrating robust light-induced translocation (Supplementary Fig. 1b). This nuclear accumulation occurs because heterodimerization with CIBN creates a complex in which the nuclear import activity mediated by CIBN's dual NLS predominates over the nuclear export activity conferred by CRY2PHR's single NES, driving net nuclear translocation of the heterodimer. Critically, other wavelengths (365, 530, 635, 730 nm) outside the blue light absorption range elicited no response, demonstrating strict wavelength specificity consistent with the FAD chromophore absorption profile. Upon light cessation, nuclear CRY2PHR-mCherry gradually decreased over 30 minutes, returning to baseline cytoplasmic distribution and demonstrating complete reversibility (Supplementary Fig. 1b, c). This reversible nuclear clearance occurs through a two-step mechanism: the CRY2PHR/CIBN heterodimer first undergoes spontaneous dissociation in darkness, followed by NES-mediated active nuclear export of free CRY2PHR-mCherry to the cytoplasm, while CIBN-GFP remains nuclear due to its dual NLS. The observed kinetics are consistent with published CRY2PHR/CIBN dissociation parameters, confirming the dynamic and reversible nature of this

optogenetic switch.[3, 4] Dark controls showed negligible spontaneous translocation throughout the observation period. These functional validation experiments confirm that our paCas13d system operates through the well-established CRY2PHR/CIBN photocycle, providing wavelength-specific, and reversible optogenetic control over Cas13d activity.

Changed in the "Supplementary Information" (Supplementary Fig. 1)

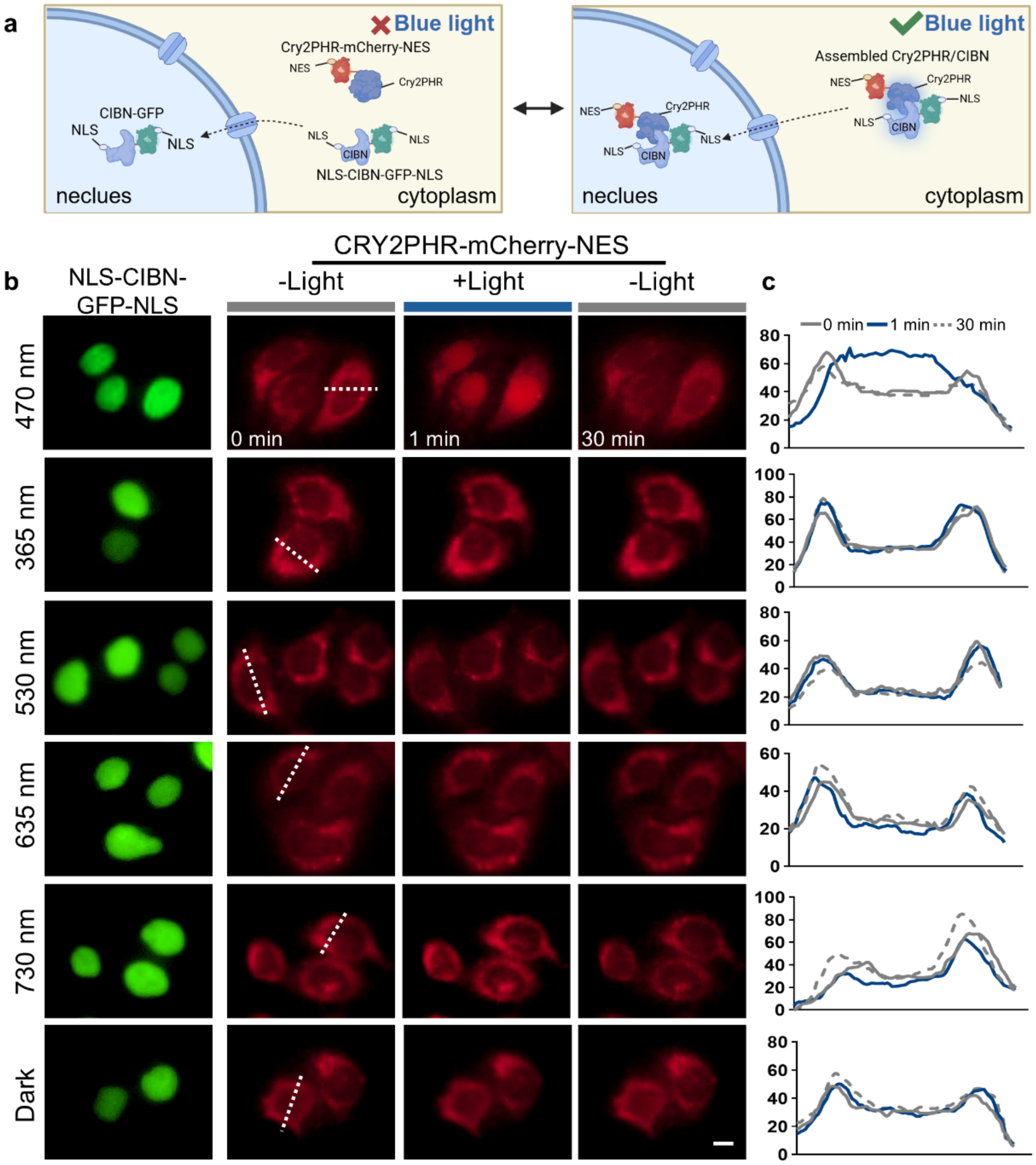

Supplementary Fig. 1 Wavelength specificity and reversibility of CRY2PHR/ CIBN light-inducible dimerization

(a) Schematic illustration of the nucleus-cytoplasm translocation assay. Nuclear-localized NLS-CIBN-GFP-NLS (green) and cytoplasmic CRY2PHR-mCherry-NES (red) undergo heterodimerization upon blue light (470 nm) illumination, inducing

CRY2PHR-mCherry nuclear translocation. NLS: nuclear localization signal; NES: nuclear export signal. **(b)** Representative live-cell imaging of HEK293T cells expressing NLS-CIBN-GFP-NLS and CRY2PHR-mCherry-NES exposed to different wavelengths (470 nm, 365 nm, 530 nm, 635 nm, 730 nm) or maintained in darkness (Dark). Left column: NLS-CIBN-GFP-NLS nuclear localization. Middle columns: CRY2PHR-mCherry distribution before illumination (−Light, 0 min), 1 minute after light onset (+Light, 1 min), and 30 minutes after light cessation (−Light, 30 min). Scale bar, 10 μm. **(c)** Quantitative line scan analysis of CRY2PHR-mCherry fluorescence intensity over time for each wavelength condition shown in **(b)**.

(2) Based on my previous comments, the authors have now presented the emission spectra of UCNPs-PEI [Fig. R2a (Fig. 2g)] and ligand-free UCNPs [Fig. R2b (Supplementary Fig. 4a)]. They assert that, "As shown in Figure R2a and R2b, both samples exhibit a dominant blue emission band centered at ≈470 nm, consistent with Tm^{3+}-sensitized upconversion, indicating that PEI surface modification does not change the emission wavelength profile." However, the emission spectra have clearly changed; the emission peaks observed in the UV region and around 450 nm for UCNPs are completely absent in UCNPs-PEI. An explanation for this reason is required.

Response: We thank the reviewer for the careful reading and for pointing out this important nuance. We agree that, beyond the dominant blue band centered at ~470-475 nm, the relative contributions of minor high-energy features (UV region and ~450 nm) differ between ligand-free UCNPs and UCNPs-PEI. Importantly, these UV and ~450 nm emissions are generally assigned to higher-lying Tm^{3+} excited states that are populated through higher-order multiphoton upconversion pathways (e.g., 349/362 nm and ~450 nm bands), whereas the ~470-475 nm band corresponds to a lower-order pathway and is typically more robust. This hierarchy makes the UV and ~450 nm features intrinsically weaker and more sensitive to near-surface effects, so modest increases in nonradiative losses can preferentially suppress them while leaving the ~470-475 nm band largely preserved.[5] Mechanistically, surface-related quenching is well known in lanthanide-doped UCNPs: high-frequency vibrational oscillators (notably O–H/N–H/C–H modes from surface ligands, polymer coatings, and associated hydration layers) can accept excitation energy via multiphonon processes or vibration-assisted pathways, enhancing nonradiative relaxation, which disproportionately impacts high-energy emitting levels.[6] In our system, PEI functionalization introduces abundant N–H/C–H vibrational modes in close proximity to the lanthanide ions and changes the interfacial solvation structure, which can increase surface-mediated nonradiative losses and thus suppress the more fragile UV and ~450 nm components.

Therefore, our revised interpretation is that PEI coating preserves the dominant blue emission band near ~470 nm, while minor higher-energy bands are relatively attenuated due to enhanced surface-associated nonradiative relaxation that selectively affects higher-order multiphoton channels. Accordingly, we have revised the manuscript text to avoid an overgeneralized statement.

Changed in the "Revised manuscript" (Lines 263-266):

Under 980 nm excitation, both ligand-free UCNPs (before PEI modification) and UCNPs-PEI (after PEI modification) exhibited a dominant blue emission band centered at ~470 nm (Fig. 2g and Supplementary Fig. 4a). Direct comparison under identical acquisition settings shows that PEI functionalization preserves the dominant ~470 nm blue emission, although minor high-energy features in the UV/~450 nm region are relatively attenuated.

Reviewer #2 (Remarks to the Author):

The authors have adequately addressed all concerns raised. They have performed additional experiments and text alterations that bring added clarity to the developed system and its purported mechanism of action and future therapeutic application.

I have no further questions

Response: We greatly appreciate the Reviewer's positive assessment and thoughtful comments throughout the review process.

References

1.Zetsche B, Volz SE, Zhang F. A split-Cas9 architecture for inducible genome editing and transcription modulation. *Nat Biotechnol* **33**, 139-142 (2015).

2.Fornerod M, Ohno M, Yoshida M, Mattaj IW. CRM1 is an export receptor for leucine-rich nuclear export signals. *Cell* **90**, 1051-1060 (1997).

3.Kennedy MJ, Hughes RM, Peteya LA, Schwartz JW, Ehlers MD, Tucker CL. Rapid blue-light-mediated induction of protein interactions in living cells. *Nature methods* **7**, 973-975 (2010).

4.Taslimi A*, et al.* An optimized optogenetic clustering tool for probing protein interaction and function. *Nature communications* **5**, 4925 (2014).

5.Liu X*, et al.* Probing the nature of upconversion nanocrystals: instrumentation matters. *Chem Soc Rev* **44**, 1479-1508 (2015).

6.Chen G, Qiu H, Prasad PN, Chen X. Upconversion nanoparticles: design, nanochemistry, and applications in theranostics. *Chem Rev* **114**, 5161-5214 (2014).
